# A New Standardized Stimulus Set for Studying Need-of-Help Recognition (NeoHelp)

**DOI:** 10.1371/journal.pone.0084373

**Published:** 2014-01-07

**Authors:** Aenne A. Brielmann, Margarita Stolarova

**Affiliations:** 1 Department of Psychology and Zukunftskolleg, University of Konstanz, Konstanz, Germany; 2 Faculty of Society and Economics, Rhine-Waal University of Applied Sciences, Kleve, Germany; University of Bologna, Italy

## Abstract

This article presents the NeoHelp visual stimulus set created to facilitate investigation of need-of-help recognition with clinical and normative populations of different ages, including children. Need-of-help recognition is one aspect of socioemotional development and a necessary precondition for active helping. The NeoHelp consists of picture pairs showing everyday situations: The first item in a pair depicts a child needing help to achieve a goal; the second one shows the child achieving the goal. Pictures of birds in analogue situations are also included. These control stimuli enable implementation of a human-animal categorization task which serves to separate behavioral correlates specific to need-of-help recognition from general differentiation processes. It is a concern in experimental research to ensure that results do not relate to systematic perceptual differences when comparing responses to categories of different content. Therefore, we not only derived the NeoHelp-pictures within a pair from one another by altering as little as possible, but also assessed their perceptual similarity empirically. We show that NeoHelp-picture pairs are very similar regarding low-level perceptual properties across content categories. We obtained data from 60 children in a broad age range (4 to 13 years) for three different paradigms, in order to assess whether the intended categorization and differentiation could be observed reliably in a normative population. Our results demonstrate that children can differentiate the pictures' content regarding both need-of-help category as well as species as intended in spite of the high perceptual similarities. We provide standard response characteristics (hit rates and response times) that are useful for future selection of stimuli and comparison of results across studies. We show that task requirements coherently determine which aspects of the pictures influence response characteristics. Thus, we present NeoHelp, the first open-access standardized visual stimuli set for investigation of need-of-help recognition and invite researchers to use and extend it.

## Introduction

It is a challenge to construct research in such a way that enables valid conclusions to be drawn about what particular manipulations lead to distinct outcomes. One of the crucial steps in planning meaningful experiments is to choose appropriate stimulus material. Scientific research benefits from access to pre-tested standardized stimulus sets since they provide information about the properties of the stimuli and their characteristics as perceived by an average population. In this way, standardized stimuli decrease the likelihood of confounding effects of experimental manipulations with those of irrelevant stimulus dimensions. If used repeatedly, standardized stimuli can increase knowledge over and above the insights provided by single experiments [1], [2]. With regard to visual stimulus material, at least two dimensions need to be considered: stimulus content (e.g. which object or situation are depicted, how they are illustrated), and perceptual stimulus properties (such as luminance, [3], color [4] and spatial frequency, e.g. [5]). Mostly, researchers are interested in attributing differences in outcomes to only one of those two dimensions. Yet, these are often confounded and changes in one can lead to unintended effects in the other. It has been noted specifically for ERP studies that one should “never assume that a small physical stimulus difference cannot explain an ERP effect” ([6], p. 74), and these findings can be extended to other methodological approaches too. In EEG, fMRI and in eye movement studies, picture properties, such as complexity, e.g. [7], contrast, e.g. [8], [9], intensity, e.g. [10], and color (e.g. [4]; see also [11] for a review) have been shown to profoundly influence participants' responses. Thus, when comparing experimental outcomes with regard to stimulus content, it is important to show that results do not relate to systematic perceptual differences between stimulus categories, e.g. [6], [12]. At the same time, researchers need to ensure that the control of perceptual properties does not compromise construct validity or recognizability of the stimuli used.

Many standardized stimulus sets exist for objects, e.g. [13]–[15], as well as for scenes [16], [17], faces, e.g. [18], and other categories of stimulus material [19]–[21]. Some have been used for years and have helped advance research theories on emotional perception, e.g. the IAPS [2], [22], and methods, e.g. regarding morphing algorithms for face pictures [23]. So far, however, no standardized set of material that encompasses stimuli relevant for social behavior exists. Some recent studies investigating the development of theory of mind (ToM) have used comics as stimulus material in brain imaging studies [24]–[26]. Other picture sets have been employed for evaluating deficits in social emotional behavior, self-concepts [27], [28], expression of feelings [29], autism [30], and schizophrenia [31]. Unfortunately, only limited information on perceptual properties and no normative base line data are available for these stimuli, even though some of them have been used repeatedly in different studies [24], [25]. Another major limitation of existing material concerns construct validity: Visual stimuli depicting social situations have rarely been pre-tested with regard to clarity of illustration, easiness of categorization or general understandability.

It was our goal to provide the research community with an open-access standardized set of visual stimuli, overcoming the methodological limitations mentioned above and extendable to meet different research needs. Socioemotional abilities have been shown to undergo constant development throughout childhood, well into adolescence (e.g. see [32] for five to 14-year-olds; [33], [34] for a review considering ages three to 8; [35] for a longitudinal study). Active helping, a kind of pro-social action and a possible and desirable consequence of need-of-help recognition, has been assessed extensively in studies relying on real-life social interactions (e.g. [36], [37], see also [38] for a book chapter summarizing relevant studies). In recent years, developmental psychology has seen a rise in the assessment of active helping in infants and toddlers documenting children's early willingness and ability to help [39]–[44]. While it can be assumed that when helping occurs, the need-of-help has been recognized, the reverse inference cannot be made: There are many reasons not to help and one of them is to not have realized that someone needed help. Assessing when and under what circumstances humans are able to identify need-of-help will increase our understanding of social behaviors, the ontogeny of helping and potentially of factors endangering prosocial development.

Investigating need-of-help recognition is thus a worthy research path, that has so far received little attention, possibly partially due to the lack of successful experimental operationalization and accessible stimulus material. We created pictures of situations understandable for children of different ages as well as for adults and clinical populations, i.e. simple comic-like black-and-white line drawings. These drawings depict 15 harmless and well-known everyday situations. Variations of each situation, either depicting somebody in need of help or not, were created in a way that ensured maximum low-level perceptual similarity. The intended pair-wise picture similarity was objectively tested by calculating structural similarity indices [45]. Herewith we ensured that confounding effects of perceptual differences are unlikely when using the NeoHelp stimulus set, making it suitable for various brain imaging and psychophysiological studies.

In order to enable the application of a control task that requires only basic human-animal distinction, picture variations showing a bird in the same situation as the human were also created, while maintaining maximum perceptual similarity. This control category was employed here in order to enable the distinction of general categorization abilities (i.e. distinguishing a bird from a human) and developmental status (as manifested for example in response times across different tasks) from specific help-content related outcomes (e.g. distinguishing someone in need of help from someone who doesn’t require assistance). This is especially relevant when general perceptual and differentiation abilities cannot be assessed in depth.

Thus, in order to assess whether children of different ages are able to differentiate the picture content according to both – species (humans and birds) and need-of-help depiction –, we created three mutually controlling behavioral experiments. Two of the employed experimental designs (P2 and P3) assessed children's need-of-help recognition abilities. They varied in presentation duration and decision mode. In a control task (P1) children were asked to differentiate between humans and birds disregarding the need-of-help content. Herewith P1 assessed differences in general categorization abilities and motivation. Importantly, P1 employed the identical stimuli as P2 and P3. Presentation and decision mode were the same as in one of the two help-content related paradigms. In all three paradigms we recorded accuracy (in terms of hit-rates) and speed of response. Comparing these behavioral correlates across the three paradigms enabled an evaluation of the NeoHelp's usability for assessing need-of-help recognition abilities. Specific need-of-help recognition related response patterns would be an indication that the NeoHelp stimulus set is a useful research tool in assessing and quantifying need-of-help recognition abilities and relating them to developmental characteristics and situational factors.

The results presented here were not intended to shed light on questions regarding child or situation related factors influencing need-of-help recognition – this is the subject of a separate analysis by the authors (Stolarova & Brielmann, under review). Before answering help-content related questions, we needed to determine whether the stimuli employed fulfilled the perceptual and content requirements necessary, i.e. perceptual similarity of the stimuli, as well as general clarity of content according to both, species (here: human vs. bird) and need-of-help-related categories. Thus, this report focuses on the NeoHelp-stimuli, their perceptual properties and their suitability for use in empirical studies with children.

We provide evidence that the NeoHelp stimuli are highly similar regarding low-level picture properties and thus are unlikely to cause confounding effects of content and stimulus properties. With the experimental study presented here, we provide baseline values for response accuracy (hit rates) and response times (RTs) for the NeoHelp stimulus set (see Appendix S1). These results show that the NeoHelp stimuli are reliably categorized by a diverse group of children both according to help-content as well as to species categories. They demonstrate that different need-of-help-distinction and control human–bird differentiation tasks yield different response and picture property effect patterns. We conclude that the NeoHelp stimulus set fulfills the requirement of standardized visual stimuli and will aid research on need-of-help recognition in the broader domain of socioemotional development. Because of its strictly controlled low-level perceptual properties the NeoHelp stimulus set is well suited for future research suing EEG and fMRI as well as for behavioral testing as presented here. It will enable researchers to tackle different questions related to social development and prosocial behavior, e.g. the question whether children's (lack of) helping behavior is attributable to their ability to realize somebody's need-of-help or how specific situational or child-related factors influence children's ability to recognize another person's need-of-help. Thus, we make our NeoHelp available to all researchers and provide information on perceptual properties of and response characteristics to each stimulus.

## Methods

### Ethics statement

All parents gave written informed consent according to the principles of the Declaration of Helsinki (see: http://www.wma.net/en/20activities/10ethics/10helsinki/) before their children participated in this study. Special care was taken to ensure that both parents and children understood that their participation was voluntary and could be ended at any time without disadvantage to the participants. The research reported here was conducted in Germany (country of residence of both authors) and is in accordance with the Ethic Guidelines of the German Psychological Association and the German Psychological Professional Organization (Ethische Richtlinien der Deutschen Gesellschaft für Psychologie e.V. und des Berufsverbands Deutscher Psychologinnen und Psychologen e.V., http://www.bdp-verband.org/bdp/verband/ethik.shtml), an approved German adaption of the “Ethical Principles of Psychologists and Code of Conduct”, *American Psychologist*, 2002, *57*, 1060–1073. Formal approval from an ethics committee is not required at the University of Konstanz for non-invasive studies involving human participants such as this.

### Stimuli

**Figure 1:**
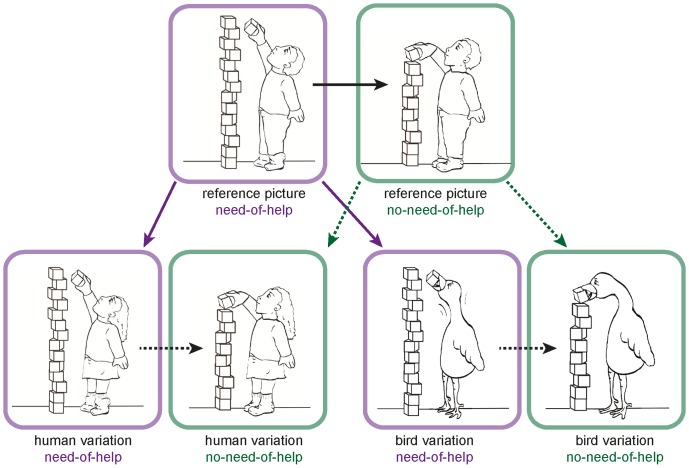
Example situation and picture generation process. The situation “blocks” with all its variations is shown here as an example. Pictures framed in purple illustrate the need-of-help depictions; pictures framed in green illustrate the no-need-of help variations. Solid arrows indicate from which picture variations were derived by changing as little lines as possible. Dashed arrows indicate which pictures were combined to generate variations in no-need-of-help pictures (here a bird variation and a variation of gender): Single features were transferred from the picture variation to the no-need-of-help reference picture.

The NeoHelp stimulus set consists of 82 black-and-white drawings (600px x 800px) that show children of different ages and sexes in 15 everyday situations. Figure 1 shows one example situation and the variations derived from the reference picture (see Appendix S2 for a detailed description of stimulus generation and Appendix S1 for an illustration of all situations). For each of the 15 situations we created corresponding picture pairs displaying birds in analog situations. These control stimuli were intended to enable implementation of a basic human-bird distinction, allowing us to disentangle general effects of categorization abilities from specific help-content related effects. Birds were shown in the same situations as humans to ensure maximum low-level picture similarity. It was achieved by altering only those lines of the reference picture necessary to generate the corresponding picture variation. In order to increase the number of possible trials per situation, without repeating identical pictures, we also derived variations for 10 out of 15 human situations. Table 1 lists all 15 situations with their variations (for a complete listing and illustrations see Appendix S1). In total, there were 41 different pictures pairs (NoH – no-NoH) for 15 situations.

**Table 1:** List of situations in the stimulus set, their variations and number of pictures.

Situation	NoH-depiction	No-NoH-depiction	Variations derived	Number Of Pictures
Blocks	Try to put the last block on top of a tower	Build a tower with blocks	Bird, gender	6
Boat	Try to reach sth. in the water	Catch sth. out of the water	Bird, gender	6
Branch	Be stuck on a twig	Walk past a branch	Bird, gender	6
Climb	Try to climb a table	Stand in front of a table	Bird	4
Door	Try to reach door knob	Open a door	Bird	4
Gap	Try to cross a large gap	Step over a small gap	Bird, gender	6
Shelf	Try to reach a ball inside a shelf	Grasp a ball inside a shelf	Bird, ethnicity	6
Shirt	Try to take off shirt	Stand	Bird	4
Sit	Reach out to be lifted up	Sit on the floor	Bird	4
Stair	Try to reach top of stair step	Climb up a step of a stair	Bird, gender	6
Table	Try to reach ball on table	Grab ball from table	Bird	4
Table_chair	Try to reach a ball on a table	Grasp a ball on a table	Bird, age	8
Apple*	Try to reach an apple on a tree	Pick an apple	Bird, gender	6
Bucket*	Try to lift a heavy bucket	Put sth. into a bucket	Bird, gender	6
Drawer*	Try to open a drawer	Open a drawer	Bird, gender	6
Total				82

Variations refer to: gender  =  boy and girl; ethnicity  =  African and Western European decent; age  =  kindergarten age and toddler. Note that the age variation of “table_chair” also had a separate bird control picture pair. *Situations that were only analyzed if presented in smaller size and thus in the supplementary material.

### Technical apparatus and stimulus presentation

**Figure 2:**
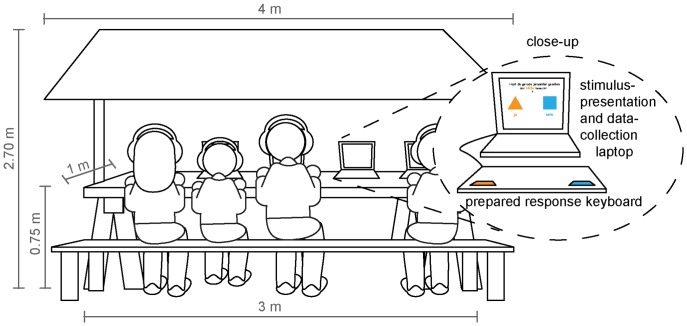
Setting for the experiment. The stand was located outside as part of a child-directed family fair. A maximum of five children could participate at any given time. A laptop collecting responses and showing stimuli on its screen, a keyboard (with color-coded response keys and a cupboard covering task-irrelevant keys) and a set of headphones were assigned to each child.

Stimuli were presented on the screens of customary laptops using Presentation (version 16.0) software. Identical regular keyboards were adapted for use as response devices. All the keys were covered with a cupboard contraption, except for the two response keys, which were laminated and color coded according to the experimental presentation (see Fig. 2). Color codes were counter-balanced for left and right responses across different PCs. Children were assigned to the different PCs randomly. Due to the restrictions of screen resolution and size, 12 out of 15 situations were presented correctly (stimulus presentation size: max. visual angle  = 15.62° picture height) on four out of five laptops. Data for all 15 situations was obtained on one laptop only (max. visual angle  = 11.75° picture height) and is reported in the supplementary materials (Appendix S3) because of the relatively small sample size (N = 22) and thus preliminary nature of the results.

### Experimental design

**Figure 3:**
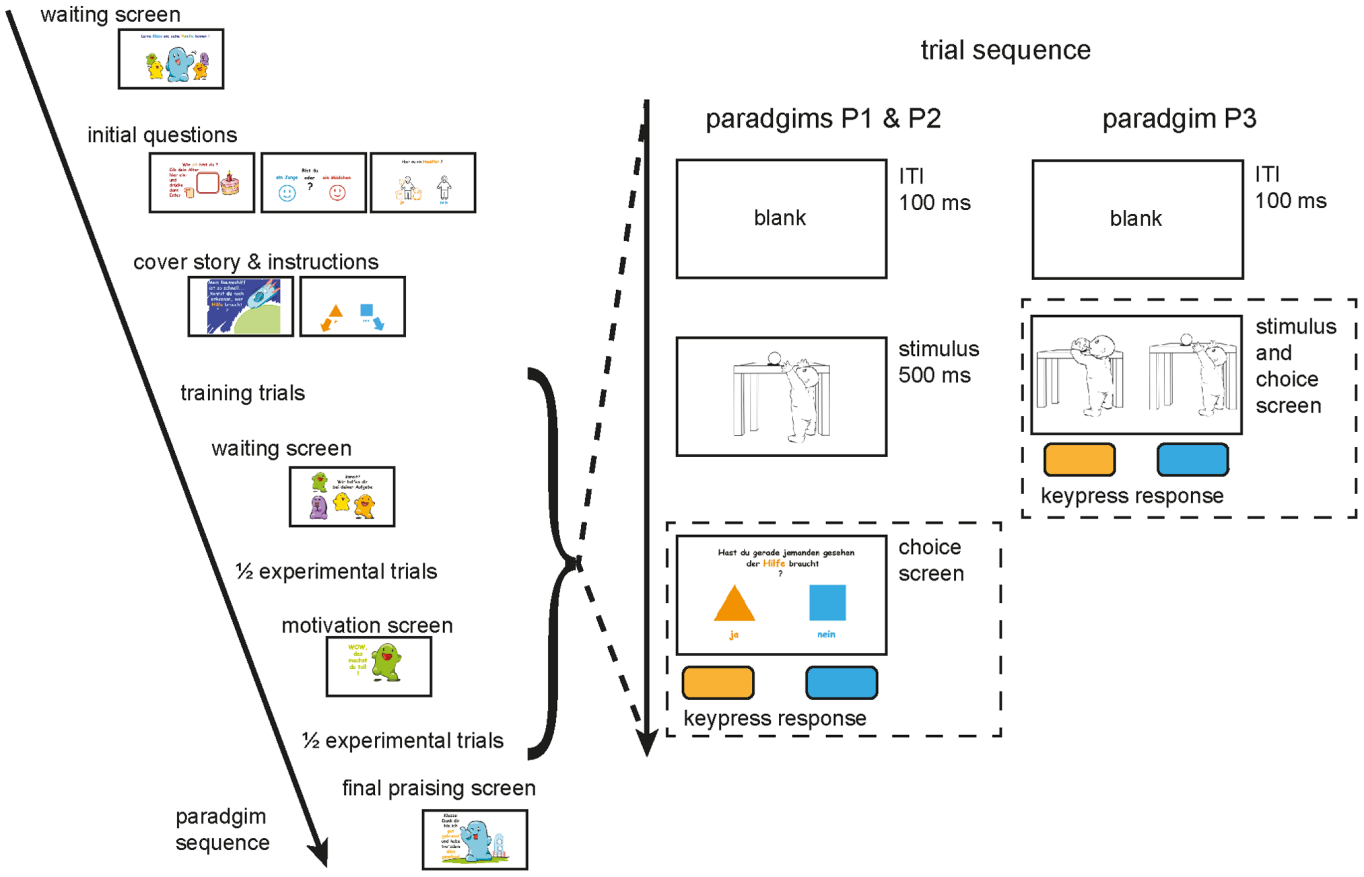
Experiment design variations. The overall paradigm sequence shown on the left side was the same for all three paradigms. The right side of the figure illustrates trial sequences, which were always the same for training and experimental trials. Note that P1-bird and P2-help had identical time restricted stimulus presentations (500 ms), trial sequence, randomization routines and experimental designs (2AFC); decision and response took place after stimulus off-set. Only introduction and instruction differed between P1-bird and P2-help: In P1-bird children were asked to indicate whether the picture showed a human or a bird, while in P2-help they were asked whether the picture showed someone in need-of-help (NoH) or no-NoH. In P3-help, on the other hand, stimuli were presented pair-wise, presentation was continued as long as needed for the child to respond, and thus the decision was taken during stimulus presentation.

The experimental design consisted of three separate paradigms united through a child directed cover story: P1-bird, P2-help and P3-help-side (see Fig. 3 for a detailed summary). In all paradigms, the order of picture presentation was random, as was the selection of the pictures for the training phase. In order to ensure comparability between paradigms, all 82 pictures were presented in each paradigm once, regardless of whether they had been used during the training trials. Children were always instructed to respond as fast and as accurately as possible and were motivated to do so by an elaborate cover-story (see SupplementaryMethods S2).

P1-bird- and P2-help had identical two-alternatives-forced-choice (2AFC) task setups and differed only in terms of instructions. In the P1-bird control task, children were asked after the stimulus off-set to indicate whether they had just seen a bird or a human. In P2-help, the identical stimulus presentation was followed by the question of whether children had seen someone (bird or human) in need-of-help (NoH) or not. Each trial of the two paradigms P1-bird- and P2-help consisted of a 100 ms inter stimulus interval (ITI), 500 ms stimulus presentation and a decision screen, where participants were asked to make a 2AFC-response (see Fig. 3). Paradigm P3-help-side was a pair-wise picture-selection-task without time restrictions. Trials were preceded again by an ITI of 100 ms. Afterwards a no-NoH/NoH pair (humans or birds) was presented on the screen until the child made a response. Children were asked to indicate which one of the two pictures showed someone in need of help by pressing a corresponding button.

Paradigms were designed to allow differential assessment of which task demands caused distinct behavioral effects: The comparison of the results from P2-help and P1-bird differentiated the influences of basic categorization abilities (and thus of general developmental characteristics such as speed of processing) from those of stimulus presentation and response mode. The comparison of P2-help and P3-help-side differentiated influences of task load through time restrictions from those of the NoH-recognition. Thus, any effect that emerges in both help-related paradigms (P2-help and P3-help-side) and not in P1-bird can be attributed to the need-of-help recognition task demand.

The order of paradigm P1-bird and P2-help presentation differed on the different PCs. P3-help-side was always conducted last, since the kind and duration of presentation made familiarization with the stimuli more likely. This procedure resulted in two randomly assigned order variations: 1) P1-bird, P2-help and P3-help-side and 2) P2-help, P1-bird and P3-help-side.

### Experimental procedure

All three paradigms started with introduction screens explaining the cover story (a detailed description is provided in Appendix S2). Further screens prompted responses regarding the child's participant number, his/her age and whether or not s/he had any pets (see Fig. 3). These introductions were directed by an experimenter, who typed the responses and administered a minimum of three training trials before starting the actual experiment. Four trained experimenters were present at all times. All children wore headphones during the experiment to reduce distracting noise and to be able to hear applause for encouragement.

Depending mainly on the age of the children, total testing time per paradigm, including instruction and training, varied between 4 and 12 minutes. Children were free to end the session at any time. If the experimenter came to the conclusion that the requirements of the task far exceeded the children's ability (mostly because of young age, e.g. below 4 years), they suggested completing only P3-help-side, where pictures were presented as long as was necessary for the child to react.

### Participants and exclusion criteria

Data collection took place at an open-air child directed festival on September 8th, 2012 in the German city of Konstanz (see Figure 2 for an illustration of the experimental setting). It can be assumed that the vast majority of participating children either lived in one of the two bordering towns Konstanz (in Germany) and Kreuzlingen (in Switzerland) or their surroundings. No child was refused participation; no intentional pre-selection of any kind took place. At least one paradigm on one of the four laptops showing the larger stimuli (see “Technical apparatus and experimental procedure”) was begun by 68 German-speaking participants during the course of the whole day, which began at 11 a.m. and finished at 5 p.m. An additional 22 children were randomly assigned to a laptop showing smaller pictures (see Appendix S3).

**Figure 4:**
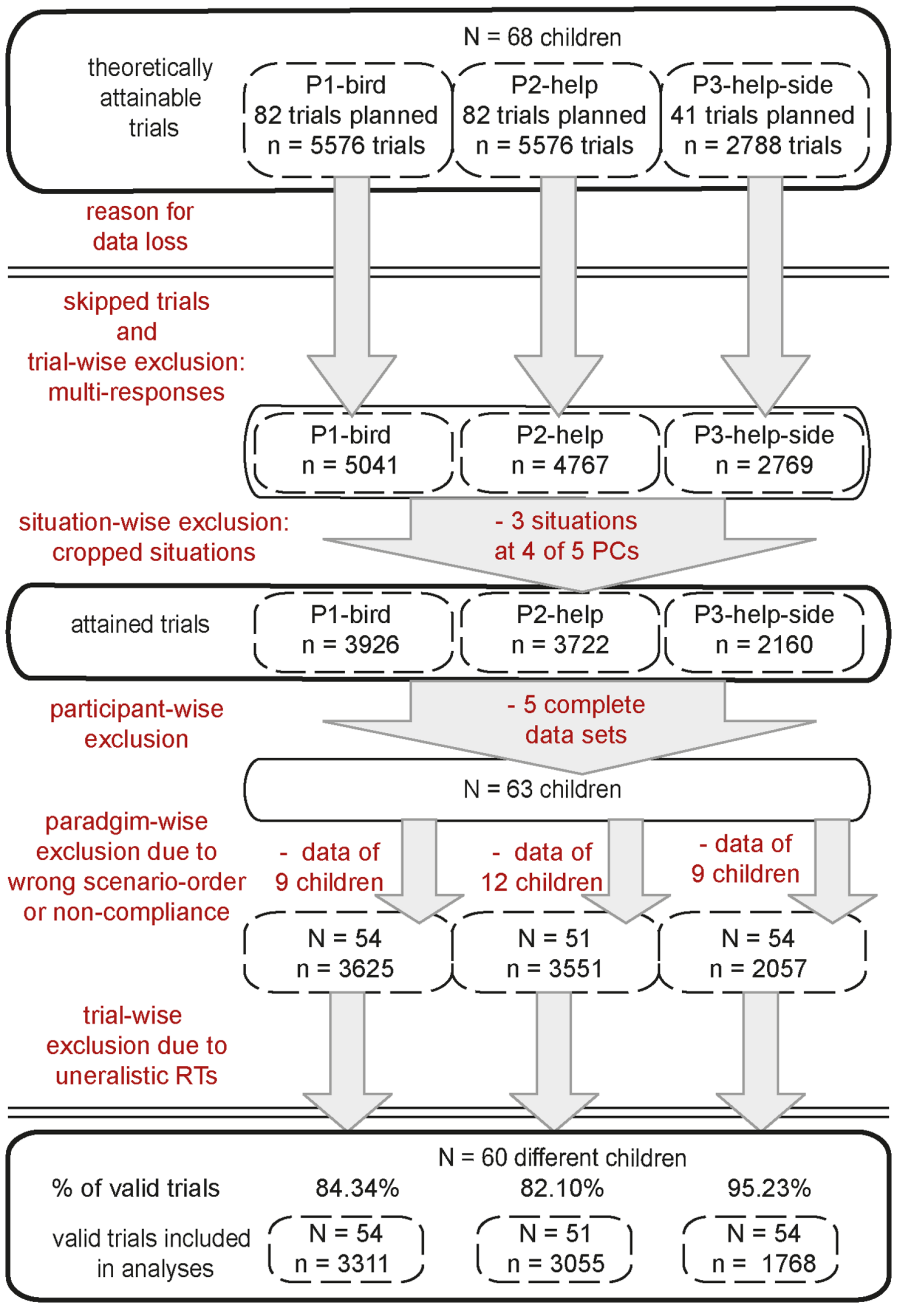
Exclusion procedure and data cleansing. (N  =  number of different children from whom data is considered; n =  number of trials). “Skipped trials” were the result of children beginning but not finishing a paradigm. “Multi-responses” refers to exclusion of all trials for which more than one key press was recorded. The “cropped situations” were “apple”, “bucket” and “drawer”; due to technical difficulties their presentation on 4 out of 5 laptops was impaired. “Participant-wise exclusion” occurred because of technical difficulties with the equipment (2), parental interference (1), because children had already done the experiment in a pretest-setting (2) or because of significant cognitive delays (1). “Wrong paradigm order” occurred if (due to experimenter's error) children completed the P3-help-side paradigm before any of the other two paradigms. We defined “non-compliance” as being the case when a paradigm's mean hit rate was below 55%, mean RT was above 4 s or below 300 ms. RTs were considered “unrealistic” if they were above 4 s, below 100 ms or 4 SDs apart from a child's mean RT. The “% of valid trials” refers to the proportion of attained trials that has been used for analyses.

**Figure 5:**
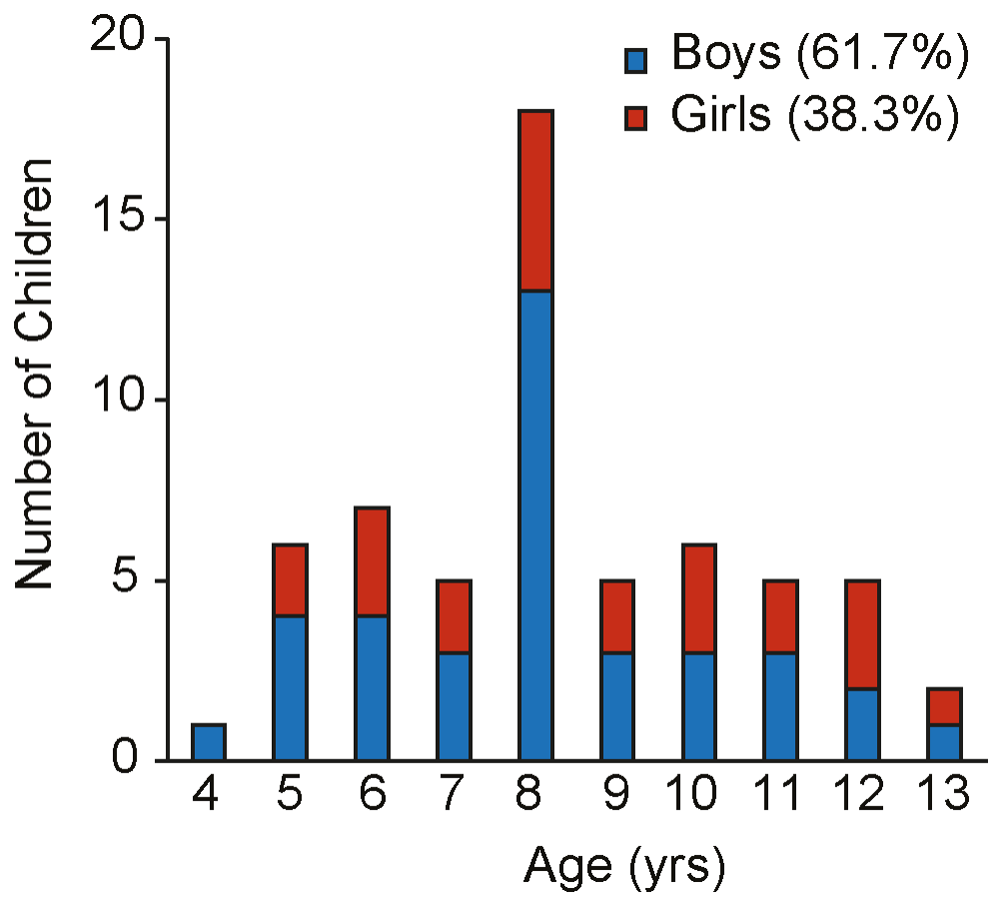
Number of children whose data was used for analyses. Red proportions of bars represent the number of girls, blue proportions represent the number of boys.

A summary of the exclusion criteria and the complete data cleansing procedure, including the number of trials for each of the three paradigms at any given step, is provided in Figure 4. Data on 60 children (37 boys, mean age  = 8.35 yrs, SD = 2.25) was obtained for 12 NeoHelp situations presented in a larger size. The age distribution for each gender is illustrated in Figure 5. Because of the paradigm-wise exclusion criteria and because not all children completed all three paradigms, data from slightly different sub-samples was available for the analysis of each paradigm. Details regarding concrete trial numbers are provided at the beginning of each analysis. In addition, we analyzed data for all 15 NeoHelp-situations, obtained from those 22 children (14 boys) who through random assignment saw them in smaller size. We present a summary of those results in the Appendix S3, in order to aid the readability of this report.

### Analysis strategy

All statistical analyses were conducted with GNU-software R (version 3.0.0). We assessed whether the newly created stimulus set was suitable for investigating need-of-help recognition in children. Thus, emphasis was put on the perceptual properties of the stimuli as well as on the detection of ambiguous and thus potentially unsuitable stimuli. We also explored which picture properties influence response characteristics in NoH-distinction tasks. For reasons of completeness, we provide analyses about whether human picture variations of a given situation or the sequence in which paradigms were absolved systematically influenced response characteristics in Appendix S4. Further exploration of significant interactions revealed by ANOVAs was done by computing Tukey honest significant difference (HSD) tests.

To evaluate the similarity between the pictures comprising a NoH-/no-NoH pair as well as between the variations of NoH-depictions, we calculated the structural similarity index (SSIM) between those pictures. SSIM was proposed as a measure of picture quality by quantifying the visibility of errors in a distorted picture. We employed it as a measure of picture similarity here because the comparisons made refer to differences between pictures in general and, as the authors put it, “it can be thought of as a similarity measure for comparing any two signals” [45].

Descriptive statistics were obtained using a Shapiro-Wilk-Test for normal distribution of age, as well as exact binomial tests (π = .50) on distribution of gender and pet-ownership. This paper focuses on the stimuli's suitability for experimental research.

In order to determine whether the situations of the stimulus set enabled children to distinguish between need-for-help (NoH) and no-NoH-pictures in P2- and P3-paradigm, exact binominal tests (π = .50) were conducted on hit rates per situation and across all situations. Analogue exact binomial tests were conducted for P1 in order to determine whether pictures of birds and humans were clearly discernible by children. Situations for which the estimated hit rate did not exceed chance were classified as “ambiguous”. Only unambiguously classifiable situations were included in further analyses.

In order to test whether different tasks can be applied to the identical NeoHelp stimuli, we quantified the effects of task demands on response characteristics (RTs and hit rates) using 12×3 (*situation* x *paradigm*) ANOVAs. The influence of specific picture properties on children's responses was assessed by means of four different factors: *situation, need-of-help-depiction (NoH-depiction), need-of-help-side (NoH-side)* and *bird-depiction*. Note that only one of the two factors *NoH-depiction* and *NoH-side* can be considered in each analysis, as *NoH-depiction* only applies to the paradigms P1-bird- and P2-help, while *NoH-side* only applies to P3-help-side. Three-way 12×2×2 ANOVAs were calculated, the first factor being *situation*, the second factor *bird-depiction*. The third factor was either *NoH-depiction* (for P1 and P2) or *NoH-side* (for P3).

Regarding mean RTs, we ran an additional analysis to investigate whether RTs were different for correct vs. incorrect responses. Therefore, 12×2 (*situation* x *correctness*) ANOVAs were computed for each paradigm separately. The relationship between hit rates and RTs of responses to different situations was clarified by testing significance of Pearson's correlation coefficients.

## Results

### Picture Similarity

The structural similarity index (SSIM, originally described in [45]) for NoH and no-NoH picture pairs ranged from 73% to 91% for human depictions, showing that objective picture properties were highly similar within these picture pairs. For bird depictions SSIM ranged from 68% to 95%, but was below 83% only for the situation “shelf”. Also, bird and human depictions were perceptually highly similar, as indicated by a SSIM ranging from 81% to 94%. What is more, the low SSIM for comparison between NoH-pictures and their variations demonstrates that objective similarity is also high within each situation: With one exception (the variation of ethnicity, SSIM  = 65%), SSIM for human variations ranged from 78% to 99%. Detailed SSIMs for each situation are listed in Appendix S1. In conclusion, low-level stimulus properties of the NeoHelp stimuli are highly similar across content categories and are thus unlikely to introduce effects confounded with those of picture content.

### Description of the participant population

In the sample of 60 children for which we will report detailed results age was not normally distributed, W = 0.72, p<.01 (see Fig. 5 for details of the age distribution), probably because of the overrepresentation of eight-year olds. Marginally more boys than girls participated, p = .09, and the majority of children did not own a pet, p = .03. Statistics on the comparisons of subgroups (60 children, who reported below and N = 22 children who were presented smaller pictures) are provided in the Appendix S3.

### Ambiguous situations

In the following analyses we investigated whether some of the situations were too ambiguous to be correctly categorized on average according to two different task-related contents: NoH vs. no-NoH in P2 and P3, human vs. bird in P1. Situations were defined as ambiguous when hit rates were not significantly above chance were and were subsequently excluded from further analyses of the specific paradigm's data. Tables in Appendix P1 summarize the results of these analyses.

**Figure 6:**
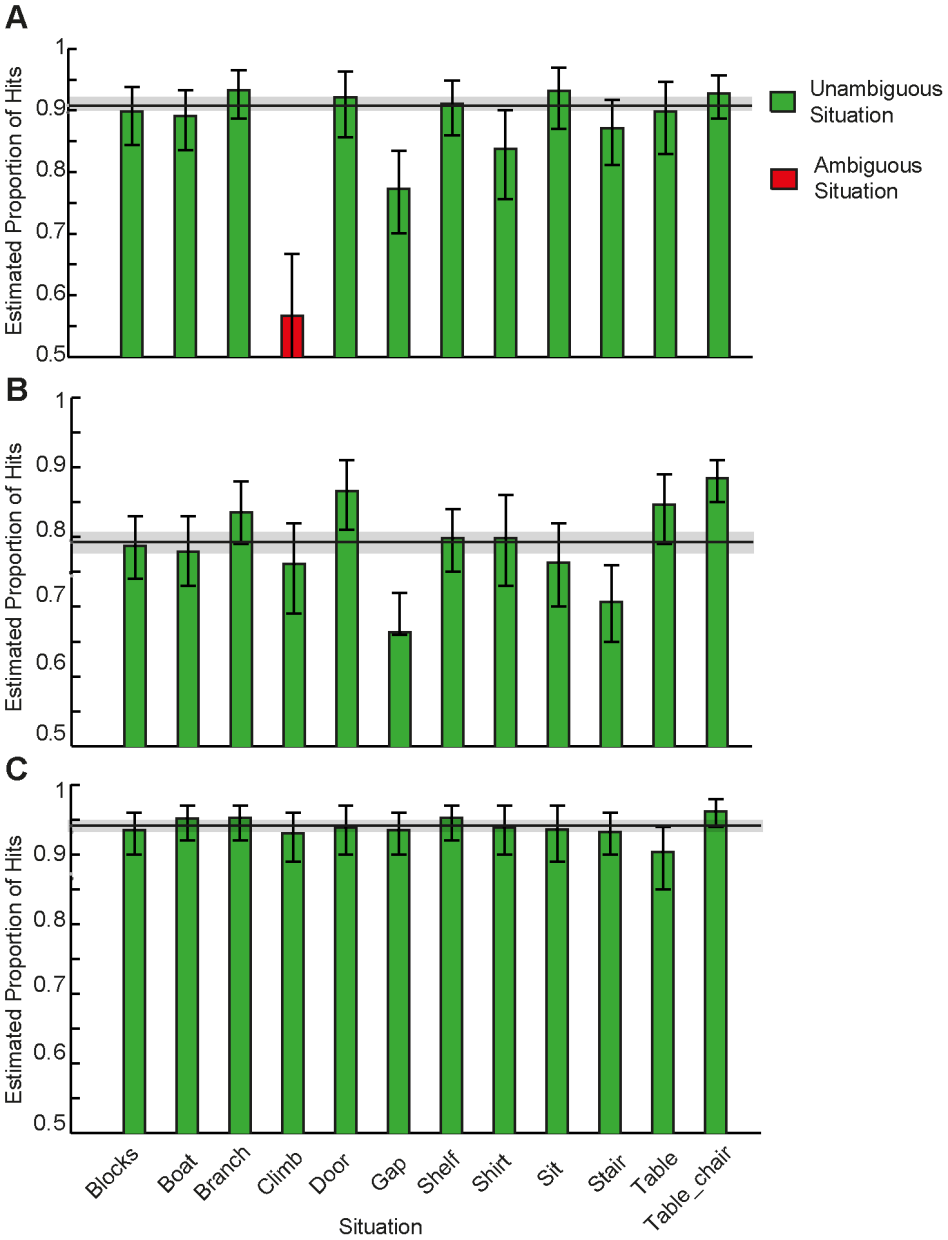
Estimated situation specific hit rates for P3-help-side (A), P2-help (B) and P1-bird (C). Estimates across variations of each situation are derived from tow-tailed exact binomial tests (π = .50). Green bars represent unambiguous situations (estimated hit rate significantly above .50) while red bars represent ambiguous situations (estimated hit rate not significantly different from chance). Error bars represent upper and lower bounds of the estimated hit rates' confidence intervals (CIs). Overall estimated hit rates were calculated across all unambiguous situations. Lines represent overall estimated hit rates, shaded areas surrounding them indicate their CIs.

Figure 6 A shows estimated probabilities of success in P3-help side, the open-ended direct no-NoH vs. NoH comparison. Pictures of all but one situation (“climb”, estimated hit rate  = 56.52%, p = .25) were identified correctly as depicting need-of-help (NoH) or no-need-of-help (no-NoH), all estimated hit rates >78.21%, all p<.001. Responses to “climb” therefore cannot be considered to reflect a correct distinction between NoH and no-NoH depictions when decision time is not restricted and a direct comparison between the NoH and no-NoH depictions takes place. Thus “climb” was excluded from further analyses of this paradigm's data, reducing the number of trials for analyses in P3-help-side from 1839 to 1676. Overall, estimated hit rate for P3-help-side across all unambiguous situations was 90.81%, p<.001, 95% CI [89.33%, 92.15%].

Figure 6 B shows estimated hit rates in P2-help, the NoH-/no-NoH-distinction after short stimulus presentation. Under the conditions of short picture presentation (500 ms) and responses made after stimulus off-set in P2-help, pictures of all 12 situations were correctly identified as showing NoH or no-NoH with a probability above chance, all estimated hit rates >66.30%, all p<.001. Note that these 12 situations also include the situation “climb” that was ambiguous concerning NoH-depiction in P3-help-side. Overall, the estimated hit rate across all situations was 79.25%, p<.001, 95% CI [77.77%, 80.67%], which is considerably above chance but also lower compared to P3-help-side.

Figure 6 C shows estimated hit rates in P1-bird, the human-bird-distinction after short stimulus presentation. Concerning the distinction between human and birds, there were no ambiguous situations; all estimated hit rates ≥90.34 %, all p<.001. The estimated overall hit rate for P1-bird was with 94.14%, p<.001, 95% CI [93.29%, 94.92 %], the highest one of all paradigms.

In sum, pictures of almost all situations (11 out of 12, marked with green outlines in all columns of Fig. 6 A-B) of the stimulus set were unambiguously identifiable as depicting need-of-help (NoH), regardless of presentation duration and task type (2AFC vs. picture selection). Children differentiated reliably between depictions of humans and birds for all 12 situations (see Fig. 6 C).

### Effect of specific task demands on hit rates and RTs

Hit rates as well as RTs were considerably influenced by *paradigm, situation* as well as the interaction of both factors (Table 2). Differences of response characteristics to distinct situations are described in detail in later analyses (see section “Effects of stimulus content on hit rates and RTs”). Here we report results concerning the factor *paradigm*.

**Table 2:** Results of ANOVAs investigating the influence of *paradigm* on hit rates and RTs.

		Hit Rates		RTs	
Source	df	F	p	F	p
Situation	11	8.92***	<.001	7.31***	<.001
Paradigm	2	185.10***	<.001	1633.28***	<.001
Situation x Paradigm	21	3.27***	<.001	3.15***	<.001

p<.001.

**Figure 7:**
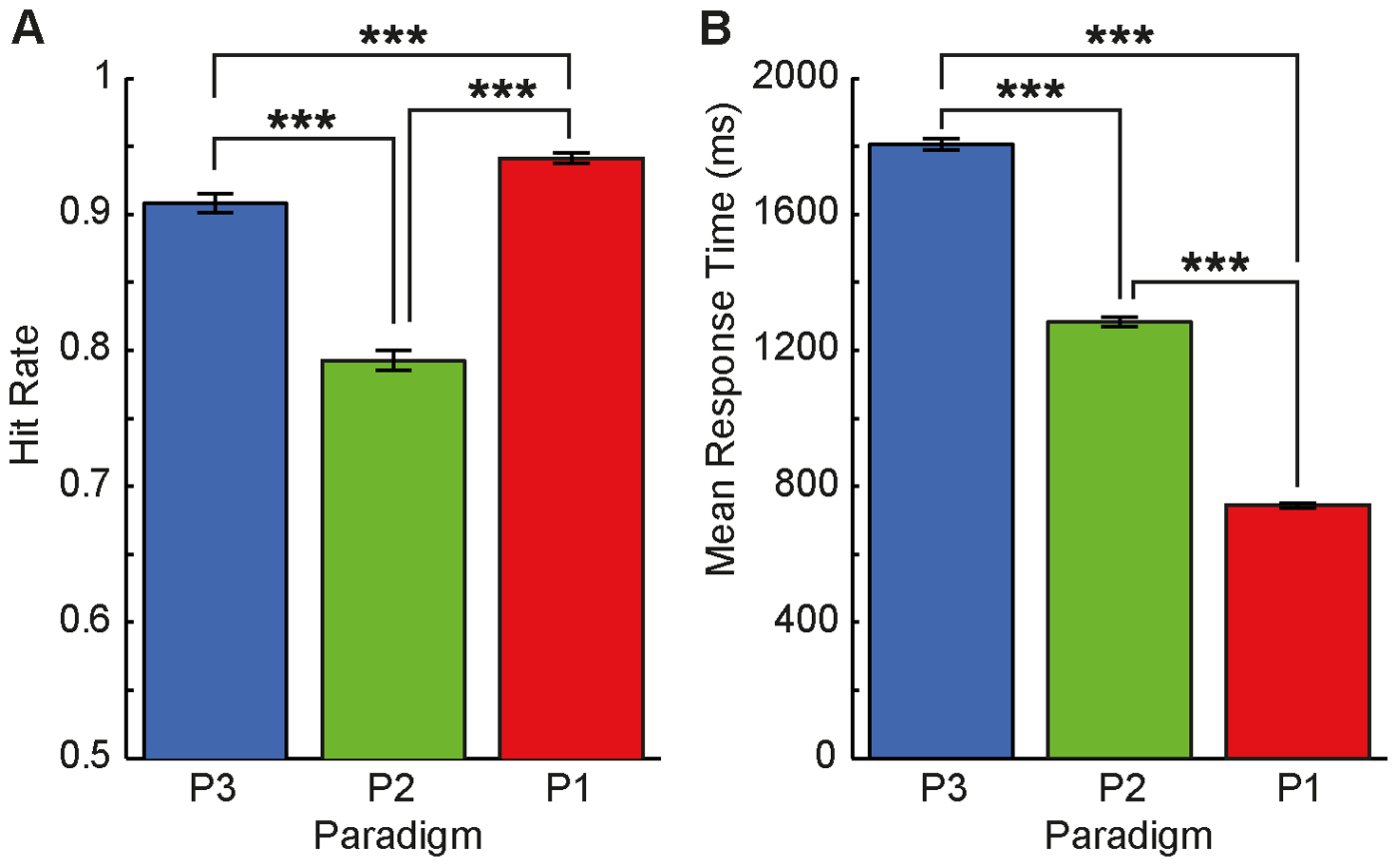
Comparison of hit rates and response times between paradigms. Hit rates and RTs are presented across situations. Asterisks mark significant differences revealed by post-hoc Tukey HSD tests, ***p<.001. Note that all differences between paradigms were significant. Error bars represent SEM.

**Figure 8:**
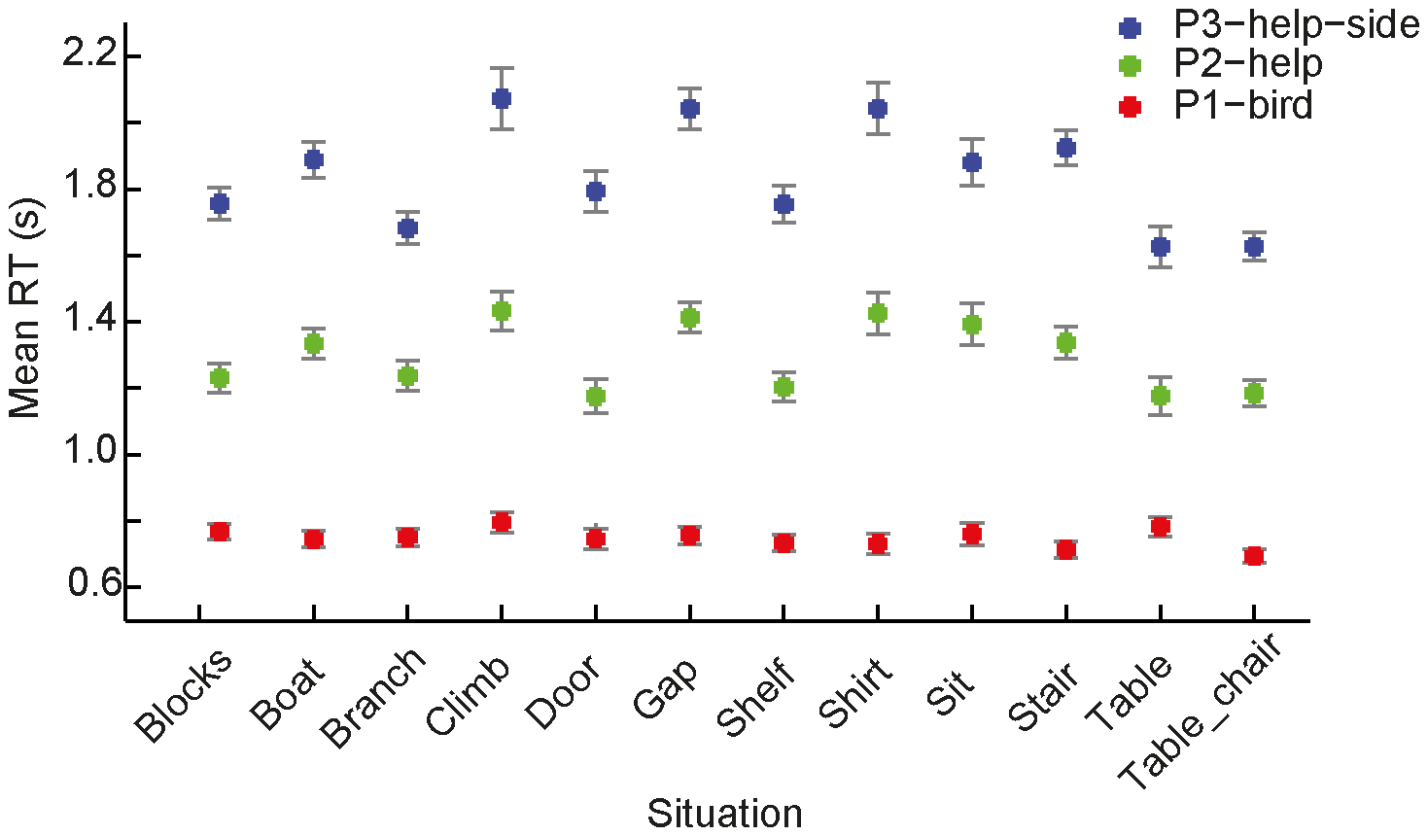
Interaction effect of *scenario* and *situation* on response times (RTs). Red symbols represent means for S1-bird, green symbols represent means for S2-help and blue ones represent means for P3-help-side. Note that differences between scenarios were not significant for all situations but that post-hoc tests confirmed that RTs were significantly different in all scenarios across situations (see Figure 5 for an illustration of the main effect). Error bars represent SEM.

As shown in Figure 7 A, mean hit rates were lowest in paradigm P2-help, illustrating the high task demands resulting from the need-of-help (NoH) distinction-task and the short picture presentation duration, combined with a decision from memory. We also found that mean hit rates were significantly higher in P1-bird (bird-human-distinction required) compared to P3-help-side. This finding indicates that the difficulty of the NoH-distinction task is so much higher compared to the bird-human-distinction task that it overrides the influence of stimulus presentation duration (non-restricted in P3-help-side, restricted to 500 ms in P1-bird) and decision mode (during paired comparison vs. from memory after stimulus offset) on response accuracy. Response times (RTs) were shorter for the two paradigms in which picture presentation was short (P1-bird and P2-help) compared to P3-help, where children saw two pictures until they made a response (see Figure 7 B) paradigm. What is more, RTs were also shorter if the task was to distinguish humans from birds rather than NoH- and no-NoH-depictions. Despite the main effects described above, *situation* and *paradigm* interacted regarding both, hit rates and RTs. Not all differences in hit rates between paradigms were significant for all situations. Differences in RTs were non-significant for some situations, too. However, the same pattern revealed by the main effect of *scenario* (RTs for P1-bird<P2-help<P3-help-side) was present in all situations for RTs (see Figure 8).

In sum, both hit rates and RTs were profoundly and systematically influenced by task demands, indicating that the identical NeoHelp-stimuli can be employed in different tasks. Differences in response behavior between paradigms can thus be attributed to each task's specific demands without confounding effects of divergence in stimulus material.

### Effects of stimulus content on hit rates and RTs

#### S-1-bird: human-bird-distinction after short stimulus presentation

**Figure 9:**
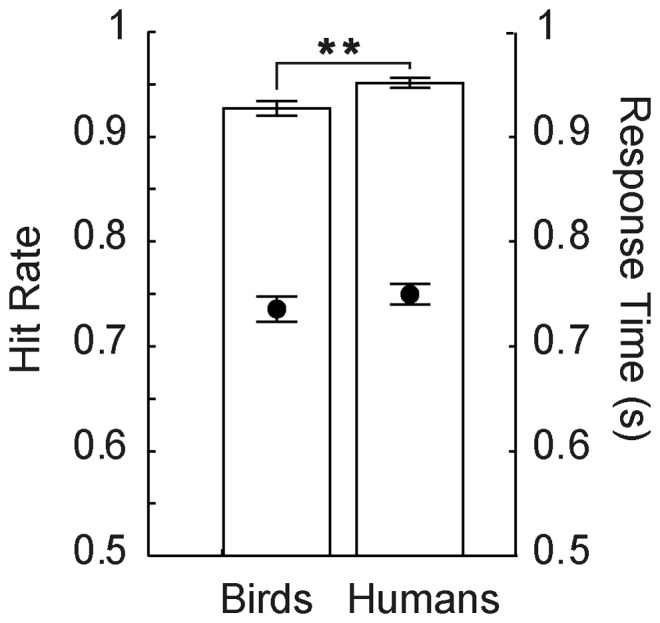
Hit rates and mean RTs for human- and bird-depictions in P1-bird. Bars represent hit rates, dots mean RTs. Asterisks mark significant differences in hit rates, **p<.01, as derived from post-hoc Tukey HSD tests. Error bars represent SEM. RTs did not differ between human and bird depictions.

The detailed results of the ANOVAs for both hit rates and RTs are given in Table 3. As response characteristics were not different across situations, or between no-NoH/NoH-depictions hit rates as well as mean RTs are shown for bird and human pictures on the left side of Figure 9. All interactions were non-significant. However, children's responses were more accurate for pictures of humans, MD = 2.35%, 95% CI[2.02%, 1.18%], p<.01. This effect was independent of whether need-of-help was depicted and of the single situation presented.

**Table 3:** Results of ANOVAs conducted in P1-bird.

		Hit Rates		RTs	
Source	df	F	p	F	p
Situation	11	1.12	.34	1.23	.26
NoH-depiction	1	0.27	.60	0.11	.74
Bird-depiction	1	8.29**	<.01	0.80	.37
Situation x NoH-depiction	11	1.14	.32	1.58	.10
Situation x bird-depiction	11	0.73	.71	0.83	.61
NoH-side x bird-depiction	1	3.09	.08	1.36	.24
Situation x NoH-depiction x bird-depiction	11	1.64	.08	0.95	.49

p<.01.

**Figure 10:**
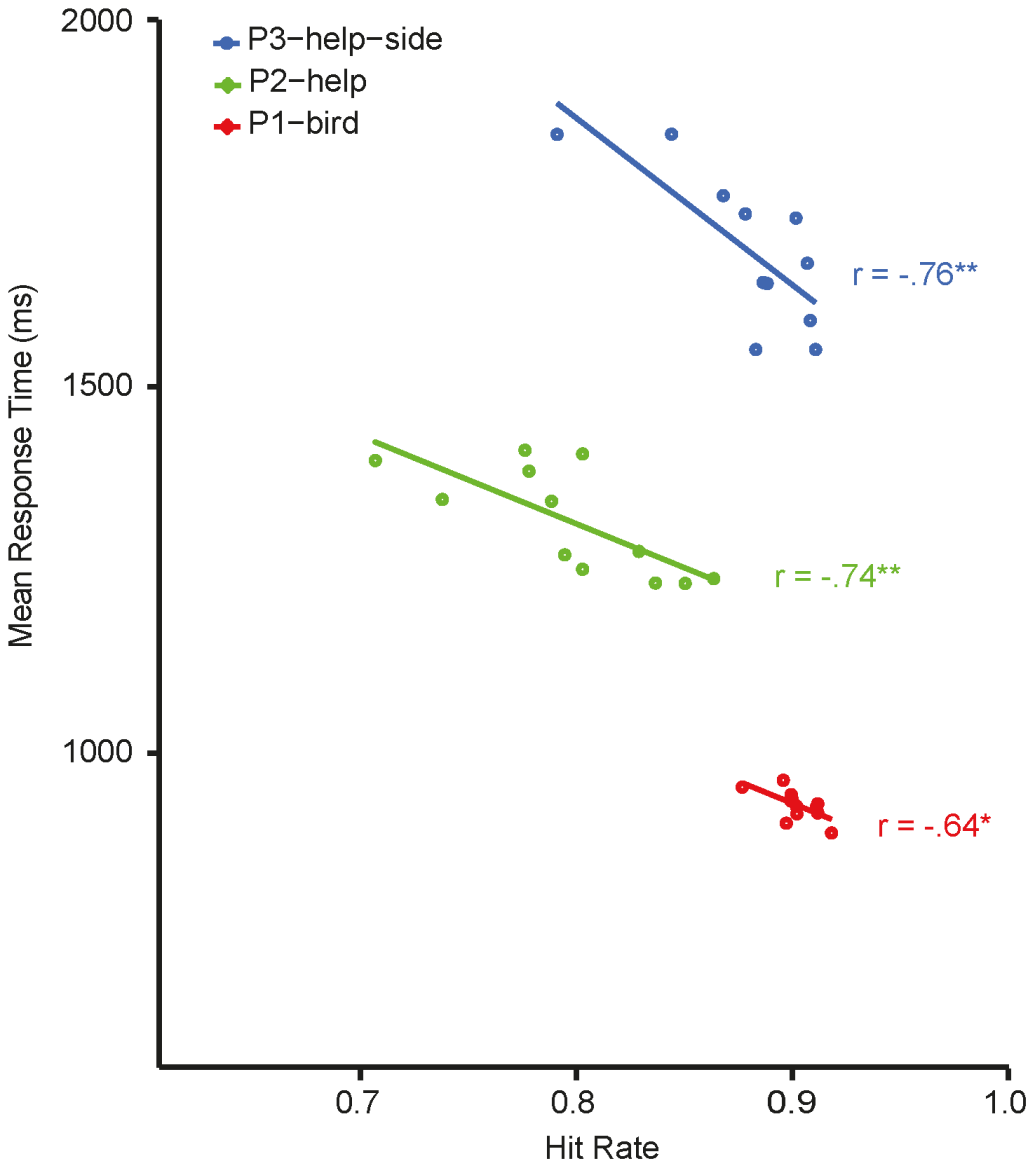
Relationship between hit rates and mean RTs. Each data point shows the mean RT for one situation as a function of its hit rate. Means and correlations were calculated separately for P3-help-side (blue), P2-help (green) and P1-bird (red). Asterisks mark significances according to Pearson's correlation tests: **p<.01, * p<.05. Regression lines were calculated by fitting linear models.

The relationship between hit rates and RTs for the different situations is illustrated in Figure 10 (red circles). A test of Pearson's correlation coefficient showed that there was no speed-accuracy-trade-off and revealed on the contrary that in general RTs were shorter for situations that were categorized more accurately, r = −.64, p = .02. RTs were not different because of the picture properties (*situation*, *bird-depiction* and *NoH-depiction*) or any combination of them.

There was a main effect of *correctness* on RTs, F(11, 3287) = 62.39, p<.001, with correct responses being faster than incorrect ones, MD = 252 ms, 95% CI[189 ms, 315 ms], p<.001. Even though RTs were not influenced by *situation* directly if correct and incorrect responses were distinguished, F(11, 3287) = 1.26, p = .24, there was a significant interaction of *correctness* with *situation*, F(11, 3287) = 2.59, p<.01. Post-hoc tests clarified that significant differences emerged between incorrect and correct responses in all but two situations: “climb” and “shelf”.

#### S-2-help: NoH-/no-NoH-distinction after short stimulus presentation

**Figure 11:**
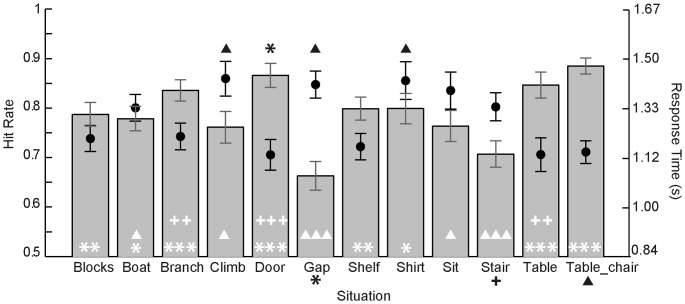
Response characteristics per situation in P2-help. Hit rates (bars) and mean RTs (dots) were calculated across all variations of a given situation. Symbols indicate significant differences in hit rates (white symbols) and RTs (black symbols). The number of symbols corresponds to p-values of post-hoc Tukey HSD tests (one: p<.05, two: p<.01, three: p<.001). Asterisks mark significant differences compared to the situation “gap”, triangles to “table_chair” and crosses to “stair”. Error bars represent SEM. Note that differences are only indicated once for each pair of situations.

The detailed results of the ANOVAs for both, hit rates and RTs, are given in Table 4. Figure 11 shows the mean hit rates and RTs for each situation. Hit rates in P2-help were profoundly influenced by the factors *situation, NoH-depiction* as well as *bird-depiction* and all two-way interactions. Note that this pattern clearly contrasts the results from P1-bird (same presentation mode, same stimuli, but different instruction: bird-human distinction required), where only *bird-depiction* influenced hit rates. Regarding differences between situations, post-hoc comparisons revealed that significant differences emerged between “gap”, “table_chair”, “stair” and the other situations (see white symbols Fig. 11). The results suggest that “gap” and “stair” can be considered as being more difficult to categorize in terms of NoH-distinction than other situations, even though the hit rates are still above chance. On the other hand, “table_chair” diverges from the other situations by means of being even more easily categorizable.

**Table 4:** Results of ANOVAs conducted in S-2-help.

		Hit Rates		RTs	
Source	df	F	p	F	p
Situation	11	7.60***	<.001	4.20***	<.001
NoH-depiction	1	88.15***	<.001	39.98***	<.001
Bird-depiction	1	23.06***	<.001	0.06	.81
Situation x NoH-depiction	11	3.36***	<.001	1.24	.26
Situation x bird-depiction	11	1.99*	.03	0.62	82
NoH-depiction x bird-depiction	1	75.84***	<.001	0.13	.72
Situation x NoH-depiction x bird-depiction	11	1.60	.09	0.56	.86

p<.001; *p<.05.

In general, NoH pictures were categorized better than no-NoH pictures, MD = 13.16%, 95% CI[15.90%, 10.41%], p<.001. Interestingly, pictures of birds were also identified more accurately as showing NoH or no-NoH, MD = 6.83%, 95% CI[3.99%, 9.66%], compared to pictures of humans. The interaction of the factors *bird*- and *NoH-depiction* showed that the difference between hit rates to bird and human depictions when the picture showed NoH was negligible, p = .09. The contingency table for this interaction is given in Table 5. In contrast, no-NoH pictures of birds were significantly better categorized than no-NoH-pictures of humans, p<.001. This also means that there were no differences in hit rates for pictures of humans depending on whether they showed someone in need for help or not, p = .07, while for pictures of birds this made a significant difference in the way that NoH depictions of birds were always correctly identified as such (see Table 5), whereas there was a considerable drop in response accuracy for no-NoH-depictions, p<.001.

**Table 5:** Mean hit rates and SDs illustrating the interaction of *bird-depiction* and *NoH-depiction* in the large-picture subgroup for P2-help.

		NoH-depiction	
		NoH	No-NoH
Bird-depiction	Human	.79 (.41)	.74 (.44)
	Bird	1.00 (.00)	.71 (.45)

NoH  =  need-of-help. Numbers in brackets represent SDs.

**Figure 12:**
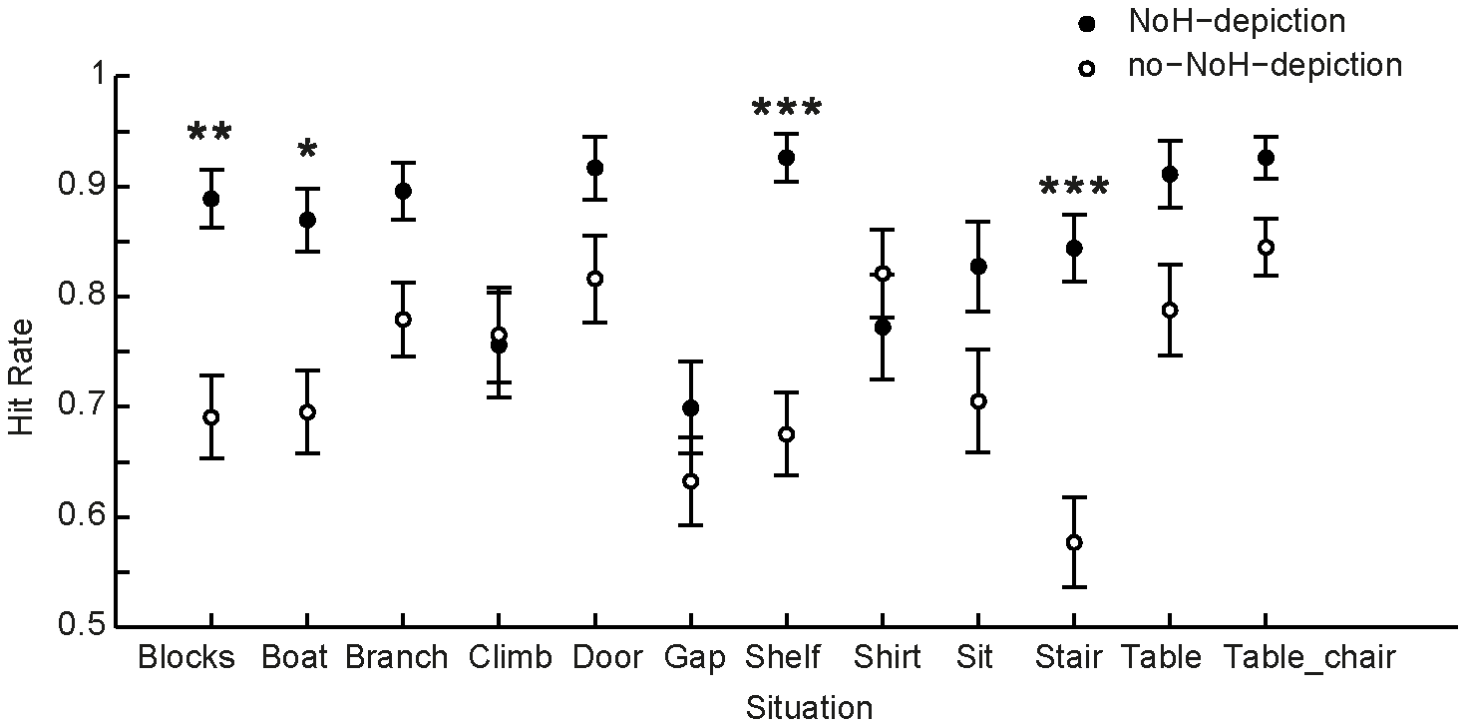
Interaction effect of NoH-depiction and situation on hit rates in P2-help. Filled black dots represent hit rates for NoH-depictions, hollow dots for no-NoH-depictions. Error bars represent SEM. Situations for which no-NoH/NoH-depictions were significantly different within the situation are marked with asterisks (*p<.05, ***p<.001, referring to post-hoc Tukey HSD tests). Also note that the main effect of NoH-depiction nonetheless shows that NoH-depictions are generally recognized better.

In addition to the main effect of situation described above, showing that the situations “gap” and “stair” had lower hit rates compared to other situations, whereas “table_chair” had higher ones, an interaction of *situation* and *NoH-depiction* emerged, as illustrated in Figure 12. The NoH-depiction elicited significantly higher hit rates for the situations “blocks”, “boat”, “shelf” and “stair”. For the other nine situations no significant post-hoc differences for no-NoH/NoH-depictions regarding hit rate were observed.

The correlation between RTs and hit rates was significant and again negative, r = −.74, p<.01, thus showing that there was no speed-accuracy-tradeoff (see green circles in Fig. 10), as demonstrated in earlier analyses, too. In contrast to hit rates, RTs were not affected by *bird-depiction*. On the other hand, main effects of *situation* as well as *NoH-depiction* were present for RTs, as well as for hit rates. In general, RTs were faster for NoH pictures, MD = 176 ms, 95% CI[121ms, 231 ms], p<.001. The four situations (“blocks”, “boat”, “shelf” and “stair”) for which post-hoc tests revealed significantly different RTs are marked with black symbols in Figure 12. All remaining main effects and interactions were non-significant.

In P2-help, we observed significantly faster RTs for correct compared to incorrect responses, F(11, 3031) = 14.69, p<. 001. Since a similar observation was made for P1-bird, the influence of *correctness* on response times did not seem to depend on the content of the task given, but rather on the way in which the stimulus was presented and the decision had to be made. On the other hand, RTs also depended critically on *situation* in this paradigm, F(11, 3031) = 4.19, p<.001. The interaction of the factors *situation* and *correctness* was non-significant, F(11, 3031) = 1.44, p = .15.

#### S-3-help-side: direct no-NoH/NoH comparison without time restriction

**Figure 13:**
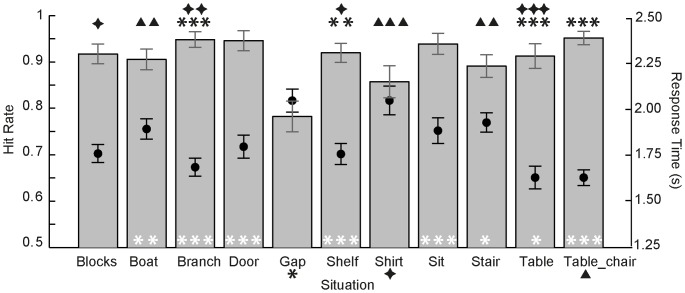
Response characteristics per situation in P3-help-side. Hit rates (bars) and mean RTs (dots) were calculated across all variations of a given situation. Symbols indicate significant differences in hit rates (white symbols) and RTs (black symbols). The number of symbols corresponds to p-values of post-hoc Tukey HSD tests (one: p<.05, two: p<.01, three: p<.001). Asterisks mark significant differences compared to the situation “gap”, triangles to “table_chair” and diamonds to “shirt”. Error bars represent SEM. Note that differences are only indicated once for each pair of situations.

The detailed results of the ANOVAs for both hit rates and RTs are given in Table 6. Mean hit rates and RTs for the different situations are presented in Figure 13. S*ituation* was one of two factors that had a main effect on hit rates. Post-hoc Tukey HSD tests showed that accuracy differences were only significant between the situation “gap” and most other situations (see white asterisks Fig. 13). This led to the assumption that the situation “gap” is systematically harder to differentiate than some easiest to differentiate situations, even though children were able to tell whether pictures of the situation “gap” depicted NoH or no-NoH above chance (see section “Ambiguous Situations”).

**Table 6:** Results of ANOVAs conducted in P3-help-side.

		Hit Rates		RTs	
Source	df	F	p	F	p
Situation	10	4.67***	<.001	6.93***	<.001
NoH-side	1	4.54*	.03	1.33	.25
Bird-depiction	1	0.85	.36	4.24*	.04
Situation x NoH-side	10	0.92	.52	1.20	.28
Situation x bird-depiction	10	0.78	.64	1.55	.12
NoH-side x bird-depiction	1	6.35*	.01	0.40	.53
Situation x NoH-side x bird-depiction	10	0.93	.51	1.18	.30

p<.001; *p<.05.

**Figure 14:**
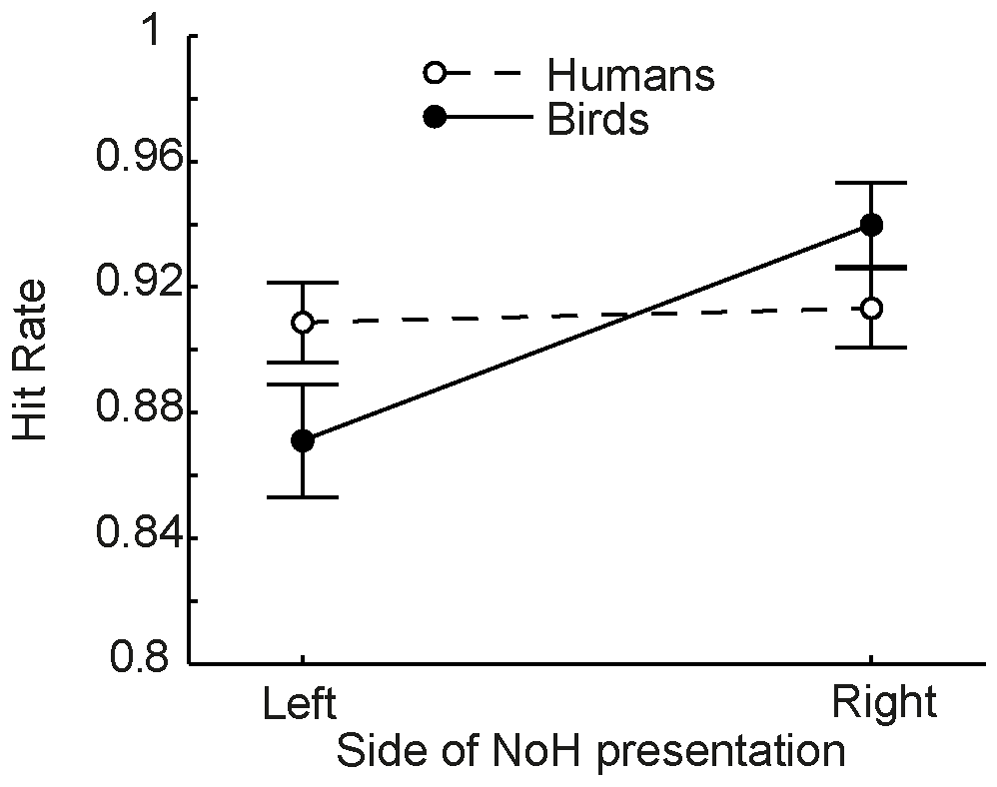
Interaction of side of need-of-help presentation (NoH-side) and bird-depiction in P3-help-side. Hit rates are shown across situations. Hollow circles connected with a dashed line represent data for human-depictions, filled circles connected with a solid line for bird-depictions. Error bars represent SEM. Note that the only significant difference in post-hoc tests is the one between NoH-depictions of birds shown on the left vs. right side, p<.01, all remaining p>. 07.

A main effect of *NoH-side* demonstrated that NoH-depictions were more accurately categorized as such, when shown on the right side of the screen. However, an interaction of *bird-depiction* and *NoH-side* put this finding into proportion: Post-hoc TukeyHSD revealed that significant differences in response accuracy for picture-pairs in which NoH-pictures shown on the left vs. right side emerged only for pictures of birds, not for those of humans (Fig. 14). This means, that for NoH-pictures of humans the position on the screen did not influence hit rates, while NoH in pictures of birds was better recognized when shown on the right side of the screen. All remaining interactions were non-significant.

The ANOVA conducted on RTs yielded a different pattern of results compared to the one for hit rates. *NoH-side* had no main effect on RTs. In contrast, not only *situation* but also *bird-depiction* influenced mean RTs, independently from each other: RTs were slower for pictures of birds, MD = 70 ms, 95% CI[2 ms, 137 ms], p = .04. Post-hoc comparison of RTs for different situations did not correspond to comparisons of hit rates for different situations: The mean RT for the “gap” situation was sometimes equal and sometimes slower compared to situations that were significantly better categorized than “gap” (compare presence of black and white asterisks in Fig. 13). Moreover, there were also significant differences in RTs between other situations that did not differ regarding hit rates, i.e. “table_chair” and “shirt” (see black triangles and diamonds, Fig. 13). These findings indicate that RTs are sensitive to some other aspect of help recognition than hit rates.

A test of Pearson's correlation coefficient again confirmed that there was no speed-accuracy-trade-off and revealed on the contrary that in general RTs were shorter for situations that were categorized more accurately, r = -.76, p<.01 (see blue circles Fig. 10).

*Correctness* did not influence RTs in this paradigm, in which there were no time constraints for stimulus presentation, F(10, 1654) = 0.03, p = .85, as opposed to the two time-constrained paradigms. By contrast, the main effect of *situation* was significant, F(10, 1654) = 6.88, p<.001 (see Fig. 13). The interaction of *situation* and *correctness* was non-significant, F(10, 1654) = 1.16, p = .31.

## Discussion

We have developed NeoHelp: an open access set of standardized visual stimuli for studying need-of-help recognition, a precondition for active helping. Our results provide evidence that a) all picture pairs comprising the NeoHelp are characterized by high similarity regarding perceptual properties, b) the pictures' content is correctly distinguished by children aged between four and 13 years for all but one situation, and c) different tasks can be applied using the NeoHelp stimulus set producing distinct and consistent response patterns. We provide standard response characteristics for the selection of stimuli and comparison across future studies. Thus the NeoHelp overcomes the limitations of previously used visual stimulus material for studies of social behavior and cognition.

Our study consists of three different tasks with varying demands. It is important to note that identical stimuli were across all tasks (human and bird depictions). The results demonstrate that both time restricted stimulus presentation and the concrete categorization task (human-bird, as well as need-of-help content) lead to characteristic changes in hit rates as well as RTs. Thus, we provide evidence that the NeoHelp stimuli elicit coherent and predictable changes in response characteristics, making them suitable for a wide range of experimental designs. Hit rates were lowest in the paradigm with the highest task load (P2-help: need-of-help distinction and time restricted stimulus presentation and decision from memory). Changing the task to an (easier) human vs. bird distinction while keeping the other experimental parameters constant (time restricted stimulus presentation, decision mode, etc.) shortened RTs and increased hit rates. Allowing open-ended pair-wise picture comparison similarly increased accuracy. It is noteworthy that for hit rates changing task content had a greater effect than prolonging stimulus presentation, whereas RTs depended more strongly on the mode of stimulus presentation and decision (see Fig. 7).

We demonstrate in our study that children are able to accomplish these three different tasks with identical stimulus material. The content of the NeoHelp stimuli was reliably recognized by children: Only one situation - “climb” - turned out to be ambiguous concerning need-of-help-content under certain conditions (see Fig. 6). None of the picture pairs were ambiguous with regard to the species depicted (here human children vs. birds). The NeoHelp includes stimuli depicting birds that are highly similar in terms of perception but easily distinguished. They are useful in control conditions for example as they tell apart general categorization abilities, which for example rely on speed of processing or cognitive capacity, from specific help-content-related effects. Children recognized whether a bird or a human was depicted highly reliably even when pictures were presented for only 500 ms and a decision was taken after stimulus offset (see Fig. 6 C). How the general ability to differentiate between humans and birds relates to the more specific need-of-help distinction is treated in other analyses by the authors (Stolarova & Brielmann, under review). In this report, we have opted to concentrate on the suitability of the newly created stimuli and their usefulness in three mutually controlling paradigms.

**Figure 15:**
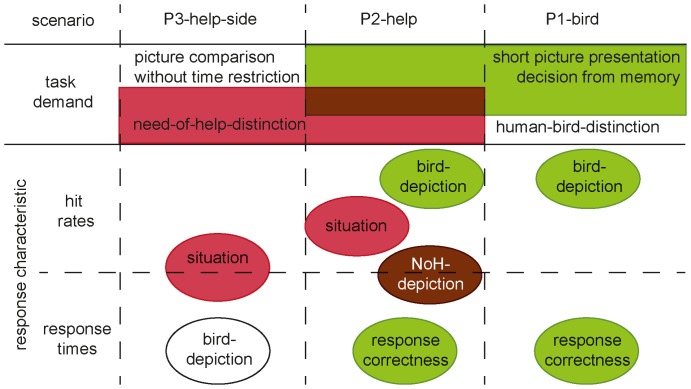
Overview of main effects on hit rates and mean RTs per paradigm. Factors that had a significant main effect are written inside ellipses. Red shaded ellipses indicate relationships with the task demand “need-for-help-distinction”, green shaded areas indicate relationships with the task demand “short picture presentation and decision from memory” and overlapping shading indicate that both task demands had an influence. White ellipses illustrate that the effect was strictly paradigm-specific. Ellipses located at the top refer to main effects on hit rates only, ellipses located at the bottom to main effects on RTs only. Ellipses that lie on the dashed line separating top and bottom panel show factors that exerted main effects on hit rates as well as RTs.

The three paradigms were created in a way that allowed us to disentangle the influences of NoH-distinction as task from those of the mode of stimulus presentation. Distinct task demands lead to discernible effect patterns regarding influences of picture properties, as illustrated in Figure 15. Most importantly, systematic differences of response characteristics for different situations emerged only when children were asked to decide whether or which picture showed someone in need of help, not when a human-bird distinction was required. Thus, we succeeded in creating situations that differ in their NoH-related clarity of content. At this point, however, we can only speculate as to which characteristics of a situation make it more or less easy for children to categorize. It is likely that a higher amount of experience with certain situations increased the ability of children to detect subtle changes illustrating the need-of-help [46], [47]. On the other hand, it is also possible that some aspect of the illustration of the situation might have influenced its clarity. To be able to draw definite conclusions about the reasons for differences between situations, the NeoHelp stimulus set needs to be extended and supplemented with ratings of familiarity and/or frequency of occurrence. Systematic analyses of population characteristics and their relationship with success of categorization will also provide significant insight. We hope that further studies using the NeoHelp stimulus set will help determine why it is easier for children to recognize need-for-help in certain situations and how this depends on the mode of presentation. What we do know from the present analyses is that the human-bird differentiation task provides a control for general categorization ability and processing and that by relating children's responses to the three mutually controlling paradigms, we will be able to differentiate between response characteristics specific to need-of-help recognition and general information processing.

Because of the controlled perceptual properties of the NeoHelp stimuli and in light of the above results, we suggest that they are well suited not only for behavioral testing but also for use in psychophysiological studies, such as EEG, eye tracking or fMRI. Also, since none of the tasks posed significant difficulties to the children in this study, we assume that the NeoHelp stimuli can also be employed in studies with adults, as well as with some clinical populations, too. It will thus be interesting to see how the NeoHelp stimuli will be used in future to illuminate a range of different research questions. Possible research areas of application are for example developmental changes and gender differences in need-of-help recognition and their relationship to actual helping behavior, the influence of different social experiences and other environmental factors on need-of-help recognition, its relationship with personality variables and clinical symptoms, as well as its underlying neurological and psychophysiological mechanisms. Our results show that need-of-help recognition differs across experimental designs with varying stimulus presentation durations and response modes. Influences of different picture contents emerge depending on the stimulus presentation mode used. It will therefore be interesting to e.g. find out why the depiction of a bird vs. a human might enhance or undermine NoH-recognition. Another important finding in our study was that the obtained effect patterns differed systematically for RTs and hit rates. This is consistent with literature documenting the fact that measures of accuracy and measures of speed are sensitive to different aspects of processing in adults [48], [49], as well as in children [50]. It remains for future studies, however, to illuminate which aspects of help-recognition are mirrored in hit rates and RTs.

The purpose of this article was to test and introduce the NeoHelp stimulus set as a research tool. We have addressed the relation between child-related factors such as age and gender, and need-of-help recognition in a separate analysis, so as not to overburden the present report. All researchers interested in using the NeoHelp stimulus set are highly encouraged to do so and should refer to Appendix S1 for detailed information about the pictures. The complete stimulus set is available for download as supplementary material Appendix S5, pictures are also available in formats which allow their modification into new variations. Every contribution to the enlargement and extension of the NeoHelp stimulus set is welcome.

## Supporting Information

Appendix S1 **Complete list of the pictures comprising the NeoHelp stimulus set.** All pictures of the stimulus set are depicted along with mean hit rates and RTs as well as SSIM values. An overview of hit rates above chance is provided, too.
(PDF)

Appendix S2 **Detailed description of the stimulus generation and the cover story.**
(PDF)

Appendix S3 **Analyses of data for a subgroup of children presented with smaller pictures.** The same analyses as presented in the main article were conducted in this subgroup. These analyses provide additional information for three situations of the NeoHelp stimulus set.
(PDF)

Appendix S4 **Analyses of picture variations and paradigm sequence.** Detailed statistics regarding the influence of variation in the human depictions and the sequence of paradigms P1-bird and P2-help are provided. Whereas no differences between human variations of one situation were observed, different paradigm sequences resulted in different hit rates and mean RTs.
(PDF)

Appendix S5 **All NeoHelp stimuli in JPEG format.** All stimuli are provided as used in the study reported.
(ZIP)
