# Supplementary material for: A New Standardized Stimulus Set for Studying Need-of-Help Recognition (NeoHelp)
Source: PLoS One. 2014 Jan 7;9(1):e84373. doi: 10.1371/journal.pone.0084373 (PMC3883661; doi:10.1371/journal.pone.0084373)
Supplement: Appendix S1 — Complete list of the pictures comprising the NeoHelp stimulus set. All pictures of the stimulus set are depicted along with mean hit rates and RTs as well as SSIM values. An overview of hit rates above chance is provided, too. (PDF) [file pone.0084373.s001.pdf]

**Appendix S1. Complete listing of picture and situation characteristics of the NeoHelp stimulus set.**

The data presented was obtained from N = 80 children (3 to 13 yrs., M = 8.19 yrs., SD = 2.26; 62.5% male). Hit rates and mean response times (RTs in ms) were calculated separately for different paradigms. Means were calculated across valid trials (n) for each situation and picture.

We will first present standards for response characteristics and clarity of content for the 15 different situations. The data for small (max. visual angle = 11.75°, N = 22) and large picture presentation (max. visual angle = 15.62°, N = 60) will be presented in separate tables. Three situations ("apple", "bucket" and "drawer") were only analyzed if presented in small size.

Second, response characteristics and picture properties will be presented for each individual picture. As a measure of low-level perceptual similarity between pictures of one situation as well within each picture pair, we have calculated the structural similarity relative to the reference picture for NoH-depictions and relative to the corresponding NoH-depiction for all no-NoH-depictions (see Wang, Brovik, Sheikh & Simoncelli, 2004, for a detailed description of the index). In order to increase n, picture size was not considered when calculating hit rates and mean RTs for individual pictures.

**Tables listing mean response characteristics across all picture variations.**

Situations are sorted by hit rate in ascending order.

1. Need-of-help-distinction task; pairwise picture presentation without time restriction

| Situation | n | large picture presentation: hit rates | | RTs | | hit rate above chance | pages |
|---|---|---|---|---|---|---|---|
| | | mean | SD | mean | SD | | |
| table_chair | 226 | 0,95 | 0,22 | 1626 | 634 | ✓ | 20-21 |
| branch | 173 | 0,95 | 0,22 | 1683 | 632 | ✓ | 9 |
| door | 111 | 0,95 | 0,23 | 1793 | 656 | ✓ | 12 |
| sit | 114 | 0,94 | 0,24 | 1880 | 743 | ✓ | 17 |
| shelf | 174 | 0,92 | 0,27 | 1754 | 746 | ✓ | 15 |
| blocks | 169 | 0,92 | 0,28 | 1756 | 633 | ✓ | 7 |
| table | 114 | 0,91 | 0,28 | 1627 | 651 | ✓ | 19 |
| boat | 169 | 0,91 | 0,29 | 1888 | 707 | ✓ | 8 |
| stair | 165 | 0,89 | 0,31 | 1923 | 678 | ✓ | 18 |
| shirt | 105 | 0,86 | 0,35 | 2042 | 793 | ✓ | 16 |
| gap | 156 | 0,78 | 0,41 | 2041 | 780 | ✓ | 14 |
| climb | 92 | 0,57 | 0,50 | 2072 | 881 | ✗ | 11 |

| Situation | n | small picture presentation: hit rates | | RTs | | hit rate above chance | pages |
|---|---|---|---|---|---|---|---|
| | | mean | SD | mean | SD | | |
| sit | 30 | 1,00 | 0,00 | 1600 | 704 | ✓ | 17 |
| shirt | 32 | 0,97 | 0,18 | 1989 | 915 | ✓ | 16 |
| table | 32 | 0,97 | 0,18 | 1620 | 660 | ✓ | 19 |
| branch | 49 | 0,96 | 0,20 | 1568 | 606 | ✓ | 9 |
| boat | 51 | 0,94 | 0,24 | 1650 | 686 | ✓ | 8 |
| drawer | 49 | 0,94 | 0,24 | 1585 | 693 | ✓ | 13 |
| door | 32 | 0,94 | 0,25 | 1536 | 456 | ✓ | 12 |
| shelf | 51 | 0,90 | 0,30 | 1614 | 845 | ✓ | 15 |
| table_chair | 67 | 0,88 | 0,33 | 1496 | 597 | ✓ | 20-21 |
| apple | 47 | 0,87 | 0,34 | 2024 | 778 | ✓ | 6 |
| stair | 45 | 0,87 | 0,34 | 1757 | 622 | ✓ | 18 |
| blocks | 49 | 0,82 | 0,39 | 1894 | 770 | ✓ | 7 |
| gap | 44 | 0,80 | 0,41 | 2088 | 857 | ✓ | 14 |
| bucket | 40 | 0,78 | 0,42 | 2050 | 1059 | ✓ | 10 |
| climb | 29 | 0,59 | 0,50 | 1775 | 993 | ✗ | 11 |

**Tables listing mean response characteristics across all picture variations** (continued)**.**

2. Need-of-help-distinction task; single picture presentation limited to 500 ms

| Situation | n | large picture presentation: hit rates mean | hit rates SD | RTs mean | RTs SD | hit rate above chance | pages |
|---|---|---|---|---|---|---|---|
| table_chair | 390 | 0,88 | 0,32 | 1185 | 754 | ✓ | 20-21 |
| door | 194 | 0,87 | 0,34 | 1176 | 713 | ✓ | 12 |
| table | 189 | 0,85 | 0,36 | 1176 | 778 | ✓ | 19 |
| branch | 298 | 0,84 | 0,37 | 1238 | 775 | ✓ | 9 |
| shirt | 174 | 0,80 | 0,40 | 1425 | 839 | ✓ | 16 |
| shelf | 303 | 0,80 | 0,40 | 1203 | 766 | ✓ | 15 |
| blocks | 296 | 0,79 | 0,41 | 1230 | 753 | ✓ | 7 |
| boat | 289 | 0,78 | 0,42 | 1334 | 764 | ✓ | 8 |
| sit | 182 | 0,76 | 0,43 | 1392 | 840 | ✓ | 17 |
| climb | 180 | 0,76 | 0,43 | 1432 | 780 | ✓ | 11 |
| stair | 290 | 0,71 | 0,46 | 1337 | 803 | ✓ | 18 |
| gap * | 270 | 0,66 | 0,47 | 1413 | 743 | ✓ | 14 |

| Situation | n | small picture presentation: hit rates mean | hit rates SD | RTs mean | RTs SD | hit rate above chance | pages |
|---|---|---|---|---|---|---|---|
| table_chair | 106 | 0,91 | 0,29 | 1083 | 764 | ✓ | 20-21 |
| table | 54 | 0,81 | 0,39 | 1048 | 698 | ✓ | 19 |
| branch | 87 | 0,78 | 0,42 | 1074 | 770 | ✓ | 9 |
| door | 63 | 0,78 | 0,42 | 1151 | 697 | ✓ | 12 |
| blocks | 87 | 0,77 | 0,42 | 1166 | 778 | ✓ | 7 |
| shelf | 83 | 0,76 | 0,43 | 1146 | 710 | ✓ | 15 |
| climb | 53 | 0,75 | 0,43 | 1303 | 858 | ✓ | 11 |
| sit | 56 | 0,75 | 0,44 | 1208 | 789 | ✓ | 17 |
| drawer | 85 | 0,74 | 0,44 | 1017 | 678 | ✓ | 13 |
| shirt | 57 | 0,74 | 0,44 | 1331 | 755 | ✓ | 16 |
| boat | 87 | 0,72 | 0,45 | 1174 | 750 | ✓ | 8 |
| apple | 84 | 0,71 | 0,45 | 1230 | 683 | ✓ | 6 |
| stair | 87 | 0,69 | 0,47 | 1171 | 721 | ✓ | 18 |
| bucket | 80 | 0,63 | 0,49 | 1296 | 958 | ✓ | 10 |
| gap * | 72 | 0,61 | 0,49 | 1189 | 798 | ✗ | 14 |

* The situation "gap" was shown to have hit rates below chance if presented in a smaller size (vis. angle below 11.75°). When presented in larger size, hit rate exceeded chance level but was still lowest (66%) compared to all other situations.

**Tables listing mean response characteristics across all picture variations** (continued).

3. Human-bird-distinction task; single picture presentation limited to 500 ms

| Situation | n | large picture presentation: hit rates mean | hit rates SD | RTs mean | RTs SD | hit rate above chance | pages |
|---|---|---|---|---|---|---|---|
| table_chair | 419 | 0,96 | 0,19 | 694 | 403 | ✓ | 20-21 |
| branch | 317 | 0,95 | 0,21 | 750 | 448 | ✓ | 9 |
| shelf | 317 | 0,95 | 0,21 | 733 | 439 | ✓ | 15 |
| boat | 313 | 0,95 | 0,21 | 745 | 436 | ✓ | 8 |
| shirt | 197 | 0,94 | 0,24 | 731 | 426 | ✓ | 16 |
| door | 196 | 0,94 | 0,24 | 746 | 424 | ✓ | 12 |
| sit | 203 | 0,94 | 0,25 | 760 | 486 | ✓ | 17 |
| blocks | 309 | 0,94 | 0,25 | 768 | 425 | ✓ | 7 |
| gap | 308 | 0,94 | 0,25 | 757 | 462 | ✓ | 14 |
| stair | 310 | 0,93 | 0,25 | 713 | 426 | ✓ | 18 |
| climb | 215 | 0,93 | 0,26 | 796 | 449 | ✓ | 11 |
| table | 207 | 0,90 | 0,30 | 782 | 437 | ✓ | 19 |

| Situation | n | small picture presentation: hit rates mean | hit rates SD | RTs mean | RTs SD | hit rate above chance | pages |
|---|---|---|---|---|---|---|---|
| sit | 62 | 0,90 | 0,30 | 803 | 560 | ✓ | 17 |
| apple | 91 | 0,90 | 0,30 | 738 | 496 | ✓ | 6 |
| shelf | 91 | 0,90 | 0,30 | 723 | 430 | ✓ | 15 |
| boat | 87 | 0,90 | 0,31 | 814 | 501 | ✓ | 8 |
| bucket | 95 | 0,88 | 0,32 | 765 | 503 | ✓ | 10 |
| shirt | 67 | 0,88 | 0,33 | 701 | 448 | ✓ | 16 |
| stair | 91 | 0,88 | 0,33 | 661 | 354 | ✓ | 18 |
| gap | 87 | 0,87 | 0,33 | 867 | 580 | ✓ | 14 |
| table | 55 | 0,87 | 0,34 | 642 | 378 | ✓ | 19 |
| climb | 61 | 0,87 | 0,34 | 904 | 596 | ✓ | 11 |
| table_chair | 119 | 0,87 | 0,34 | 781 | 507 | ✓ | 20-21 |
| blocks | 92 | 0,86 | 0,35 | 780 | 510 | ✓ | 7 |
| branch | 92 | 0,86 | 0,35 | 807 | 534 | ✓ | 9 |
| drawer | 91 | 0,82 | 0,38 | 761 | 488 | ✓ | 13 |
| door | 62 | 0,81 | 0,40 | 733 | 473 | ✓ | 12 |

**Picture similarity, hit rates and mean RTs for individual pictures of the NeoHelp Stimulus Set.**

All individual pictures will be listed in alphabetical order of the situations' names in the following structure:

---

## "Situation X"

First picture pair of situation X

Specification of picture properties [human/bird; further specification of variation in gender, age, etc.]

**NoH-distinction** [data for a time restricted need-of-help-distinction task]
n = data sets available for the picture considered
hit rate = proportion of correct responses (SD)
RT = mean response time in ms (SD)

**human-bird-distinction** [data for a time restricted human-bird-distinction task]
[...]

need-of-help-picture

**NoH-distinction**
n =
hit rate = . (.)
RT = ()

**human-bird-distinction**
n =
hit rate = . (.)
RT = ()

no-need-of-help-picture

**NoH-distinction**
n =
hit rate = . (.)
RT = ()

**human-bird-distinction**
n =
hit rate = . (.)
RT = ()

**NoH-distinction** (pairwise comparison)
n =
hit rate = . (.)
RT = ()

**SSIM**
reference picture = 0.
NoH-depiction = 0.

**NoH-distinction** (pairwise comparison)
[data for a need-of-help-distinction task with pairwise picture comparison and unlimited picture presentation]
[...]

**SSIM** [the index used for picure similarity]
reference picture = SSIM comparing the NoH picture of the given variation and the NoH picture of the reference pair
NoH-depiction = SSIM comparing NoH and no-NoH pictures of the given variation

variation of situation X

new variation of situation X, not empirically tested yet

---

new page

## "Situation Y"

**“apple”** (data for small picture presentation only)

human; school age; boy

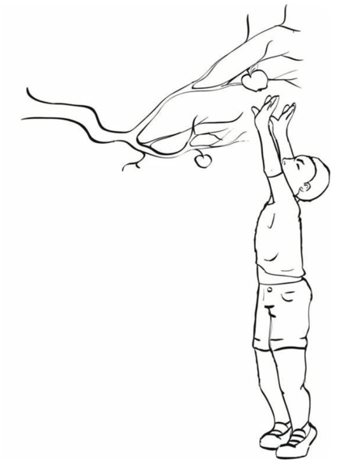

**NoH-distinction**
n = 12
hit rate = .92 (.29)
RT = 940 (401)

**human-bird-distinction**
n = 17
hit rate = .88 (.33)
RT = 772 (547)

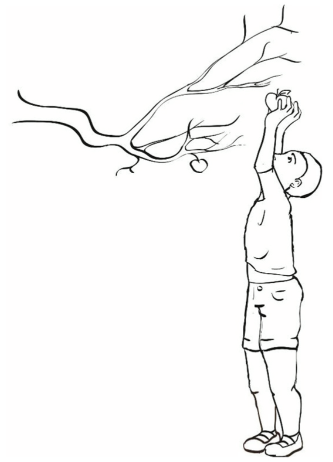

**NoH-distinction**
n = 15
hit rate = .40 (.51)
RT = 1382 (871)

**human-bird-distinction**
n = 13
hit rate = .92 (.28)
RT = 830 (490)

**NoH-distinction** (pairwise comparison)
n = 16
hit rate = .75 (.45)
RT = 1776 (582)

**SSIM**
reference picture = 0.83
NoH-depiction = 0.80

human; school age; girl

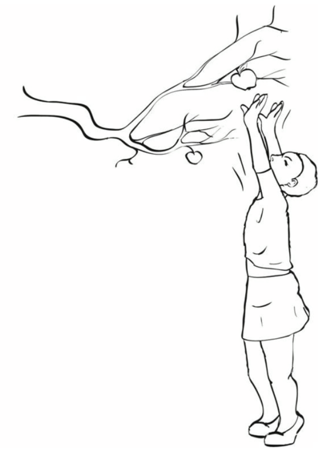

**NoH-distinction**
n = 15
hit rate = 1.00 (0.00)
RT = 661 (471)

**human-bird-distinction**
n = 13
hit rate = .92 (.28)
RT = 661 (471)

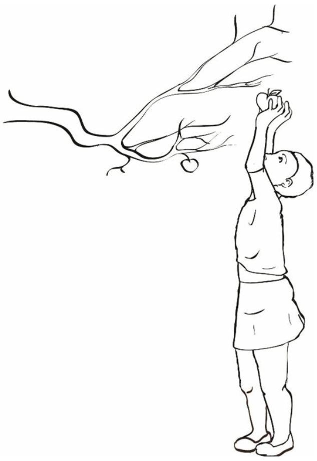

**NoH-distinction**
n = 13
hit rate = .38 (.51)
RT = 1497 (659)

**human-bird-distinction**
n = 17
hit rate = .88 (.33)
RT = 726 (451)

**NoH-distinction** (pairwise comparison)
n = 16
hit rate = .94 (.25)
RT = 2249 (895)

**SSIM**
reference picture = 1.00
NoH-depiction = 0.88

bird

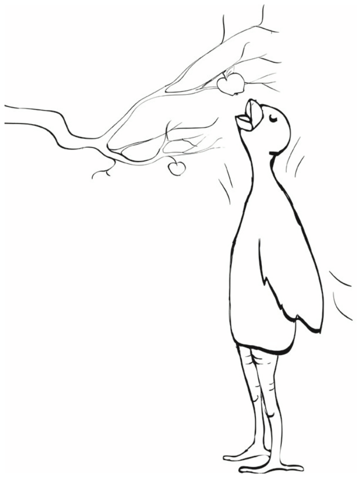

**NoH-distinction**
n = 13
hit rate = 1.00 (0.00)
RT = 1036 (619)

human-bird-distinction
n = 15
hit rate = .93 (.26)
RT = 737 (499)

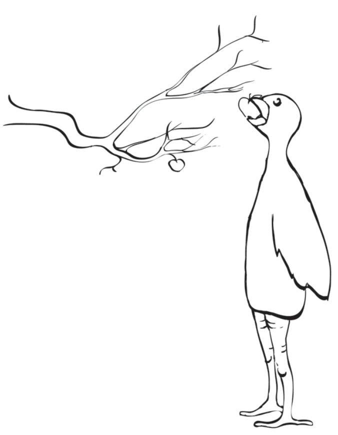

**NoH-distinction**
n = 16
hit rate = .63 (.50)
RT = 1447 (773)

**human-bird-distinction**
n = 16
hit rate = .88 (.34)
RT = 704 (568)

**NoH-distinction** (pairwise comparison)
n = 15
hit rate = .93 (.26)
RT = 2048 (631)

**SSIM**
reference picture = 0.89
NoH-depiction = 0.91

## “blocks”

human; kindergaden age; boy

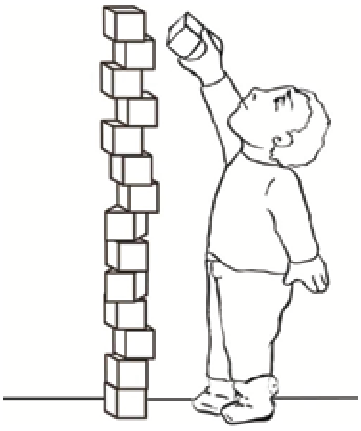

**NoH-distinction**
n = 67
hit rate = .76 (.43)
RT =  1226 (721)

**human-bird-distinction**
n = 70
hit rate = .96 (.20)
RT = 786 (445)

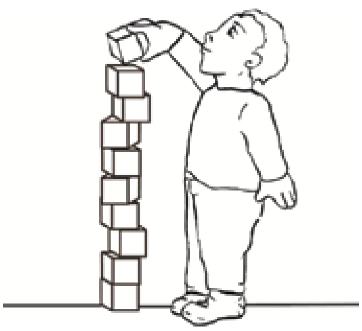

**NoH-distinction**
n = 65
hit rate = .83 (.38)
RT = 1179 (823)

**human-bird-distinction**
n = 67
hit rate = .88 (.33)
RT = 796 (466)

**NoH-distinction** (pairwise comparison)
n = 74
hit rate = . 88 (.33)
RT = 2248 (895)

**SSIM**
reference picture = 1.00
NoH-depiction =  0.81

---

human; kindergarden age; girl

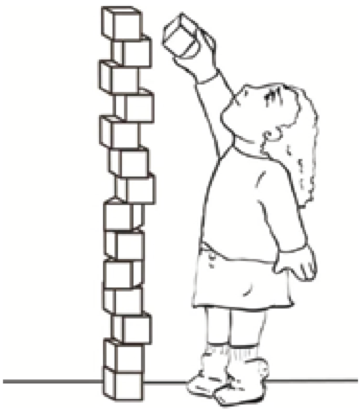

**NoH-distinction**
n = 63
hit rate = .86 (.35)
RT = 1175 (648)

**human-bird-distinction**
n = 67
hit rate = .93 (.26)
RT = 816 (453)

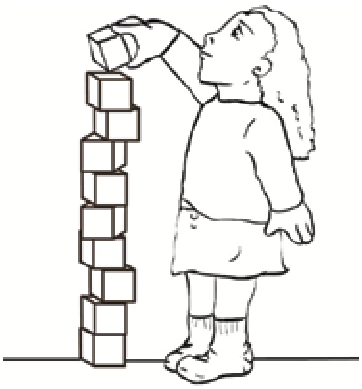

**NoH-distinction**
n = 68
hit rate = .71 (.46)
RT = 1132 (707)

**human-bird-distinction**
n = 65
hit rate = .89 (.31)
RT = 658 (369)

**NoH-distinction** (pairwise comparison)
n = 72
hit rate = .90 (.30)
RT = 1784 (582)

**SSIM**
reference picture = 0.81
NoH-depiction =   0.81

---

bird

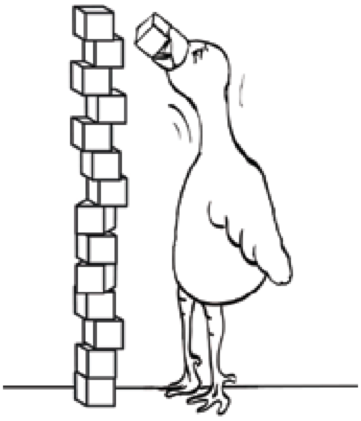

**NoH-distinction**
n = 59
hit rate = 1.00 (0.00)
RT =  1234 (770)

human-bird-distinction
n = 67
hit rate = . 88(.33)
RT =  801 (429)

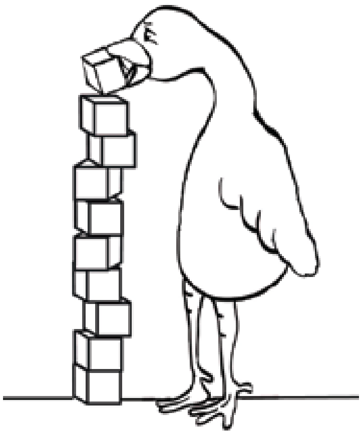

**NoH-distinction**
n = 61
hit rate = .56 (.50)
RT =  1362 (875)

**human-bird-distinction**
n = 65
hit rate = .97 (.17)
RT =  766 (498)

**NoH-distinction** (pairwise comparison)
n = 72
hit rate = .90 (.30)
RT =  1842 (631)

**SSIM**
reference picture = 0.82
NoH-depiction =  0.86

**"boat"**

human; kindergaden age; boy

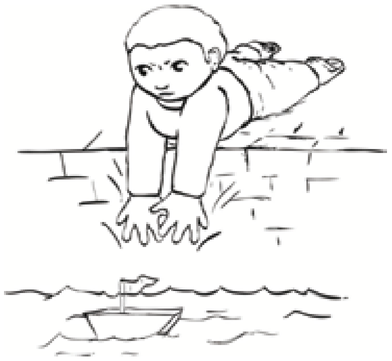

**NoH-distinction**
n = 64
hit rate = .81 (.39)
RT = 1303 (703)

**human-bird-distinction**
n = 67
hit rate = .93 (.26)
RT = 794 (484)

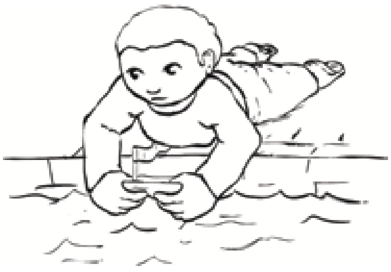

**NoH-distinction**
n = 64
hit rate = .64 (.48)
RT = 1272 (827)

**human-bird-distinction**
n = 67
hit rate = .96 (.21)
RT = 776 (456)

**NoH-distinction** (pairwise comparison)
n = 75
hit rate = . 91 (.29)
RT = 1817 (727)

**SSIM**
reference picture = 1.00
NoH-depiction = 0.76

human; kindergarden age; girl

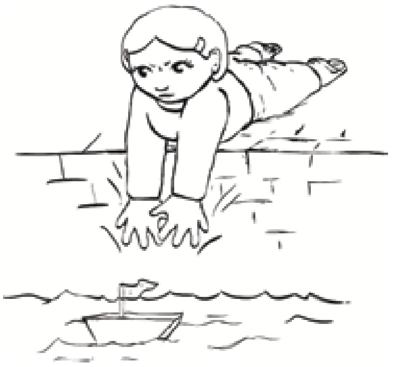

**NoH-distinction**
n = 64
hit rate = .80 (.41)
RT = 1240 (772)

**human-bird-distinction**
n = 69
hit rate = .94 (.24)
RT = 741 (478)

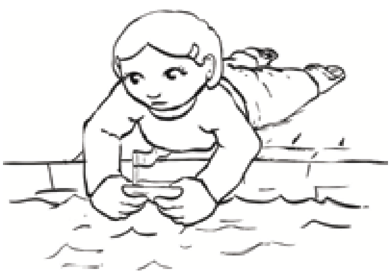

**NoH-distinction**
n = 63
hit rate = .75 (.44)
RT = 1375 (780)

**human-bird-distinction**
n = 62
hit rate = .90 (.30)
RT = 761 (465)

**NoH-distinction** (pairwise comparison)
n = 73
hit rate = . 92(.30)
RT = 1674 (598)

**SSIM**
reference picture = 0.85
NoH-depiction = 0.74

bird

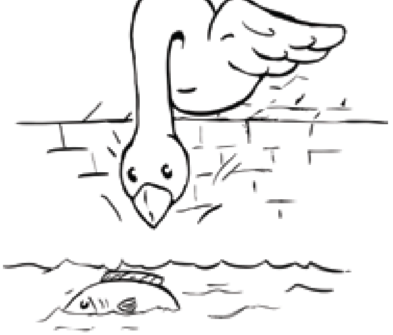

**NoH-distinction**
n = 53
hit rate = 1.00 (0.00)
RT = 1248 (794)

**human-bird-distinction**
n = 72
hit rate = .94 (.23)
RT = 775 (449)

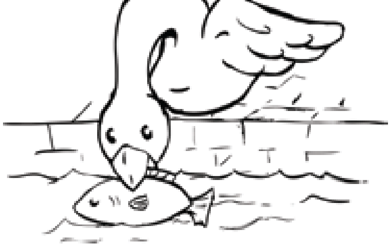

**NoH-distinction**
n = 68
hit rate = .65 (.48)
RT = 1334 (724)

**human-bird-distinction**
n = 63
hit rate = .97 (.18)
RT = 709 (377)

**NoH-distinction** (pairwise comparison)
n = 72
hit rate = . 92 (.28)
RT = 1842 (756)

**SSIM**
reference picture = 0.86
NoH-depiction = 0.89

## “branch”

human; school age; boy

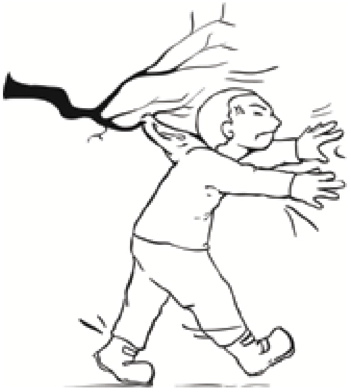

**NoH-distinction**
n = 67
hit rate = .82 (.39)
RT = 1086 (749)

**human-bird-distinction**
n = 68
hit rate = .93 (.26)
RT = 797 (481)

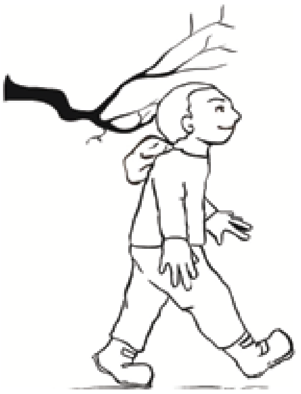

**NoH-distinction**
n = 64
hit rate = .77 (.43)
RT = 1384 (871)

**human-bird-distinction**
n = 70
hit rate = .91 (.28)
RT = 785 (491)

**NoH-distinction** (pairwise comparison)
n = 72
hit rate = .97 (.17)
RT = 1530 (582)

**SSIM**
reference picture = 1.00
NoH-depiction = 0.87

human; school age; girl

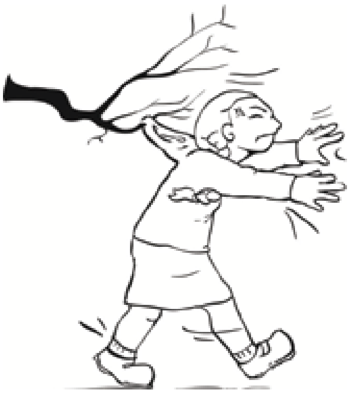

**NoH-distinction**
n = 63
hit rate = .86 (.35)
RT = 1078 (644)

**human-bird-distinction**
n = 69
hit rate = .96 (.21)
RT = 790 (514)

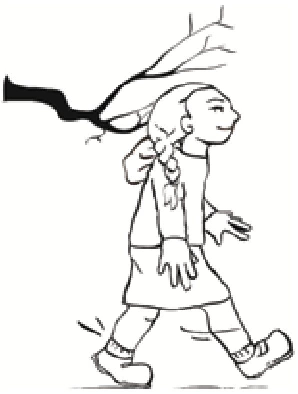

**NoH-distinction**
n = 63
hit rate = .76 (.43)
RT = 1255 (773)

**human-bird-distinction**
n = 70
hit rate = .94 (.23)
RT = 790 (514)

**NoH-distinction** (pairwise comparison)
n = 79
hit rate = .96 (.19)
RT = 1716 (638)

**SSIM**
reference picture = 0.91
NoH-depiction = 0.87

bird

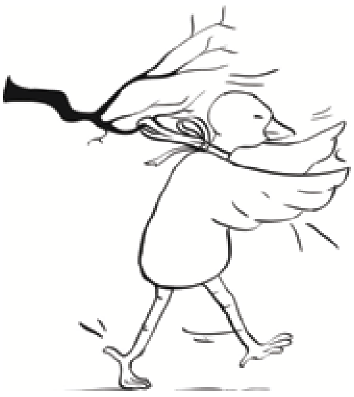

**NoH-distinction**
n = 59
hit rate = 1.00 (0.00)
RT = 910 (527)

human-bird-distinction
n = 70
hit rate = .90 (.30)
RT = 796 (480)

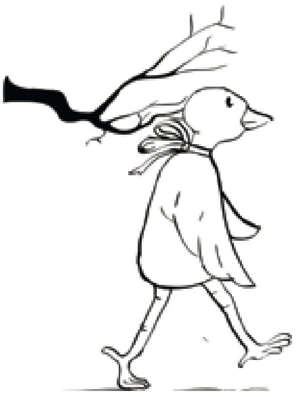

**NoH-distinction**
n = 69
hit rate = .75 (.43)
RT = 1452 (892)

**human-bird-distinction**
n = 62
hit rate = .95 (.22)
RT = 687 (392)

**NoH-distinction** (pairwise comparison)
n = 71
hit rate = .92 (.28)
RT = 1721 (648)

**SSIM**
reference picture = 0.89
NoH-depiction = 0.90

**"bucket"** (data for small picture presentation only)

human; school age; boy

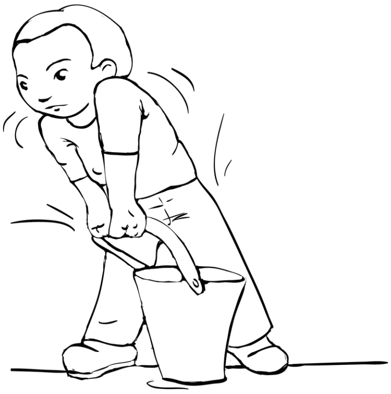

**NoH-distinction**
n = 14
hit rate = .71 (.47)
RT = 1092 (834)

**human-bird-distinction**
n = 13
hit rate = .92 (.28)
RT = 1025 (766)

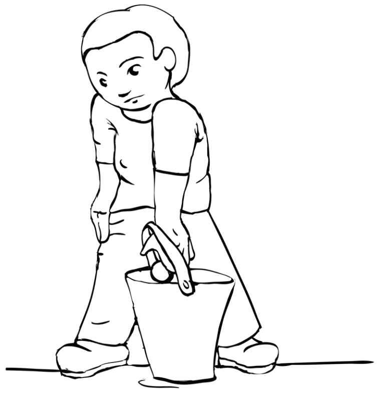

**NoH-distinction**
n = 14
hit rate = .50 (.52)
RT = 1475 (253)

**human-bird-distinction**
n = 15
hit rate = .87 (.35)
RT = 682 (473)

**NoH-distinction** (pairwise comparison)
n = 14
hit rate = . 71 (.47)
RT = 1880 (1022)

**SSIM**
reference picture = 1.00
NoH-depiction = 0.90

human; school age; girl

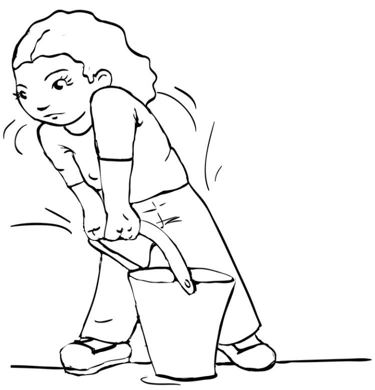

**NoH-distinction**
n = 15
hit rate = .73 (.46)
RT = 1338 (931)

**human-bird-distinction**
n = 15
hit rate = .80 (.41)
RT = 816 (546)

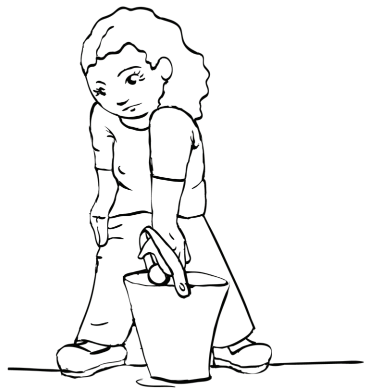

**NoH-distinction**
n = 13
hit rate = .46 (.52)
RT = 1186 (659)

**human-bird-distinction**
n =
hit rate = . (.)
RT = ()

**NoH-distinction** (pairwise comparison)
n = 12
hit rate = .83 (.39)
RT = 1948 (1121)

**SSIM**
reference picture = 0.90
NoH-depiction = 0.89

bird

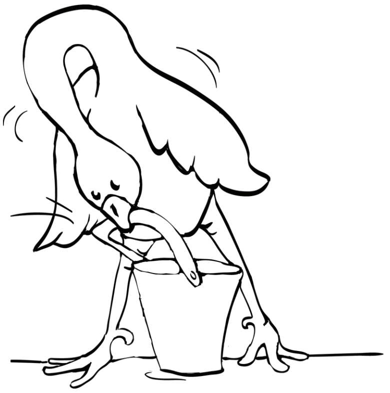

**NoH-distinction**
n =
hit rate = . (.)
RT = ()

human-bird-distinction
n =
hit rate = . (.)
RT = ()

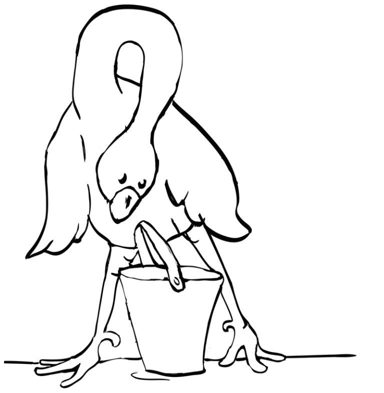

**NoH-distinction**
n =
hit rate = . (.)
RT = ()

**human-bird-distinction**
n =
hit rate = . (.)
RT = ()

**NoH-distinction** (pairwise comparison)
n = 14
hit rate = .79 (.43)
RT = 2308 (1071)

**SSIM**
reference picture = 0.88
NoH-depiction = 0.89

**"climb"**

human; toddler

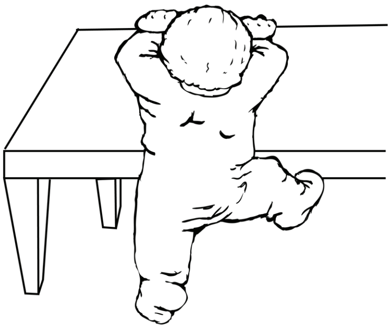

**NoH-distinction**
n = 65
hit rate = .65 (.48)
RT = 1369 (750)

**human-bird-distinction**
n = 71
hit rate = .93 (.26)
RT = 885 (442)

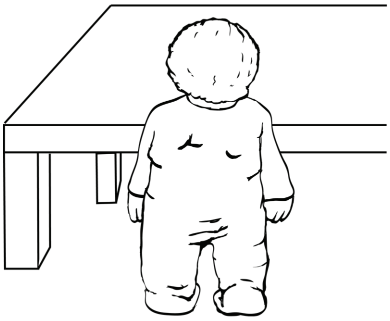

**NoH-distinction**
n = 66
hit rate = .85 (.36)
RT = 1409 (798)

**human-bird-distinction**
n = 67
hit rate = .93 (.26)
RT = 855 (502)

**NoH-distinction** (pairwise comparison)
n = 60
hit rate = .52 (.33)
RT = 2106 (958)

**SSIM**
reference picture = 1.00
NoH-depiction = 0.91

bird

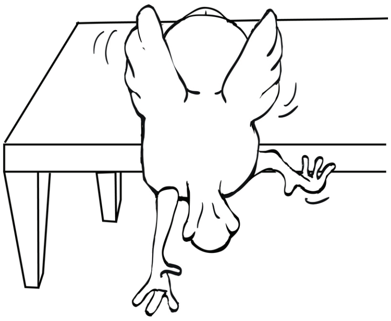

**NoH-distinction**
n = 42
hit rate = 1.00 (0.00)
RT = 1374 (859)

human-bird-distinction
n = 70
hit rate = .89 (.32)
RT = 797 (532)

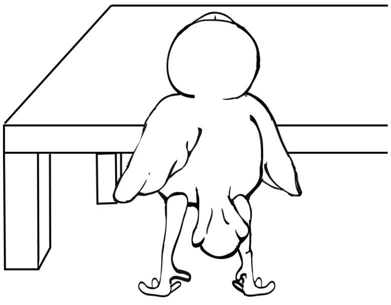

**NoH-distinction**
n = 60
hit rate = .62 (.49)
RT = 1454 (855)

**human-bird-distinction**
n = 68
hit rate = .93 (.26)
RT = 741 (462)

**NoH-distinction** (pairwise comparison)
n = 61
hit rate = .62 (.49)
RT = 1897 (862)

**SSIM**
reference picture = 0.84
NoH-depiction = 0.88

## “door”

human; school age; boy

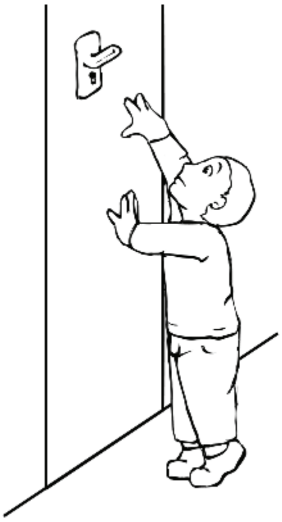

**NoH-distinction**
n = 69
hit rate = .87 (.34)
RT = 1013 (694)

**human-bird-distinction**
n = 62
hit rate = .95 (.22)
RT = 732 (358)

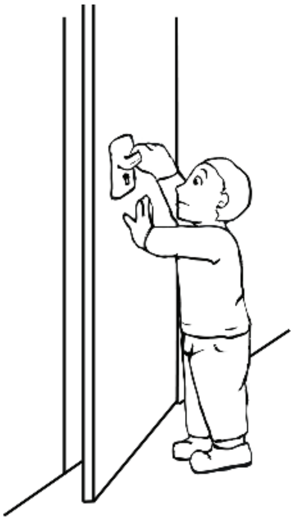

**NoH-distinction**
n = 63
hit rate = .73 (.45)
RT = 1291 (652)

**human-bird-distinction**
n = 62
hit rate = .95 (.22)
RT = 798 (441)

**NoH-distinction** (pairwise comparison)
n = 72
hit rate = .96 (.20)
RT = 1777 (672)

**SSIM**
reference picture = 1.00
NoH-depiction = 0.89

bird

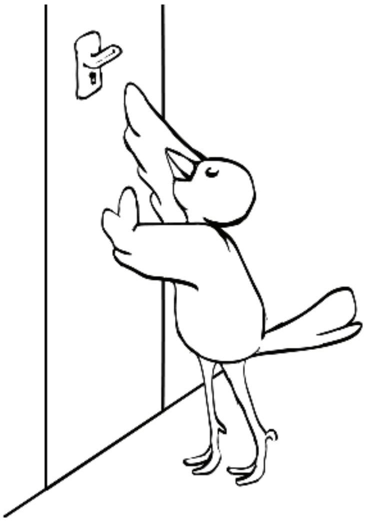

**NoH-distinction**
n = 59
hit rate = 1.00 (0.00)
RT = 1142 (773)

human-bird-distinction
n = 64
hit rate = .88 (.33)
RT = 667 (406)

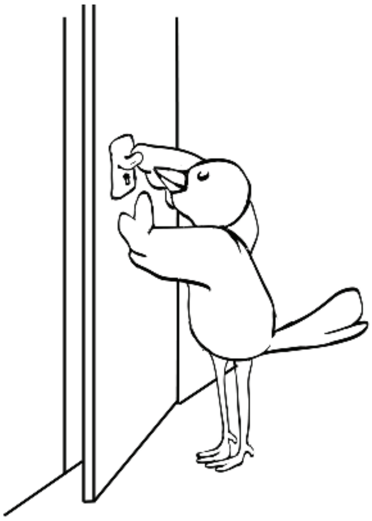

**NoH-distinction**
n = 66
hit rate = .79 (.41)
RT = 1242 (697)

**human-bird-distinction**
n = 70
hit rate = .86 (.35)
RT = 782 (511)

**NoH-distinction** (pairwise comparison)
n = 71
hit rate = .93 (.26)
RT = 1693 (574)

**SSIM**
reference picture = 0.90
NoH-depiction = 0.83

**“drawer”** (data for small picture presentation only)

human; school age; boy

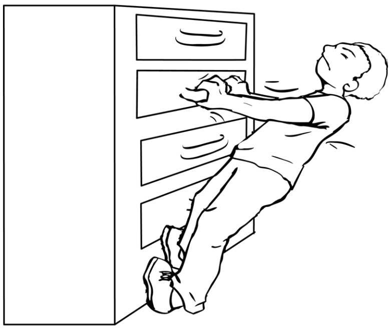

**NoH-distinction**
n = 13
hit rate = 1.00 (0.00)
RT = 739 (456)

**human-bird-distinction**
n = 13
hit rate = .69 (.48)
RT = 696 (411)

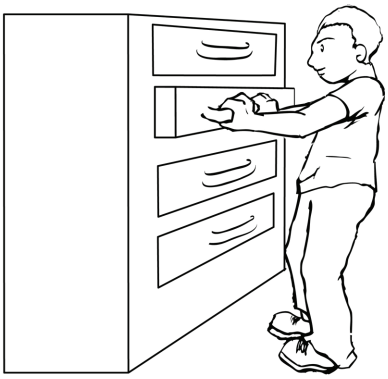

**NoH-distinction**
n = 15
hit rate = .40 (.51)
RT = 1129 (811)

**human-bird-distinction**
n = 13
hit rate = .85 (.38)
RT = 684 (413)

**NoH-distinction** (pairwise comparison)
n = 17
hit rate = . 88 (.33)
RT = 1684 (789)

**SSIM**
reference picture = 1.00
NoH-depiction = 0.86

human; school age; girl

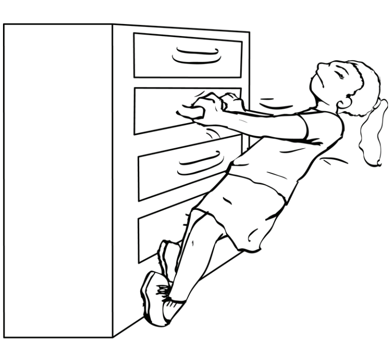

**NoH-distinction**
n = 15
hit rate = .87 (.35)
RT = 893 (499)

**human-bird-distinction**
n = 15
hit rate = .67 (.49)
RT = 926 (661)

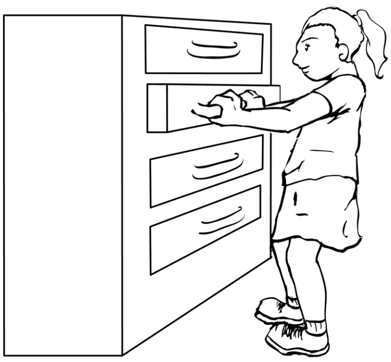

**NoH-distinction**
n = 17
hit rate = .47 (.51)
RT = 1102 (705)

**human-bird-distinction**
n = 18
hit rate = .89 (.32)
RT = 709 (314)

**NoH-distinction** (pairwise comparison)
n = 16
hit rate = 1.00 (0.00)
RT = 1512 (632)

**SSIM**
reference picture = 0.90
NoH-depiction = 0.86

bird

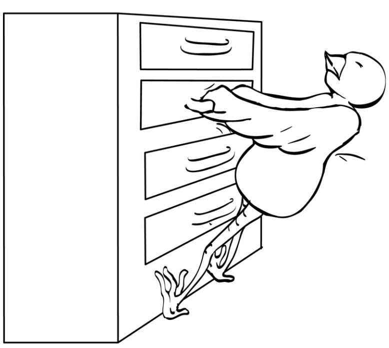

**NoH-distinction**
n = 12
hit rate = 1.00 (0.00)
RT = 841 (686)

human-bird-distinction
n = 14
hit rate = .93 (.27)
RT = 722 (522)

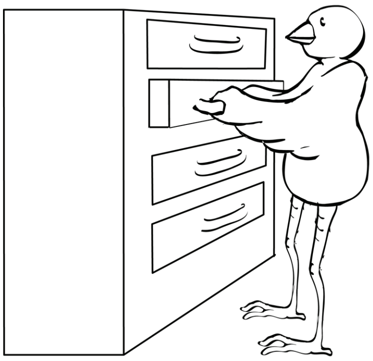

**NoH-distinction**
n = 13
hit rate = .85 (.38)
RT = 1357 (754)

**human-bird-distinction**
n = 18
hit rate = .89 (.32)
RT = 809 (564)

**NoH-distinction** (pairwise comparison)
n = 16
hit rate = .94 (.25)
RT = 1552 (675)

**SSIM**
reference picture = 0.89
NoH-depiction = 0.88

**“gap”**

human; school age; boy

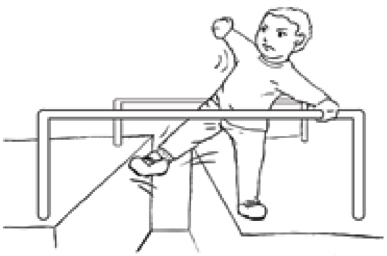

**NoH-distinction**
n = 59
hit rate = .59 (.50)
RT = 1201 (732)

**human-bird-distinction**
n = 61
hit rate = .93 (.25)
RT = 798 (524)

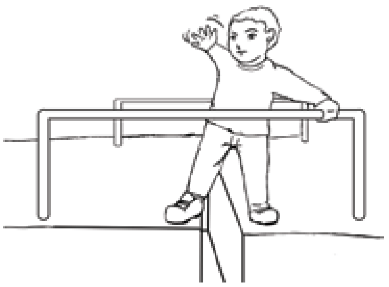

**NoH-distinction**
n = 67
hit rate = .60 (.49)
RT = 1404 (849)

**human-bird-distinction**
n = 62
hit rate = .94 (.25)
RT = 758 (483)

**NoH-distinction** (pairwise comparison)
n = 67
hit rate = .78 (.42)
RT = 1957 (781)

**SSIM**
reference picture = 1.00
NoH-depiction = 0.92

human; school age; girl

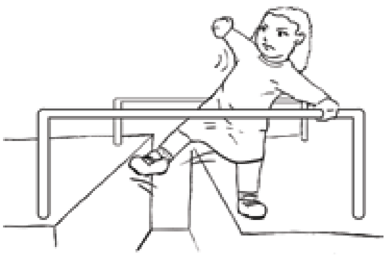

**NoH-distinction**
n = 64
hit rate = .66 (.48)
RT = 1360 (608)

**human-bird-distinction**
n = 65
hit rate = .88 (.33)
RT = 831 (495)

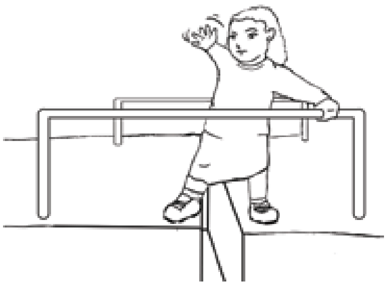

**NoH-distinction**
n = 65
hit rate = .63 (.49)
RT = 1529 (896)

**human-bird-distinction**
n = 69
hit rate = .96 (.96)
RT = 735 (502)

**NoH-distinction** (pairwise comparison)
n = 70
hit rate = .74 (.44)
RT = 2107 (847)

**SSIM**
reference picture = 0.99
NoH-depiction = 0.93

bird

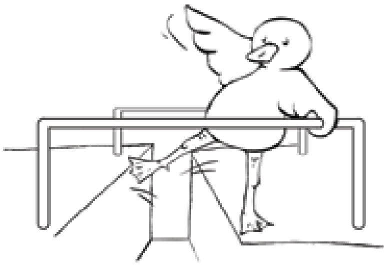

**NoH-distinction**
n = 30
hit rate = 1.00 (0.00)
RT = 1173 (554)

human-bird-distinction
n = 71
hit rate = .92 (.28)
RT = 813 (458)

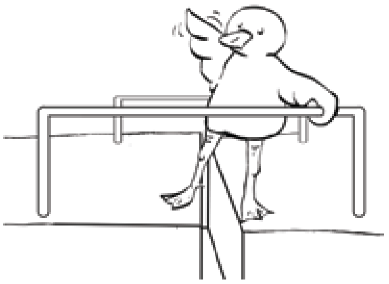

**NoH-distinction**
n = 57
hit rate = .61 (.49)
RT = 1412 (727)

**human-bird-distinction**
n = 67
hit rate = .91 (.29)
RT = 752 (500)

**NoH-distinction** (pairwise comparison)
n = 63
hit rate = .84 (.37)
RT = 2091 (756)

**SSIM**
reference picture = 0.94
NoH-depiction = 0.94

## “shelf”

human; toddler; Caucasian

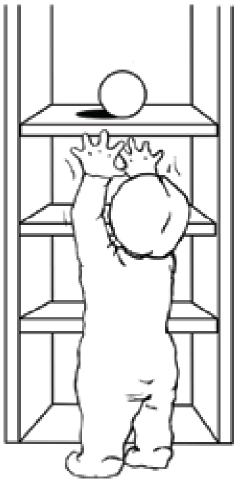

**NoH-distinction**
n = 67
hit rate = .85 (.36)
RT = 1138 (741)

**human-bird-distinction**
n = 64
hit rate = .98 (.13)
RT = 725 (436)

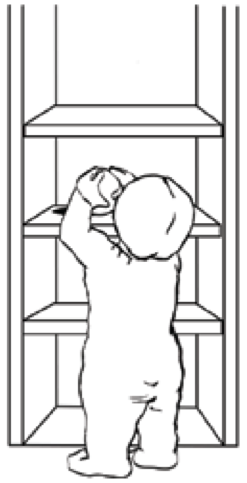

**NoH-distinction**
n = 65
hit rate = .80 (.40)
RT = 1289 (781)

**human-bird-distinction**
n = 67
hit rate = .87 (.34)
RT = 811 (445)

**NoH-distinction** (pairwise comparison)
n =
hit rate = .93 (.25)
RT = 1616 (701)

**SSIM**
reference picture = 1.00
NoH-depiction = 0.82

human; toddler; African Native

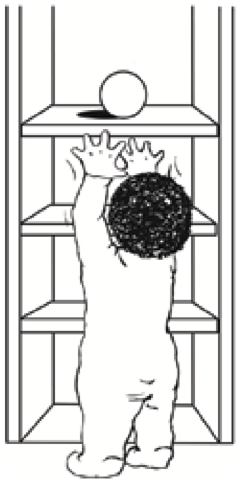

**NoH-distinction**
n =
hit rate = . (.)
RT = ()

**human-bird-distinction**
n =
hit rate = . (.)
RT = ()

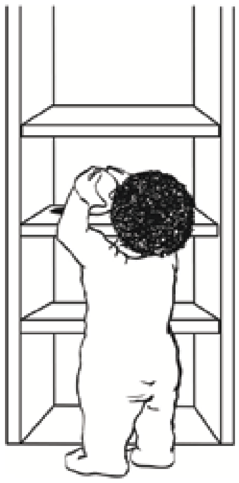

**NoH-distinction**
n =
hit rate = . (.)
RT = ()

**human-bird-distinction**
n =
hit rate = . (.)
RT = ()

**NoH-distinction** (pairwise comparison)
n =
hit rate = .93 (.25)
RT = 1658 (739)

**SSIM**
reference picture = 0.65
NoH-depiction = 0.73

bird

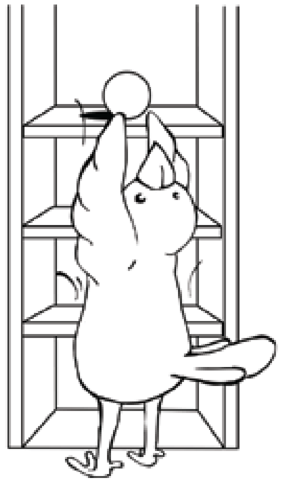

**NoH-distinction**
n =
hit rate = . (.)
RT = ()

human-bird-distinction
n =
hit rate = . (.)
RT = ()

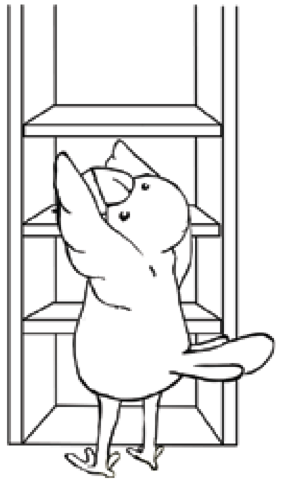

**NoH-distinction**
n =
hit rate = . (.)
RT = ()

**human-bird-distinction**
n =
hit rate = . (.)
RT = ()

**NoH-distinction** (pairwise comparison)
n =
hit rate = .88 (.33)
RT = 1895 (845)

**SSIM**
reference picture = 0.81
NoH-depiction = 0.68

## “shirt”

human; school age; boy

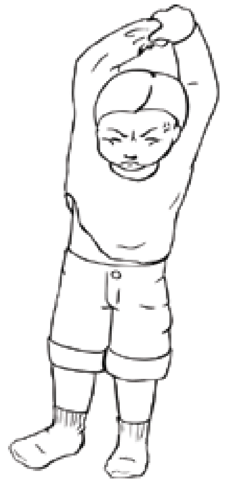

**NoH-distinction**
n = 64
hit rate = .69 (.47)
RT = 1438 (854)

**human-bird-distinction**
n = 66
hit rate = .97 (.17)
RT = 708 (413)

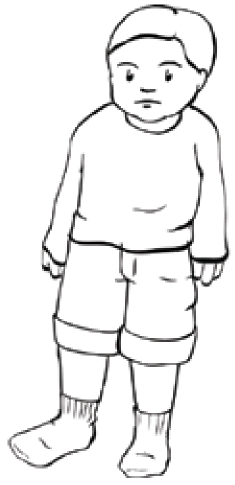

**NoH-distinction**
n = 61
hit rate = .74 (.44)
RT = 1436 (802)

**human-bird-distinction**
n = 66
hit rate = .86 (.35)
RT = 751 (474)

**NoH-distinction** (pairwise comparison)
n = 70
hit rate = .89 (.32)
RT = 1925 (796)

**SSIM**
reference picture = 1.00
NoH-depiction = 0.91

bird

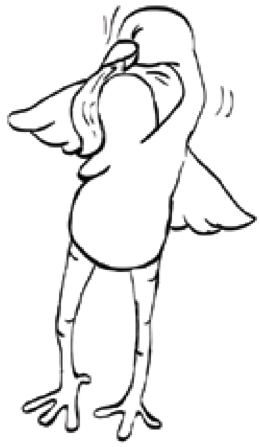

**NoH-distinction**
n = 41
hit rate = 1.00 (0.00)
RT = 1311 (876)

**human-bird-distinction**
n = 66
hit rate = .91 (.29)
RT = 760 (437)

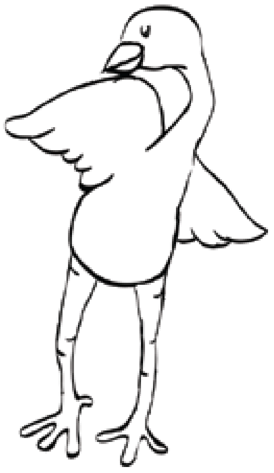

**NoH-distinction**
n = 65
hit rate = .78 (.41)
RT = 1392 (775)

**human-bird-distinction**
n = 66
hit rate = .95 (.21)
RT = 675 (402)

**NoH-distinction** (pairwise comparison)
n = 67
hit rate = .88 (.33)
RT = 2138 (837)

**SSIM**
reference picture = 0.90
NoH-depiction = 0.91

**"sit"**

human; toddler; boy

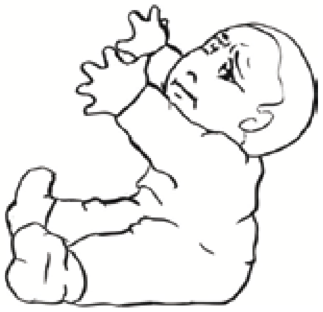

**NoH-distinction**
n = 64
hit rate = .72 (.45)
RT = 1245 (723)

**human-bird-distinction**
n = 68
hit rate = .90 (.31)
RT = 810 (520)

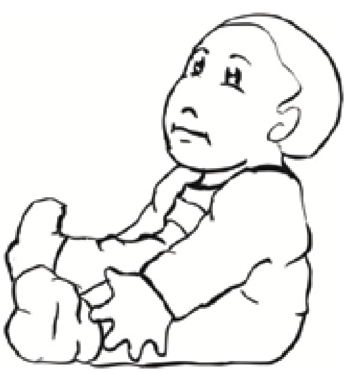

**NoH-distinction**
n = 66
hit rate = .67 (.48)
RT = 1478 (930)

**human-bird-distinction**
n = 70
hit rate = .93 (.26)
RT = 695 (441)

**NoH-distinction** (pairwise comparison)
n = 71
hit rate = .97 (.17)
RT = 1945 (764)

**SSIM**
reference picture = 1.00
NoH-depiction = 0.78

bird

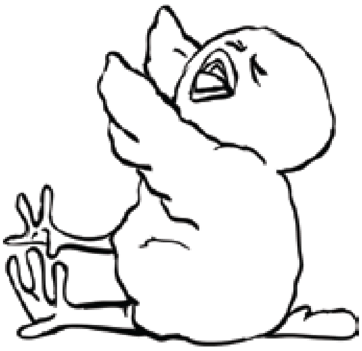

**NoH-distinction**
n = 51
hit rate = 1.00 (0.00)
RT = 1228 (766)

**human-bird-distinction**
n = 64
hit rate = .95 (.21)
RT = 811 (531)

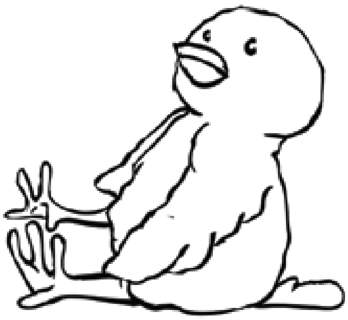

**NoH-distinction**
n = 57
hit rate = .70 (.46)
RT = 1423 (866)

**human-bird-distinction**
n = 63
hit rate = .94 (.25)
RT = 769 (525)

**NoH-distinction** (pairwise comparison)
n = 73
hit rate = .93 (.25)
RT = 1702 (703)

**SSIM**
reference picture = 0.84
NoH-depiction = 0.87

## **“stair”**

human; kindergaden age; boy

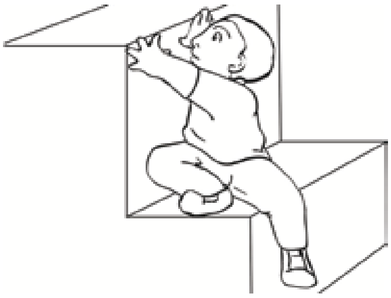

**NoH-distinction**
n = 70
hit rate = .79 (.41)
RT = 1110 (634)

**human-bird-distinction**
n = 68
hit rate = .94 (.24)
RT = 646 (335)

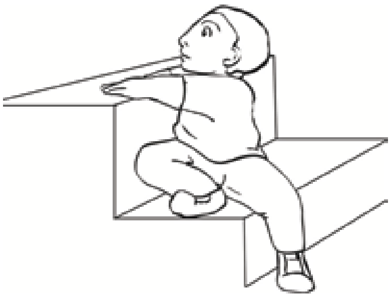

**NoH-distinction**
n = 63
hit rate = .49 (.50)
RT = 1346 (839)

**human-bird-distinction**
n = 70
hit rate = .94 (.23)
RT = 705 (395)

**NoH-distinction** (pairwise comparison)
n = 71
hit rate = .87 (.34)
RT = 1815 (589)

**SSIM**
reference picture = 0.90
NoH-depiction = 0.84

---

human; kindergarden age; girl

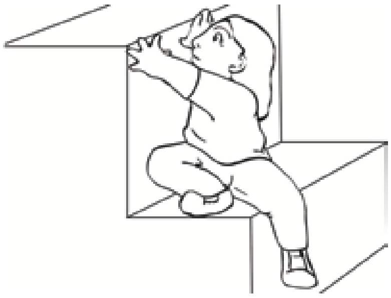

**NoH-distinction**
n = 66
hit rate = .83 (.38)
RT = 1347 (841)

**human-bird-distinction**
n = 64
hit rate = .91 (.29)
RT = 668 (441)

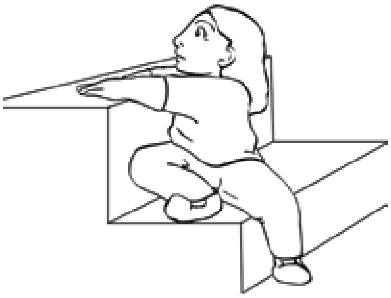

**NoH-distinction**
n = 66
hit rate = .61 (.49)
RT = 1364 (748)

**human-bird-distinction**
n = 67
hit rate = .88 (.33)
RT = 763 (464)

**NoH-distinction** (pairwise comparison)
n = 70
hit rate = .87 (.34)
RT = 1889 (672)

**SSIM**
reference picture = 1.00
NoH-depiction = 0.79

---

bird

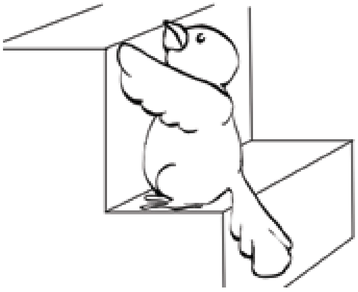

**NoH-distinction**
n = 49
hit rate = 1.00 (0.00)
RT = 1208 (786)

human-bird-distinction
n = 63
hit rate = .92 (.27)
RT = 738 (442)

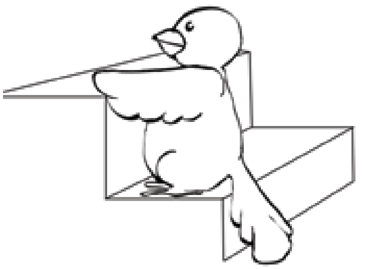

**NoH-distinction**
n = 63
hit rate = .56 (.50)
RT = 1110 (634)

**human-bird-distinction**
n = 69
hit rate = .93 (.26)
RT = 691 (384)

**NoH-distinction** (pairwise comparison)
n = 69
hit rate = .91 (.28)
RT = 1961 (739)

**SSIM**
reference picture = 0.98
NoH-depiction = 0.93

## “table”

human; toddler

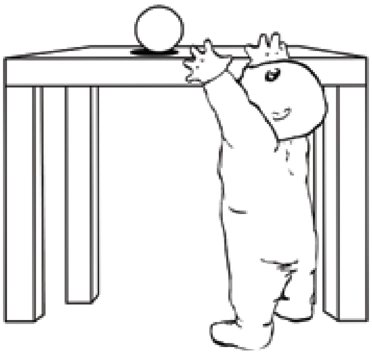

**NoH-distinction**
n = 65
hit rate = .86 (.35)
RT = 1176 (934)

**human-bird-distinction**
n = 65
hit rate = .91 (.29)
RT = 775 (417)

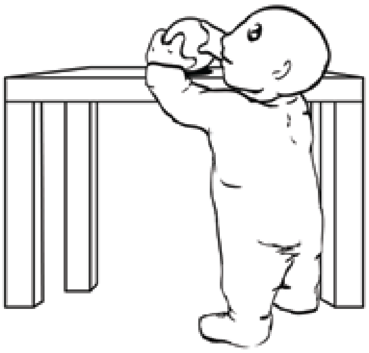

**NoH-distinction**
n = 63
hit rate = .78 (.42)
RT = 1240 (725)

**human-bird-distinction**
n = 64
hit rate = .94 (.24)
RT = 754 (399)

**NoH-distinction** (pairwise comparison)
n = 71
hit rate = .96 (.20)
RT = 1519 (653)

**SSIM**
reference picture = 1.00
NoH-depiction = 0.88

bird

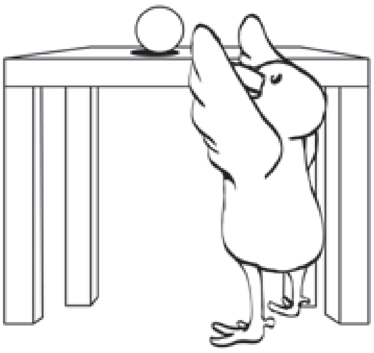

**NoH-distinction**
n = 53
hit rate = 1.00 (0.00)
RT = 961 (622)

**human-bird-distinction**
n = 70
hit rate = .87 (.34)
RT = 739 (460)

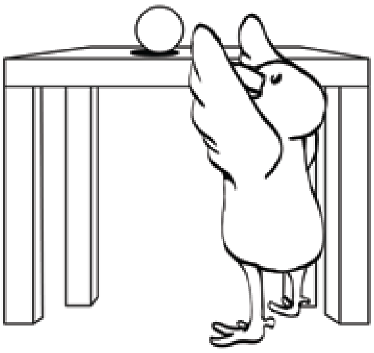

**NoH-distinction**
n = 62
hit rate = .74 (.44)
RT = 1185 (693)

**human-bird-distinction**
n = 63
hit rate = .87 (.34)
RT = 745 (443)

**NoH-distinction** (pairwise comparison)
n = 75
hit rate = .89 (.31)
RT = 1725 (706)

**SSIM**
reference picture = 0.86
NoH-depiction = 0.85

**“table_chair”**

human; kindergarden age; boy

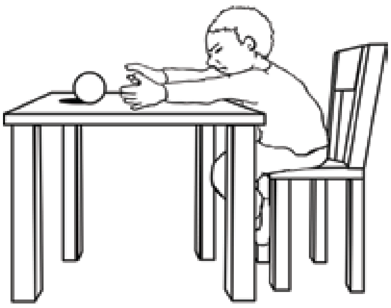

**NoH-distinction**
n = 63
hit rate = .87 (.34)
RT = 1052 (676)

**human-bird-distinction**
n = 62
hit rate = .95 (.22)
RT = 719 (405)

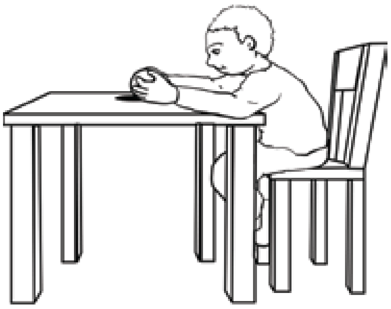

**NoH-distinction**
n = 67
hit rate = .84 (.37)
RT = 1334 (911)

**human-bird-distinction**
n = 69
hit rate = .94 (.24)
RT = 754 (411)

**NoH-distinction** (pairwise comparison)
n = 74
hit rate = . 96 (.20)
RT = 1588 (653)

**SSIM**
reference picture = 1.00
NoH-depiction = 0.81

bird

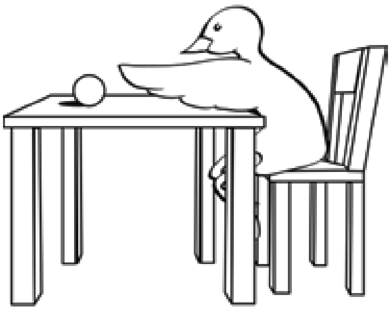

**NoH-distinction**
n = 63
hit rate = .86 (.35)
RT = 1250 (739)

human-bird-distinction
n = 66
hit rate = .89 (.31)
RT = 708 (457)

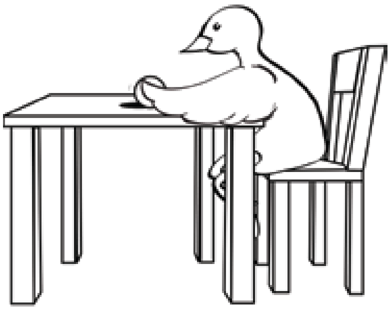

**NoH-distinction**
n = 67
hit rate = .84 (.37)
RT = 1334 (911)

**human-bird-distinction**
n = 71
hit rate = .97 (.17)
RT = 754 (445)

**NoH-distinction** (pairwise comparison)
n = 72
hit rate = .90 (.30)
RT = 1619 (655)

**SSIM**
reference picture = 0.84
NoH-depiction = 0.85

human; toddler

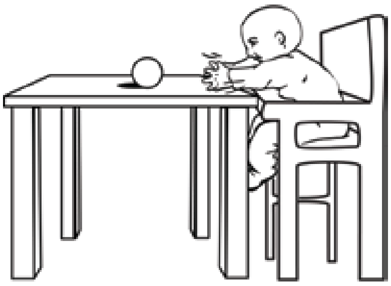

**NoH-distinction**
n = 67
hit rate = .88 (.33)
RT = 1043 (739)

**human-bird-distinction**
n = 66
hit rate = .97 (.17)
RT = 662 (349)

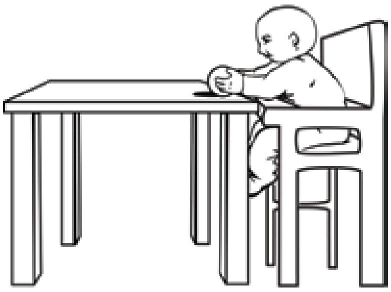

**NoH-distinction**
n = 58
hit rate = .84 (.37)
RT = 1266 (777)

**human-bird-distinction**
n = 70
hit rate = .91 (.28)
RT = 695 (425)

**NoH-distinction** (pairwise comparison)
n = 73
hit rate = . 92 (.28)
RT = 1616 (683)

**SSIM**
reference picture = 0.78
NoH-depiction = 0.86

bird; toddler-equivalent

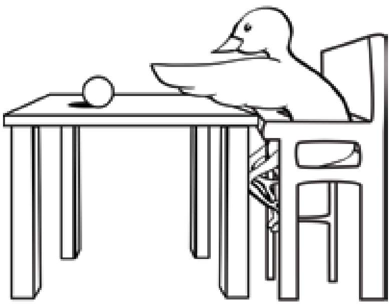

**NoH-distinction**
n = 56
hit rate = 1.00 (0.00)
RT = 1106 (707)

**human-bird-distinction**
n = 69
hit rate = .93 (.26)
RT = 695 (452)

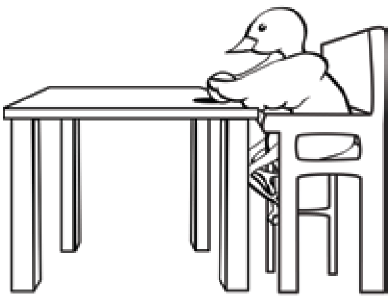

**NoH-distinction**
n = 65
hit rate = .85 (.36)
RT = 1217 (783)

**human-bird-distinction**
n = 65
hit rate = .95 (.21)
RT = 718 (487)

**NoH-distinction** (pairwise comparison)
n = 74
hit rate = . 96 (.20)
RT = 1563 (519)

**SSIM**
reference picture = 0.89
NoH-depiction = 0.84

human; kindergarden age; girl

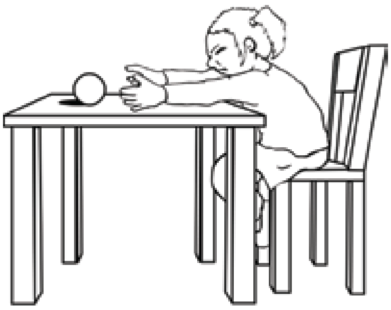

**NoH-distinction**
no data available yet

**human-bird-distinction**
no data available yet

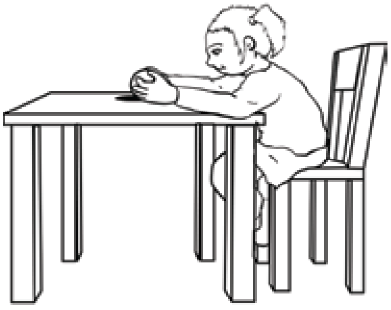

**NoH-distinction**
no data available yet

**human-bird-distinction**
no data available yet

**NoH-distinction** (pairwise comparison)
no data avalable yet

**SSIM**
reference picture = 0.83
NoH-depiction = 0.87
