# Supplementary material for: A New Standardized Stimulus Set for Studying Need-of-Help Recognition (NeoHelp)
Source: PLoS One. 2014 Jan 7;9(1):e84373. doi: 10.1371/journal.pone.0084373 (PMC3883661; doi:10.1371/journal.pone.0084373)
Supplement: Appendix S2 — Detailed description of the stimulus generation and the cover story. (PDF) [file pone.0084373.s002.pdf]

Supplementary Methods S2

Stimuli

Stimuli were created as follows: A reference picture for each situation was hand-drawn and converted into a black-and-white vector graphic using Adobe Illustrator CS 4. This picture format allowed us to create perceptually highly similar picture variations, most importantly picture pairs depicting need-of-help (NoH) and no-need-of-help (no-NoH), but also corresponding age and gender variations, as well as control pictures of birds. Birds were chosen as control animal pictures as their body shape enabled us to maintain highest possible picture similarity across both, NoH/no-NoH and human/animal picture pairs. Within each NoH/no-NoH picture pair differences only concerned those aspects of the picture that indicated NoH (e.g. child reaches for an apple, grasps it and thus succeeds in the attempt, or reaches for an apple, but it is too far away and the child's attempt fails). Analogous changes were made in order to create bird and human picture variations. No-NoH depictions of picture variations were created by transferring changes in NoH-picture features made in the original to the no-NoH variation picture (see Fig.1). Importantly, all stimulus conditions (NoH/no-NoH and human/bird) were created for each situation in order to ensure that the same stimulus material was usable across all three experimental paradigms.

Cover stories for each of the three experimental paradigms

At the beginning of each session, the children were introduce to Blobs, a nice alien preparing to land on earth. Before starting P1-bird, the experimenters explained that Blobs had lost his pet bird just before landing and needed help finding it. Therefore, the children needed to watch the flashing pictures carefully and indicate by pressing a button whether they saw a picture of a human, or of a bird (Blobs' lost pet). At the end of this scenario, children saw a happy Blobs reunited with his bird and were praised for helping him to find his pet.

Before starting P2-help, the experimenters explained that Blobs, while flying around Earth fast and catching only a glimpse of different situations, had difficulties understanding when someone on Earth needed help. Special care was taken to explain that it did not matter whether it was humans or birds who needed help, but that the general question was whether anyone needed help or not. So again, in order to help Blobs, children needed to watch the fast flashing pictures very carefully and indicate after each one by pressing a designated button whether it depicted someone in need of help or not. At the end of this scenario children were praised for doing a good job. Again, a picture of a happy and grateful Blobs was shown.

In P3-help-side, the experimenter explained to the children that Blobs was still confused about behavior on Earth and continued to have difficulties understanding if and when somebody needed help. Children had to indicate with a button press, whether the help-variation was shown on the left or the right side.
