# Supplementary material for: A New Standardized Stimulus Set for Studying Need-of-Help Recognition (NeoHelp)
Source: PLoS One. 2014 Jan 7;9(1):e84373. doi: 10.1371/journal.pone.0084373 (PMC3883661; doi:10.1371/journal.pone.0084373)
Supplement: Appendix S3 — Analyses of data for a subgroup of children presented with smaller pictures. The same analyses as presented in the main article were conducted in this subgroup. These analyses provide additional information for three situations of the NeoHelp stimulus set. (PDF) [file pone.0084373.s003.pdf]

Small Picture Analyses S3

In this supplementary report, we present additional data collected from a sample of 22 children (14 boys), who through random assignment saw a variation with smaller pictures. We will refer to this group as “small-picture subgroup” below, whereas the sample considered in the main article (60 children, 37 boys) will subsequently be referred to as “large-picture subgroup”. These analyses complement the results presented in our main paper and the same statistical procedures were used. We provide comparisons of effects and post-hoc differences between subgroups (Table S1), but limit the description of results for the small-picture subgroups to Figures and Tables. Since the small-picture subgroups consisted of only 22 children, the comparison with the large-picture subgroup (60 children) should be considered with caution.

Table S1. Comparison of analyses' results between picture-size subgroups

Analysis	Sub-analyses	Large-Picture subgroup	Small-Picture subgroup
Ambiguous situations	S3-help-side	climb	climb
	S2-help	none	gap
	S1-bird	none	none
Effects of task demands	hit rates	Main effect paradigm: P2 < P3 < P1	Main effect paradigm: P2 < P3; P2 < P1
		interaction paradigm x situation	interaction paradigm x situation
	RTs	Main effect paradigm: P1 < P2 < P3<br>interaction paradigm x situation	Main effect paradigm: P1 < P2 < P3<br>interaction paradigm x situation
Effects of stimulus content	Hit rates S3-help-side	Main effect situation:<br>gap < other situations	Main effect situation:<br>post-hoc differences n.s.
		main effect NoH-side	no main effect NoH-side
		interaction NoH-side x bird-depiction	no interaction
	RTs S3-help-side	Main effect of situation:<br>table_chair< other situations	Main effect of situation<br>table_chair< other situations
		Main effect bird-depiction:<br>bird- > human-depictions	Main effect bird-depiction:<br>bird- > human-depictions
		no effect of correctness	no effect of correctness
	Hit rates S2-help	All main effects significant:<br>gap and stair < other situations;<br>table_chair> other situations	All main effects significant:<br>no difference to gap and stair<br>table_chair> other situations
		NoH > no-NoH<br>bird- > human-depictions	NoH > no-NoH<br>bird- > human-depictions
		all 2-way interactions significant	interaction of NoH-depiction x bird-depiction and three-way interaction
	RTs S2-help	Main effect of situation	No main effect of situation
		main effect of NoH-depiction:<br>NoH < no-NoH	main effect of NoH-depiction:<br>NoH < no-NoH
		main effect correctness:<br>correct < incorrect responses	main effect correctness:<br>correct < incorrect responses
	Hit rates S1-bird	Main effect of bird-depiction:<br>bird- < human-depictions	Main effect of bird-depiction:<br>bird- > human-depictions
		main effect correctness:<br>correct < incorrect responses	main effect correctness:<br>correct < incorrect responses
		No interaction correctness x situation	interaction correctness x situation
	RTs S1-bird	No significant effects	No significant effects

Green shading indicates identical effects; orange shading indicates differences in effects due to non-significance in one of the picture-size subgroups, but an identical direction of absolute differences; red shading indicates differences in effects including their direction.

Comparison of participant populations in both picture-size subgroups

The total number of children included for analysis, distribution of gender and pet-ownership for the total sample, as well as the picture-size-subgroups are presented in Table S2.Systematic differences in demographic variables between picture-size subgroups were explored using a Wilcoxon rank sum test and exact binomial tests (π = estimated proportion of hits in the other subgroup). Age, gender and pet-ownership were not different between picture-size subgroups, $W = 55$, $p = .05$, $p = .79$ and $p = .83$, respectively (for percentages compare rows of Table S2).Thus, no systematic differences between picture size subgroup were observed for any of the demographic variables considered.

Table S2. Distribution of gender, age and pet-ownership for the sample and picture-size subgroups

Sample	Number of Children	Gender		Pet	
		Male	Female	Yes	No
Total	80	62.5 %	37.5 %	33.75 %	66.25 %
Large Pictures	60	59 %	41 %	34 %	66 %
Small Pictures	22	61.67 %	38.33 %	35 %	65 %

Note. Two children changed PCs, resulting in descriptive data for both picture-size subgroups

Ambiguous situations

Figure S1 shows estimated probabilities of success for each situation and paradigm when presented small. In sum, picture-size did not considerably influence the fact that pictures of almost all depicted situations (13 out of 15, marked with green outlines in all columns of Fig. S1 A-B) were unambiguously identifiable as depicting need-of-help (NoH).

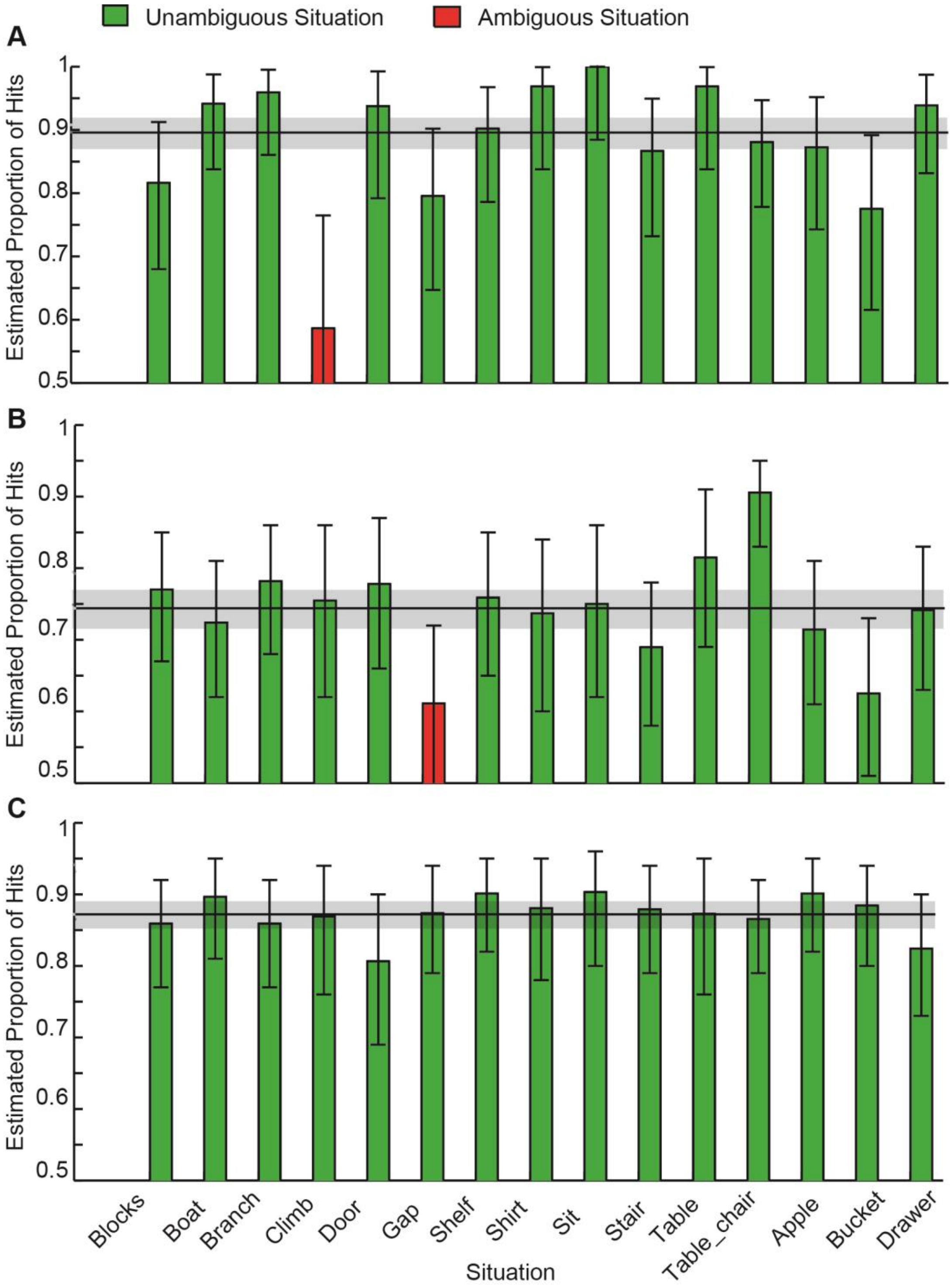

Figure S1. Estimated situation specific hit rates forP3-help-side (A), P2-help (B) and P1-bird (C). Estimates across variations of each situation are derived from tow-tailed exact binomial tests (π = .50). Green bars represent unambiguous situations (estimated hit rate significantly above .50) and red bars represent ambiguous situations (estimated hit rate not significantly

different from chance). Error bars represent upper and lower bounds of the estimated hit rates' confidence intervals (CIs). Overall estimated hit rates were calculated across all unambiguous situations. Lines represent overall estimated hit rates, shaded areas surrounding them indicate their CIs.

Highly similar results of binomial tests of hit rates for small- and large-picture subgroups (see Table S1) substantiate the conclusion that children perceived the difference between no-NoH- and NoH-depictions in all but two situations, regardless of whether they saw larger or smaller variations of the stimuli. Moreover, all three situations that were only correctly displayed in smaller size were unambiguous regarding need-of-help as well as human-/animal content in all three paradigms (see rightmost three bars in Fig. S1 A-C).

Changes in hit rates and RTs according to task demands

Detailed results of the ANOVA conducted for the small-picture subgroup are given in Table S3. As opposed to the analysis of the large-picture subgroup the mean hit rates of the small picture subgroup did not differ significantly between P1-bird (bird-human-distinction required) and P3-help-side. However, the remaining differences pointed into the same direction: P2-help was the paradigm with lowest accuracy. The pattern revealed by the main effect of *paradigm* with regard to RTs, (P1-bird <P2-help <P3-help-side) emerged in both picture size subgroups alike and thus irrespective of picture size. As can be seen in Table S1 differences in effects of task demands between picture-size subgroups were restricted to the absence of one post-hoc difference.

Table S3. Results of ANOVAs investigating the influence of *paradigm* on hit rates and RTs

		Hit Rates		RTs	
**Source**	**df**	**F**	**p**	**F**	**p**
Situation	14	1.40	.14	2.23**	< .01
Paradigm	2	40.76***	< .001	465.19***	< .001
Situation x Paradigm	26	1.56*	.04	1.79**	< .01

*** p< .001; **p < .01; *p < .05

Effects of stimulus content on hit rates and RTs

The detailed results of the ANOVAs considering S1-bird for both, hit rates and RTs are given in Table S4.The pattern of effects on RTs was the same in large- and small-picture subgroups (see Table S1). Regarding hit rates, the same factor *bird-depiction* yielded the only significant effect in both picture-size subgroups while no interactions were found in either picture-size subgroup.

Table S4. Results of ANOVAs conducted in P1-bird

		Hit Rates		RTs	
**Source**	**df**	**F**	**p**	**F**	**p**
Situation	14	0.51	.93	1.43	.13
NH-depiction	1	0.69	.40	0.67	.41
Bird-depiction	1	5.66*	.02	0.19	.66
Situation x NH-depiction	14	0.86	.61	0.65	.82
Situation x bird-depiction	1	0.73	.74	0.56	.90
NH-side x bird-depiction	1	1.37	.24	0.02	.90
Situation x NH-depiction x bird-depiction	14	1.10	.35	0.85	.62

*p < .05

However, the main effect of *bird-depiction* had opposite directions for the large-picture and small-picture subgroups. Birds were categorized more accurately in the large-picture subgroup whereas humans were more accurately categorized in the small-picture subgroup. This inversion

of *bird-depiction*'s main effect might arise due to differences in task difficulty according to *picture-size*: Hit rates were generally higher in the large-picture subgroup and on average above 92%, indicating that a ceiling effect could have played a role in changing the direction of the effect of *bird-depiction* on hit rates.

The detailed results of the ANOVAs considering S2-help for both, hit rates and RTs are given in Table S5. Mean RTs and hit rates for the 14 situations remaining in analysis are shown in Figure S2. We did not find pronounced differences in main effects between the small-picture and large-picture subgroups in P2-help.

Table S5. Results of ANOVAs conducted in S-2-help

		**Hit Rates**		**RTs**	
**Source**	**df**	**F**	**p**	**F**	**p**
Situation	13	2.31**	< .01	1.13	.33
NoH-depiction	1	145.19***	< .001	10.83**	< .01
Bird-depiction	1	7.44**	< .01	0.04	.85
Situation x NoH-depiction	13	1.69	.06	1.16	.31
Situation x bird-depiction	1	0.89	.56	0.54	.90
NoH-depiction x bird-depiction	1	8.14**	< .01	0.68	.41
Situation x NoH-depiction x bird-depiction	13	1.98*	.02	0.58	.87

***p < .001; **p < .01

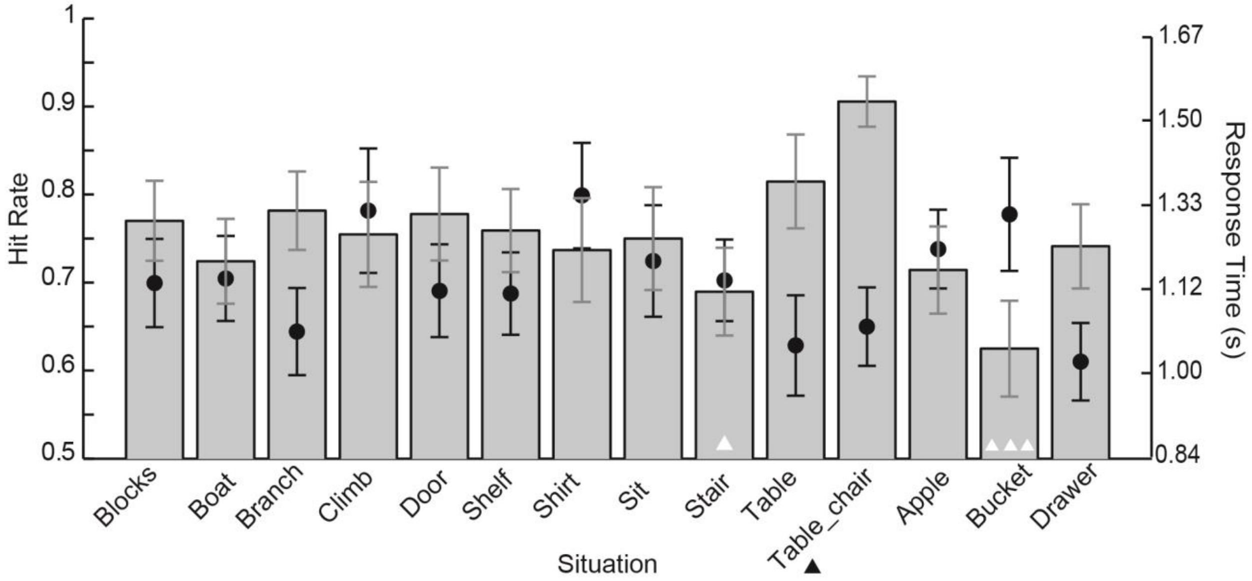

Figure S2.Response characteristics per situation for the small-picture subgroup in P2-help. Hit rates (bars) and mean RTs (dots) were calculated across all variations of a given situation. White triangles mark significant differences in hit rates compared to the situation "table_chair", their number corresponding to p-values of post-hoc Tukey HSD tests (one: $p < .05$, three: $p < .001$). Error bars represent SEM.

Divergences in effect patterns regarding hit rates and RTs were restricted to the absence of some significant interactions and one main effect in the small picture size subgroup (see Table S1), which was also considerably smaller in sample size (N = 22) than the large-picture subgroup (N = 60). Thus, divergences in effect patterns between picture-size subgroups are likely due to the smaller sample size, since all effects for both pictures size-subgroups pointed in the same direction, including the pattern revealed by the interaction of NoH-depiction and bird-depiction (see Table S6).

Table S6. Mean hit rates and SDs illustrating the interaction of *bird-depiction* and *NoH-depiction* in the small-picture subgroup for P2-help.

		NoH-depiction	
		NoH	No-NoH
Bird-	Human	.83 (.38)	.61 (.49)
depiction	Bird	1.00 (.00)	.61 (.49)

Note. NoH = need-of-help. Numbers in brackets represent SDs.

The detailed results of the ANOVAs considering S3-help-side for both, hit rates and RTs are given in Table S7. Mean RTs and hit rates for the 14 situations remaining in analysis are shown in Figure S3.Effect patterns found in the large and small picture-size subgroups in paradigmP3-help were highly similar (see Table S1). Given that considerably fewer children (N = 22) were assigned to the small picture-size subgroup and that the only differences were restricted to a lack of significant main effects and interactions in this subgroup, it is likely that content-related picture properties' influences on response characteristics in P3-help-side were independent of picture size. What is more, we also found that differences between situations were similar in both subgroups, the only discrepancy being again that some differences did not reach significance in the smaller subgroup.

Table S7. Results of ANOVAs conducted in P3-help-side

		Hit Rates		RTs	
**Source**	**df**	**F**	**p**	**F**	**p**
Situation	13	2.09*	.01	3.68***	< .001
NH-side	1	0.40	.53	0.16	.69
Bird-depiction	1	0.35	.56	4.35*	.04
Situation x NH-side	13	0.40	.97	1.70	.06
Situation x bird-depiction	1	0.53	.91	0.84	.75
NH-side x bird-depiction	1	0.23	.63	0.10	.99
Situation x NH-side x bird-depiction	13	0.96	.49	0.30	.62

***p < .001; *p < .05

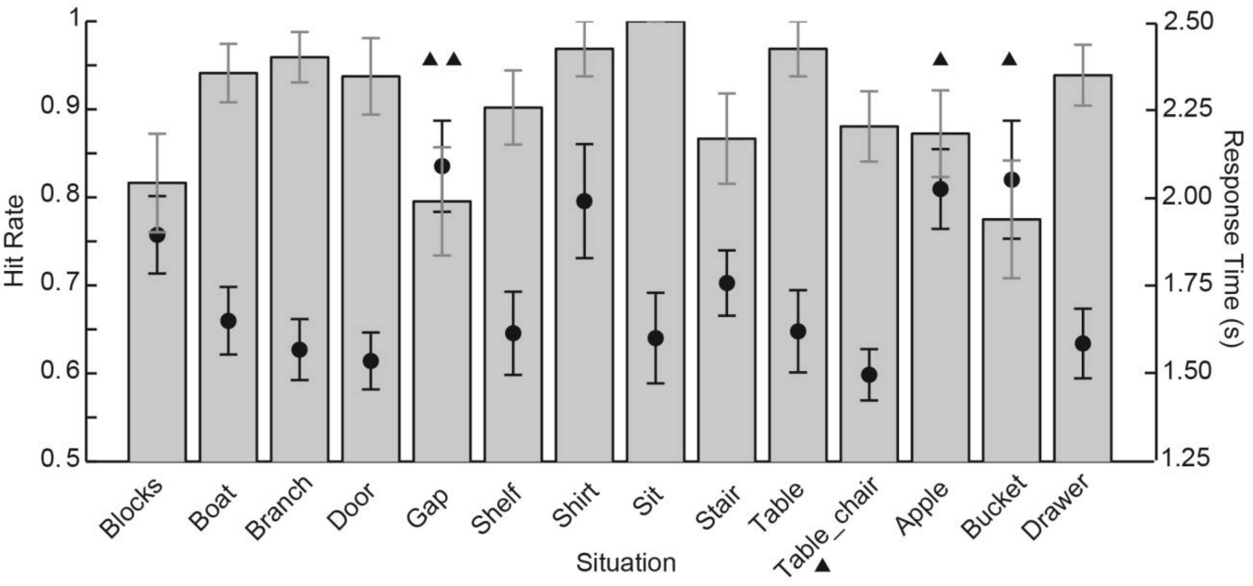

Figure S3.Response characteristics per situation for the small-picture subgroup in P3-help-side. Hit rates (bars) and mean RTs (dots) were calculated across all variations of a given situation. Symbols indicate significant differences in RTs between the situation “table_chair” and others. The number of symbols corresponds to p-values of post-hoc Tukey HSD tests (one: p < .05, two: p < .01). Error bars represent SEM.
