# Supplementary material for: A New Standardized Stimulus Set for Studying Need-of-Help Recognition (NeoHelp)
Source: PLoS One. 2014 Jan 7;9(1):e84373. doi: 10.1371/journal.pone.0084373 (PMC3883661; doi:10.1371/journal.pone.0084373)
Supplement: Appendix S4 — Analyses of picture variations and paradigm sequence. Detailed statistics regarding the influence of variation in the human depictions and the sequence of paradigms P1-bird and P2-help are provided. Whereas no differences between human variations of one situation were observed, different paradigm sequences resulted in different hit rates and mean RTs. (PDF) [file pone.0084373.s004.pdf]

Supplementary Analyses S4

Here we present additional analyses that are not directly related to the main questions of the paper regarding suitability of the NeoHelp stimulus set for use in empirical research but which can be helpful when evaluating individual pictures, situations or when deciding on the appropriate presentation side and presentation order. In addition to reporting the results of the study population included in the main paper (60 children), we include information on the data of the 22 children (14 boys) who through random assignment saw a variation with smaller pictures. We will refer to this group as the “small-picture subgroup”, whereas the sample considered in the main article (60 children, 37 boys) will subsequently be referred to as “large-picture subgroup”.

Picture Variations

For ten of the 15 situations comprising the NeoHelp Stimulus Set, additional variations of the human depictions (altering gender or age or race) were developed. As these variations could theoretically contribute to differences of compared means, we analyzed whether it is acceptable to cluster picture variations according to the situation they depicted by comparing mean hit rates and RTs to pictures of those 10 situations for which variations (gender, age or race) were available. Data from bird depictions was excluded from this analysis, as there were no equivalent variations. Thus, 12 separate 10 x 2 (*situation* x *picture-variation*) ANOVAs were conducted for each paradigm (3) and each picture-size subgroup (2) for hit rates (6) as well as RTs (6).

Supplementary Tables S8, S9 and S10 list the detailed results of the ANOVAs conducted for each paradigm. Regardless of picture-size subgroup and paradigm, *picture-variation* within a situation never had an effect on either hit rates or RTs and also never interacted with *situation*. Therefore, we combined the means within each situation across its variations in all subsequent analyses, thus increasing the number of trials per situation mean.

Table S8. Results of ANOVAs investigating the influence of *picture-variation* in P3-help-side

	Large-Picture Subgroup					Small-Picture Subgroup				
		Hit Rates		RTs			Hit Rates		RTs	
**Source**	**df**	**F**	**p**	**F**	**p**	**df**	**F**	**p**	**F**	**p**
Situation	6	6.36***	< .001	4.49***	< .001	9	1.41	0.18	3.194***	< .01
Picture-variation	1	0.51	.48	0.45	0.50	1	0.16	0.69	0.80	0.37
NoH-side	1	0.10	.75	1.11	0.29	1	0.38	0.54	0.79	0.38
Situation x picture-variation	6	0.29	.94	0.90	0.50	9	1.30	0.24	0.62	0.78
Situation x NoH-side	6	1.12	.35	2.04	0.06	9	0.25	0.99	0.82	0.60
Picture-variation x NoH-side	1	0.65	.42	0.87	0.35	1	1.14	0.29	3.44	0.06
Situation x picture-variation x NoH-side	6	0.36	.90	0.48	0.83	9	0.29	0.98	0.91	0.52

***p < .001

Table S9. Results of ANOVAs investigating the influence of *picture-variation* in P2-help

	Large-Picture Subgroup					Small-Picture Subgroup				
		Hit Rates		RTs			Hit Rates		RTs	
**Source**	**df**	**F**	**p**	**F**	**p**	**df**	**F**	**p**	**F**	**p**
Situation	6	6.80***	< .001	3.187***	< .001	9	2.25*	.02	1.00	.43
Picture-variation	1	0.02	.90	0.041	.84	1	0.78	.38	0.07	.79
NoH-depiction	1	17.44***	< .001	22.309***	< .001	1	33.16***	< .001	2.74	.10
Situation x picture-variation	6	0.89	.50	0.604	.73	9	0.73	.69	0.64	.77
Situation x NoH-depiction	6	1.35	.23	0.977	.44	9	2.44*	.01	0.56	.83
Picture-variation x NoH-depiction	1	0.62	.43	0.427	.51	1	0.67	.41	0.01	.91
Situation x picture-variation x NoH-depiction	6	0.69	.66	0.976	.44	9	0.82	.60	1.17	.31

*** p < .001; **p < .01; *p < .05

Table S10. Results of ANOVAs investigating the influence of *picture-variation* in P1-bird

	Large-Picture Subgroup					Small-Picture Subgroup				
		Hit Rates		RTs			Hit Rates		RTs	
**Source**	**df**	**F**	**p**	**F**	**p**	**df**	**F**	**p**	**F**	**p**
Situation	6	1.02	.41	1.30	.25	9	0.74	.68	1.29	.24
Picture-variation	1	0.04	.84	1.17	.28	1	0.20	.66	0.15	.70
NoH-depiction	1	4.90	.03	1.00	.32	1	0.27	.61	1.75	.19
Situation x picture-variation	6	1.11	.35	0.19	.98	9	0.69	.72	0.91	.52
Situation x NoH-depiction	6	1.99	.06	2.48	.02	9	1.27	.25	1.16	.32
Picture-variation x NoH-depiction	1	0.18	.67	0.00	.96	1	0.32	.57	0.18	.67
Situation x picture-variation x NoH-depiction	6	0.92	.48	0.28	.95	9	0.99	.45	0.88	.55

Effects of paradigm sequence on hit rates and RTs

As it is plausible that not only prior exposure to pictures of the stimulus set can affect response characteristics but also that this effect can be modified by the task demands, we assessed whether response characteristics were influenced by the sequence in which children absolved paradigms. We employed 12 x 2 (*situation* x *paradigm sequence*) ANOVAs. Only data of children who absolved all three paradigms is included here (N = 66). Separate ANOVAs were calculated for each paradigm and each picture-size subgroup. Even though included as factor in the ANOVAs, we will not report main effects of *paradigm* separately, here, as they followed the same pattern as reported in the main article. Detailed results of all ANOVAs conducted are shown in Tables S 11-13.

Table S11. Results of ANOVAs investigating the influence of *scenario sequence* on hit rates and RTs in S3-help-side

	**Large-Picture Subgroup**					**Small-Picture Subgroup**				
		**Hit Rates**		**RTs**			**Hit Rates**		**RTs**	
**Source**	**df**	**F**	**p**	**F**	**p**	**df**	**F**	**p**	**F**	**p**
Situation	10	3.89***	< .001	6.44***	< .001	13	2.50**	< .01	4.54***	< .001
Scenario sequence	1	8.36**	< .01	5.62*	.02	1	1.25	.26	7.33**	< .01
Situation x Scenario sequence	10	0.98	.46	0.65	.77	13	2.72***	< .001	1.56	.09

*** p < .001; **p < .01; *p < .05

Table S12. Results of ANOVAs investigating the influence of *scenario sequence* on hit rates and RTs in S2-help

	**Large-Picture Subgroup**					**Small-Picture Subgroup**				
		**Hit Rates**		**RTs**			**Hit Rates**		**RTs**	
**Source**	**df**	**F**	**p**	**F**	**p**	**df**	**F**	**p**	**F**	**p**
Situation	11	6.01***	< .001	4.48***	< .001	13	1.75*	.05	1.60	.08
Scenario sequence	1	9.99**	< .01	10.06**	< .01	1	1.12	.29	7.13**	< .01
Situation x Scenario sequence	11	0.49	.91	0.62	.81	13	0.85	.60	0.95	.50

*** p < .001; **p < .01; *p < .05

Table S13. Results of ANOVAs investigating the influence of *scenario sequence* on hit rates and RTs in S1-help

	**Large-Picture Subgroup**					**Small-Picture Subgroup**				
		**Hit Rates**		**RTs**			**Hit Rates**		**RTs**	
**Source**	**df**	**F**	**p**	**F**	**p**	**df**	**F**	**p**	**F**	**p**
Situation	11	0.94	.50	1.16	.31	14	0.20	1.00	1.30	.20
Scenario sequence	1	7.70**	< .01	0.10	.75	1	1.80	.18	20.24***	< .001
Situation x Scenario sequence	11	1.53	.11	1.33	.20	14	0.50	.93	0.27	1.00

*** p < .001; **p < .01

Effects of *paradigm sequence* on hit rates and RTs of the large-picture subgroup are illustrated in the top row of Figure S4, effects for the small-picture subgroup in the bottom row of Figure S4. In sum, *paradigm sequence* had consistent effects on response characteristics across picture-size subgroups. *Paradigm sequence* interacted with a picture content related factor only once, i.e. regarding hit rates in P3-help in the small-picture size subgroup. Thus, the main effect of *paradigm sequence* can primarily be regarded as a superimposed factor influencing response characteristics, not influencing analyses of picture content.

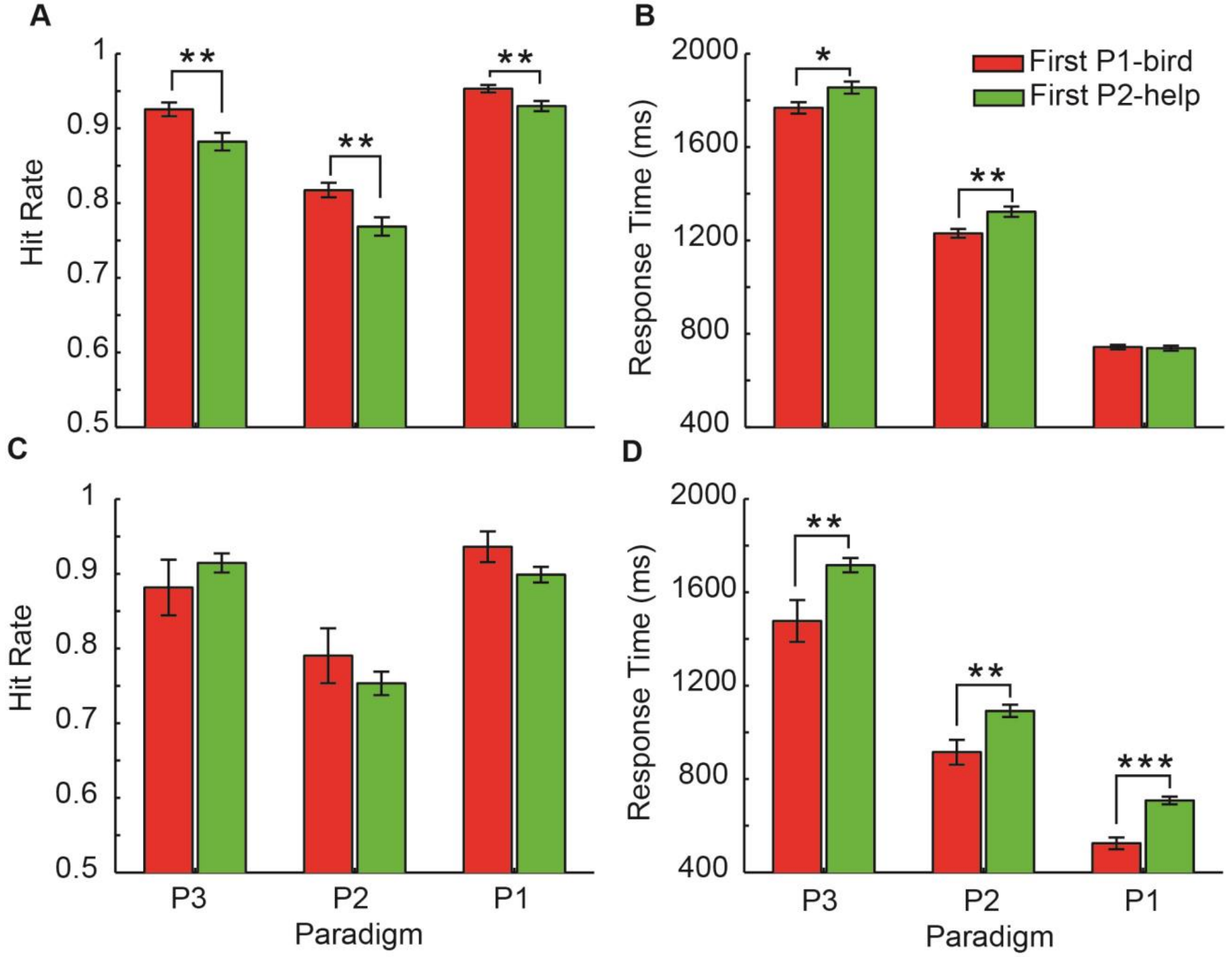

Figure S4. Hit rates and mean RTs according to paradigm sequence. Red bars represent data of children who did P1-bird first, green bars of children who did P2-help first. Data for the large-picture subgroup is shown on top (A, B), data for the small-picture subgroup is shown on the bottom (C, D). Hit rates are shown in the left graphs (A, C), RTs in the right graphs (B, D). Asterisks mark significant differences as indicated by post-hoc Tukey HSD tests: ***$p < .001$; **$p < .01$; *$p < .05$. Note that significant main effects of *paradigm sequence* point into the same direction in both picture-size subgroups for hit rates and RTs.
