# Supplementary figures and images for: A New Standardized Stimulus Set for Studying Need-of-Help Recognition (NeoHelp)
Source: PLoS One. 2014 Jan 7;9(1):e84373. doi: 10.1371/journal.pone.0084373 (PMC3883661; doi:10.1371/journal.pone.0084373)

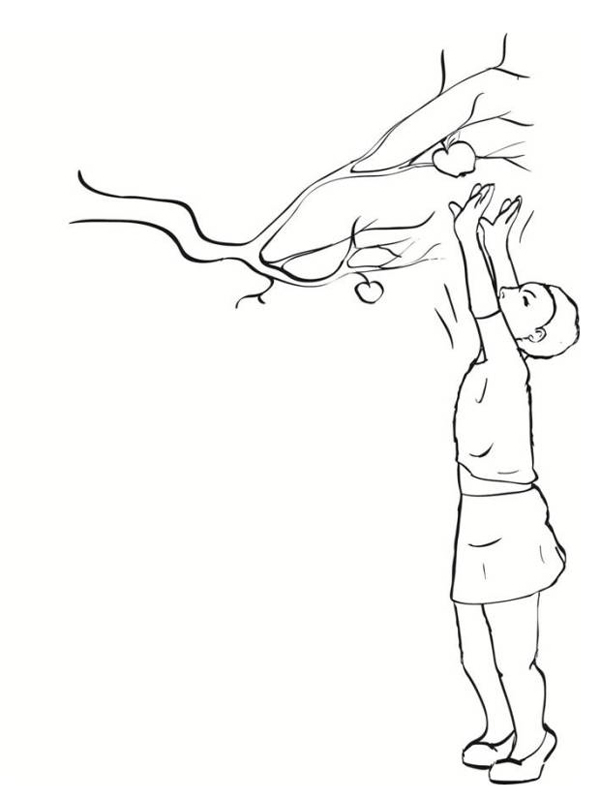

Supplement: Appendix S5 — All NeoHelp stimuli in JPEG format. All stimuli are provided as used in the study reported. (ZIP) [file pone.0084373.s005.zip › Stimuli/apple.jpg]

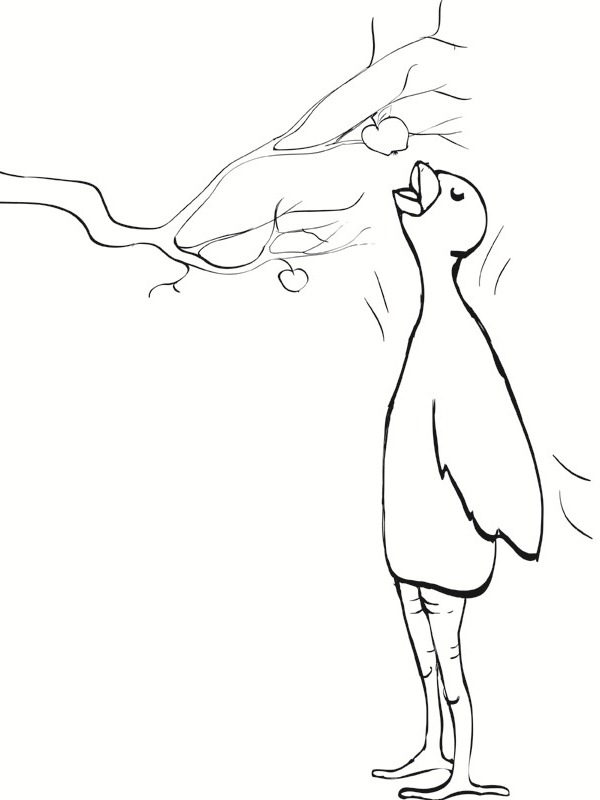

Supplement: Appendix S5 — All NeoHelp stimuli in JPEG format. All stimuli are provided as used in the study reported. (ZIP) [file pone.0084373.s005.zip › Stimuli/apple_bird.jpg]

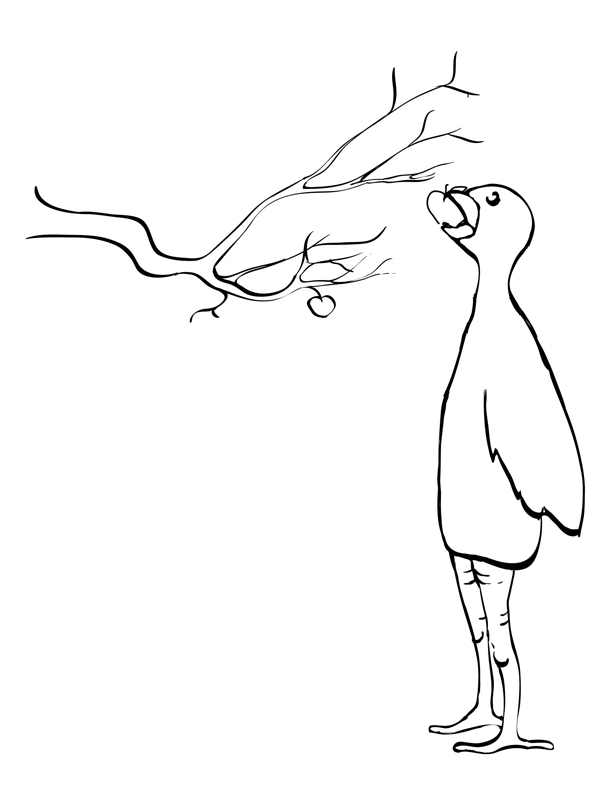

Supplement: Appendix S5 — All NeoHelp stimuli in JPEG format. All stimuli are provided as used in the study reported. (ZIP) [file pone.0084373.s005.zip › Stimuli/apple_bird_success.jpg]

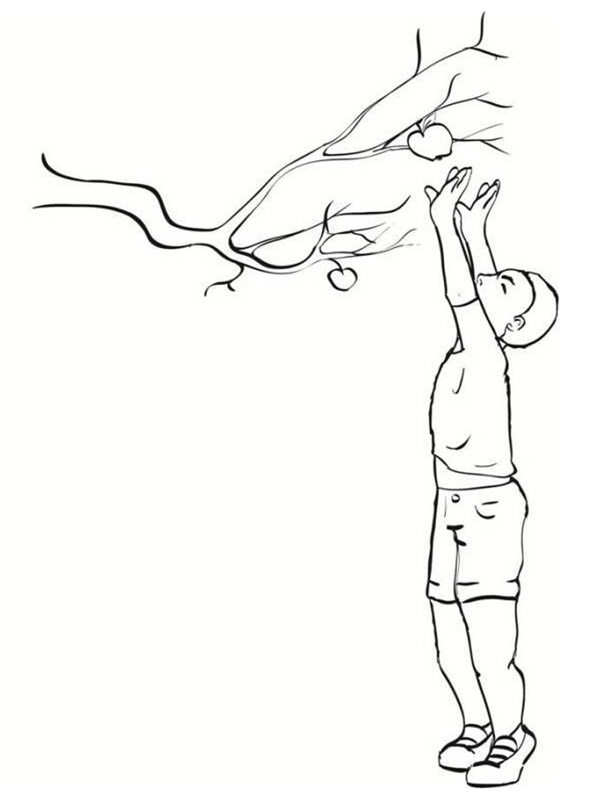

Supplement: Appendix S5 — All NeoHelp stimuli in JPEG format. All stimuli are provided as used in the study reported. (ZIP) [file pone.0084373.s005.zip › Stimuli/apple_boy.jpg]

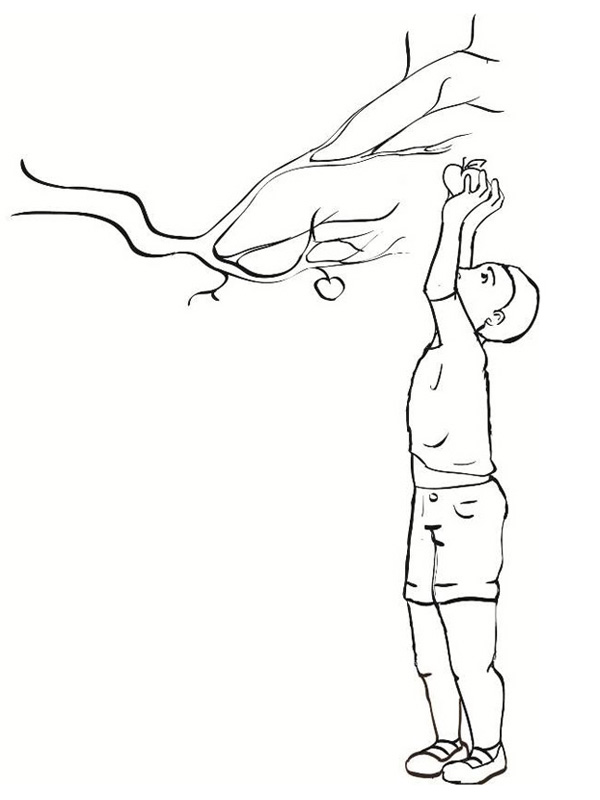

Supplement: Appendix S5 — All NeoHelp stimuli in JPEG format. All stimuli are provided as used in the study reported. (ZIP) [file pone.0084373.s005.zip › Stimuli/apple_boy_success.jpg]

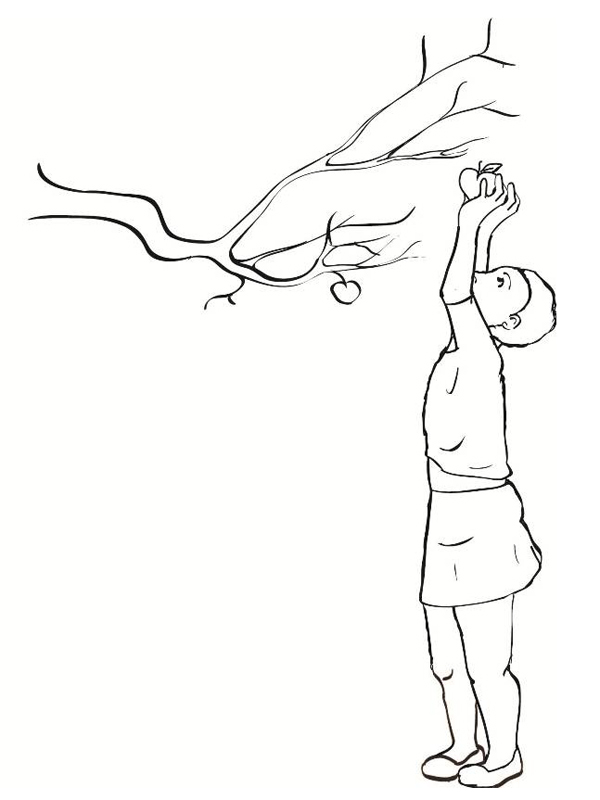

Supplement: Appendix S5 — All NeoHelp stimuli in JPEG format. All stimuli are provided as used in the study reported. (ZIP) [file pone.0084373.s005.zip › Stimuli/apple_success.jpg]

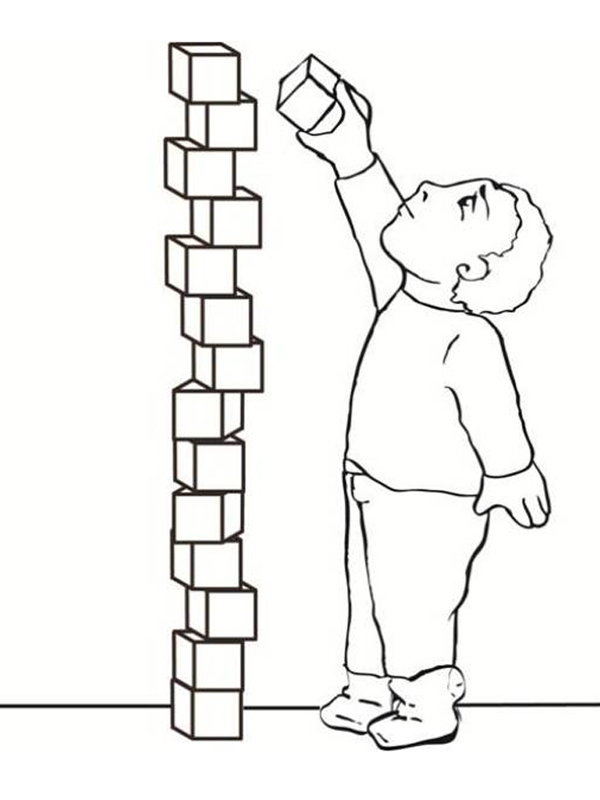

Supplement: Appendix S5 — All NeoHelp stimuli in JPEG format. All stimuli are provided as used in the study reported. (ZIP) [file pone.0084373.s005.zip › Stimuli/blocks.jpg]

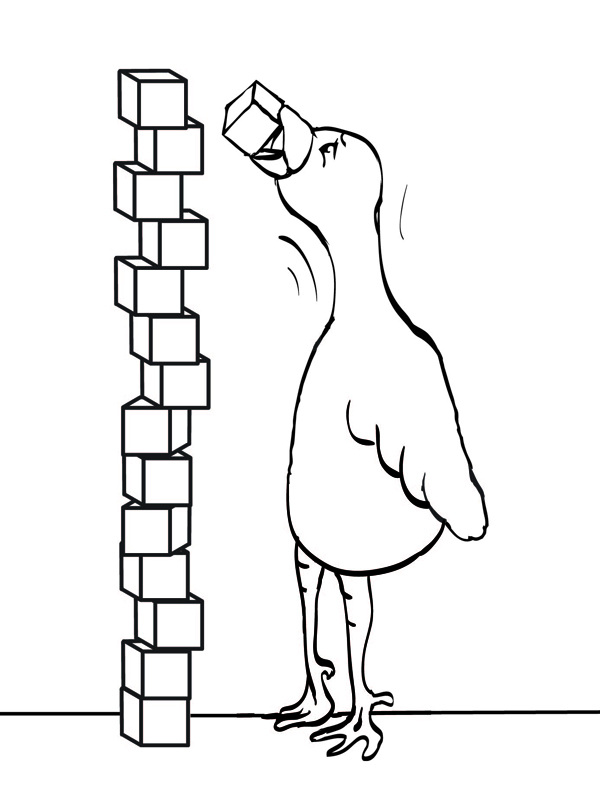

Supplement: Appendix S5 — All NeoHelp stimuli in JPEG format. All stimuli are provided as used in the study reported. (ZIP) [file pone.0084373.s005.zip › Stimuli/blocks_bird.jpg]

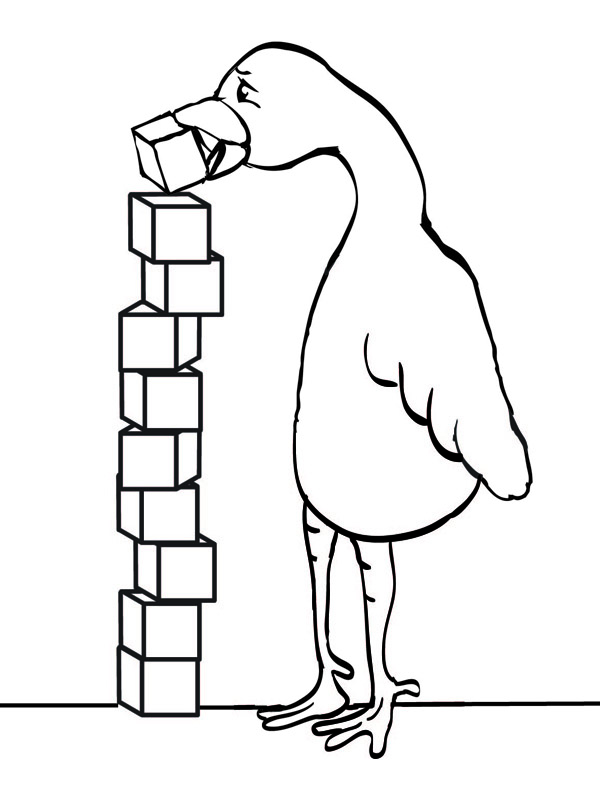

Supplement: Appendix S5 — All NeoHelp stimuli in JPEG format. All stimuli are provided as used in the study reported. (ZIP) [file pone.0084373.s005.zip › Stimuli/blocks_bird_success.jpg]

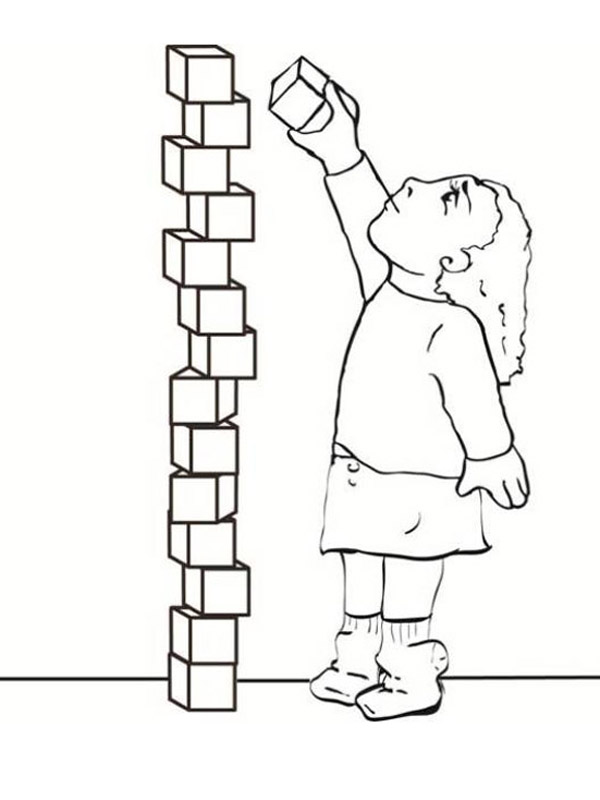

Supplement: Appendix S5 — All NeoHelp stimuli in JPEG format. All stimuli are provided as used in the study reported. (ZIP) [file pone.0084373.s005.zip › Stimuli/blocks_girl.jpg]

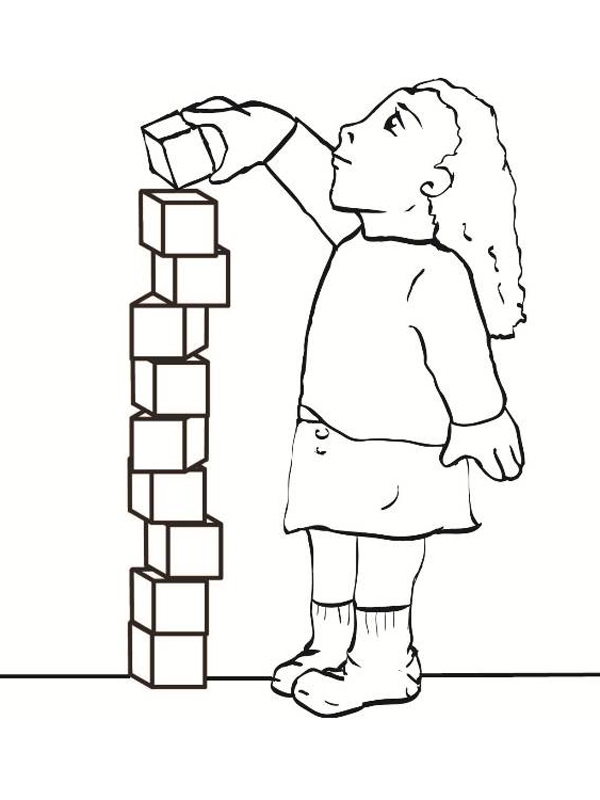

Supplement: Appendix S5 — All NeoHelp stimuli in JPEG format. All stimuli are provided as used in the study reported. (ZIP) [file pone.0084373.s005.zip › Stimuli/blocks_girl_success.jpg]

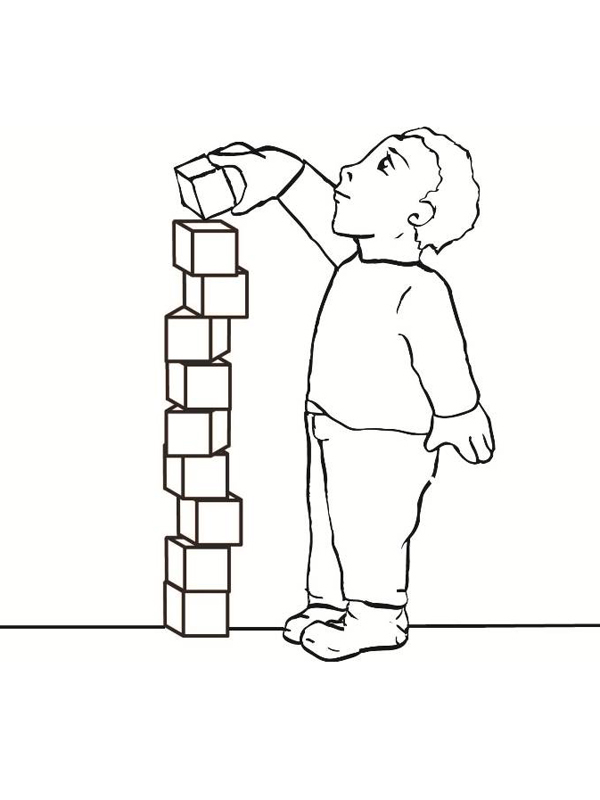

Supplement: Appendix S5 — All NeoHelp stimuli in JPEG format. All stimuli are provided as used in the study reported. (ZIP) [file pone.0084373.s005.zip › Stimuli/blocks_success.jpg]

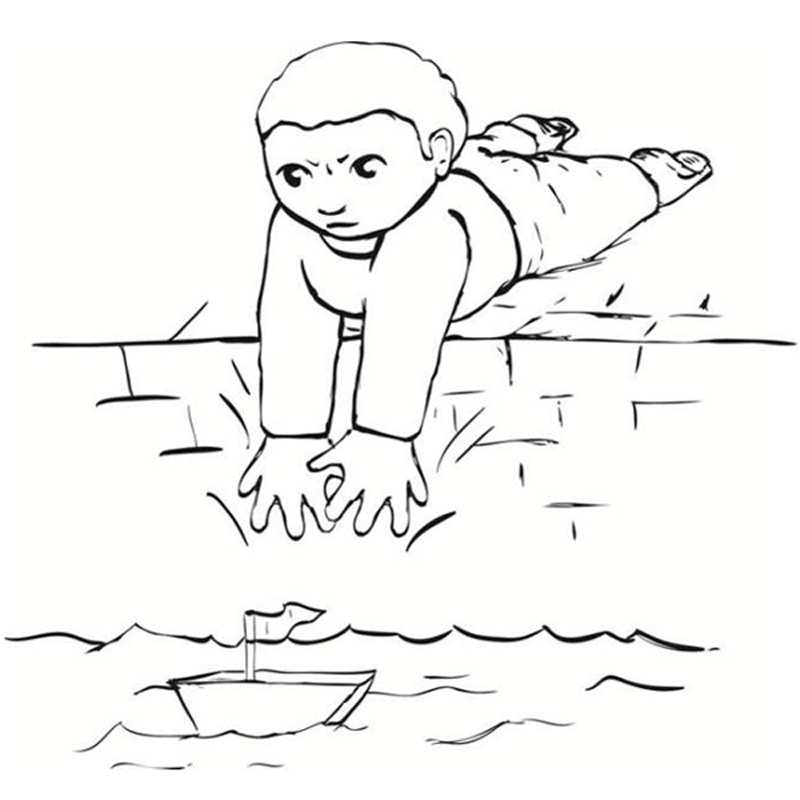

Supplement: Appendix S5 — All NeoHelp stimuli in JPEG format. All stimuli are provided as used in the study reported. (ZIP) [file pone.0084373.s005.zip › Stimuli/boat.jpg]

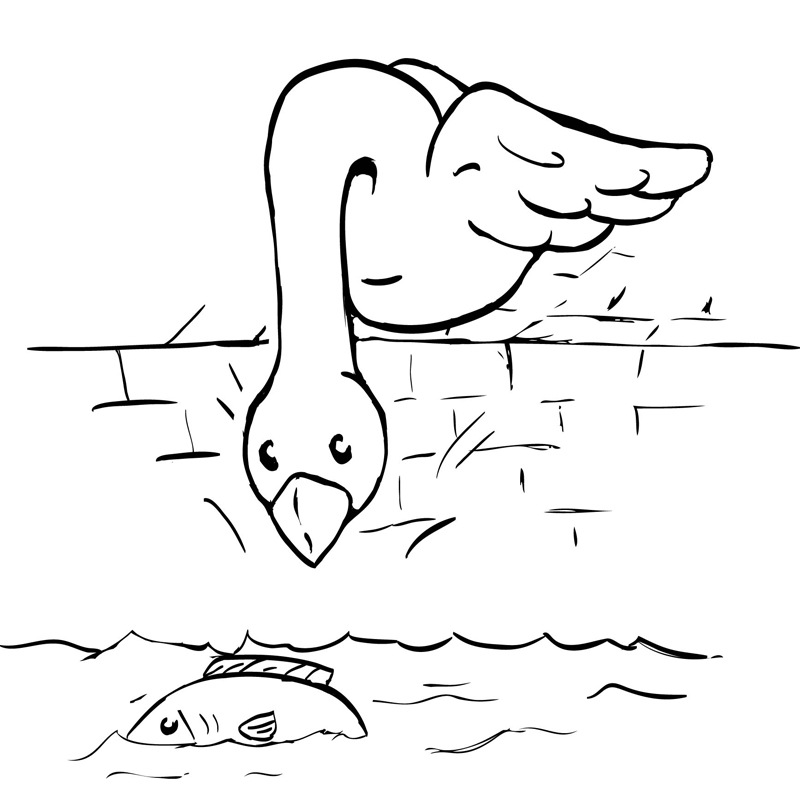

Supplement: Appendix S5 — All NeoHelp stimuli in JPEG format. All stimuli are provided as used in the study reported. (ZIP) [file pone.0084373.s005.zip › Stimuli/boat_bird.jpg]

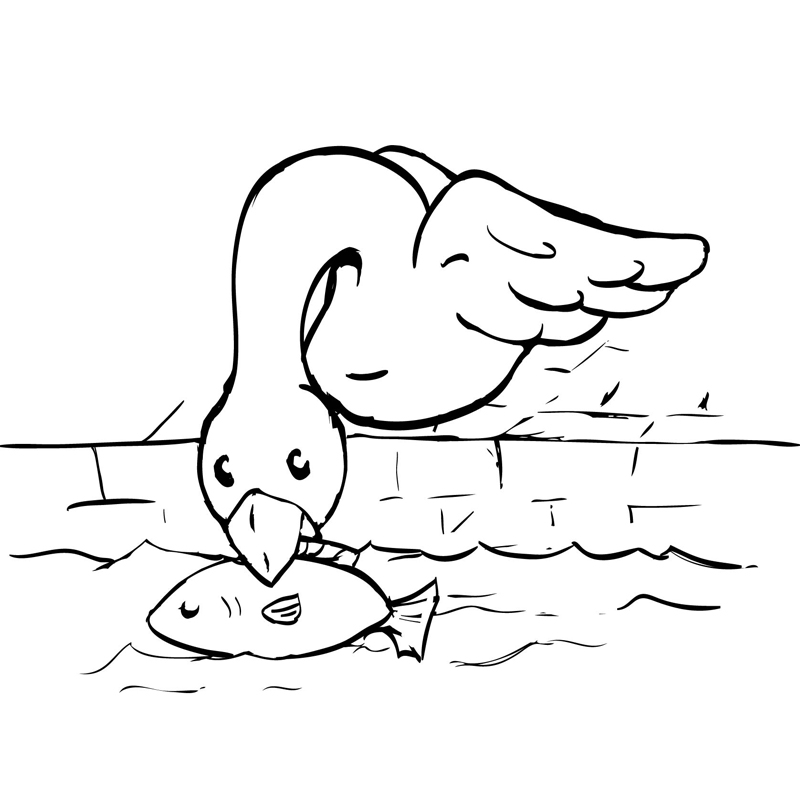

Supplement: Appendix S5 — All NeoHelp stimuli in JPEG format. All stimuli are provided as used in the study reported. (ZIP) [file pone.0084373.s005.zip › Stimuli/boat_bird_success.jpg]

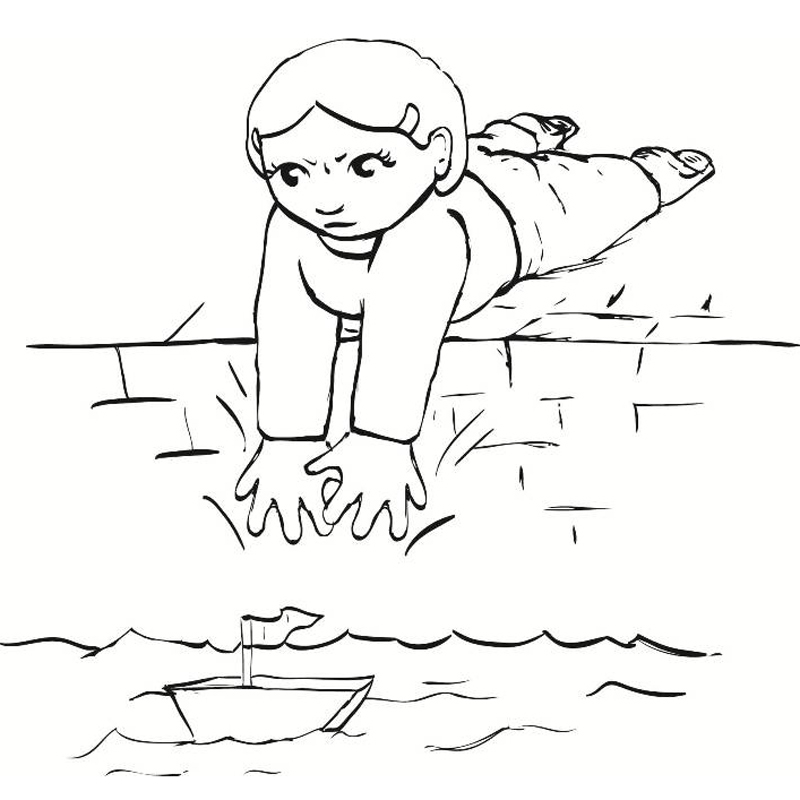

Supplement: Appendix S5 — All NeoHelp stimuli in JPEG format. All stimuli are provided as used in the study reported. (ZIP) [file pone.0084373.s005.zip › Stimuli/boat_girl.jpg]

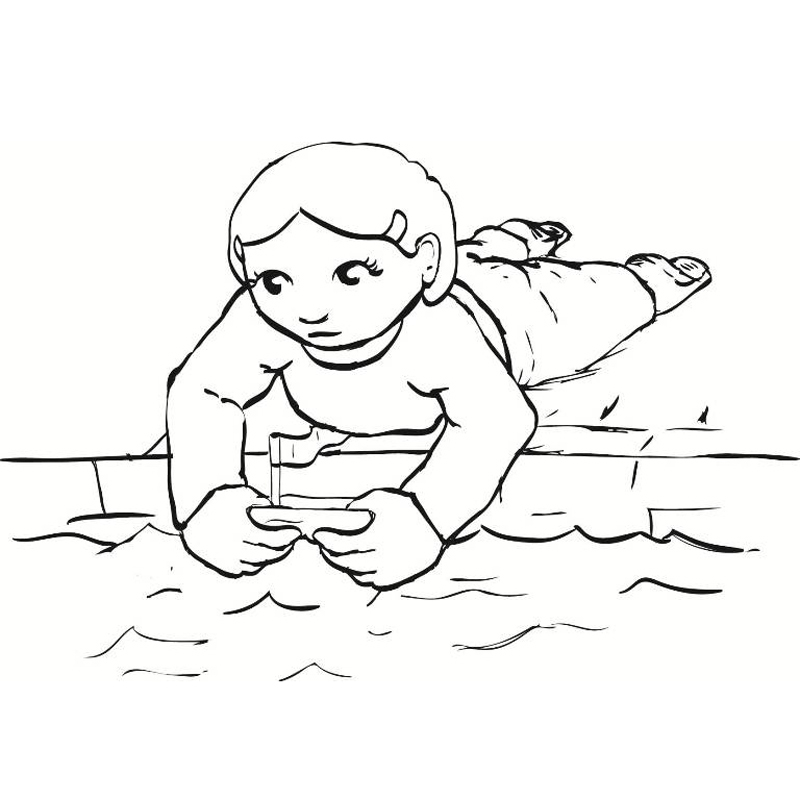

Supplement: Appendix S5 — All NeoHelp stimuli in JPEG format. All stimuli are provided as used in the study reported. (ZIP) [file pone.0084373.s005.zip › Stimuli/boat_girl_success.jpg]

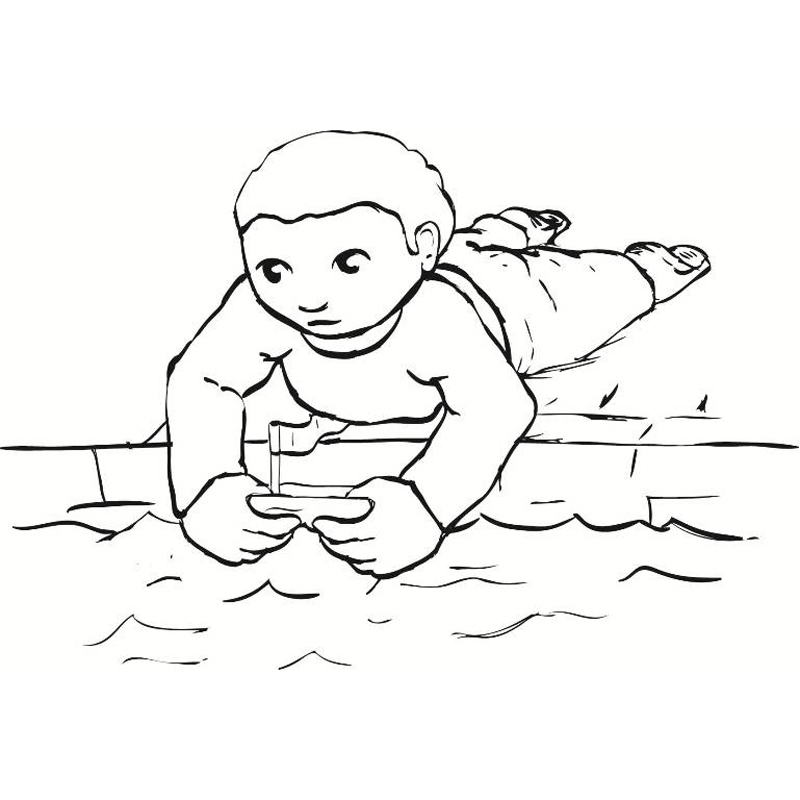

Supplement: Appendix S5 — All NeoHelp stimuli in JPEG format. All stimuli are provided as used in the study reported. (ZIP) [file pone.0084373.s005.zip › Stimuli/boat_success.jpg]

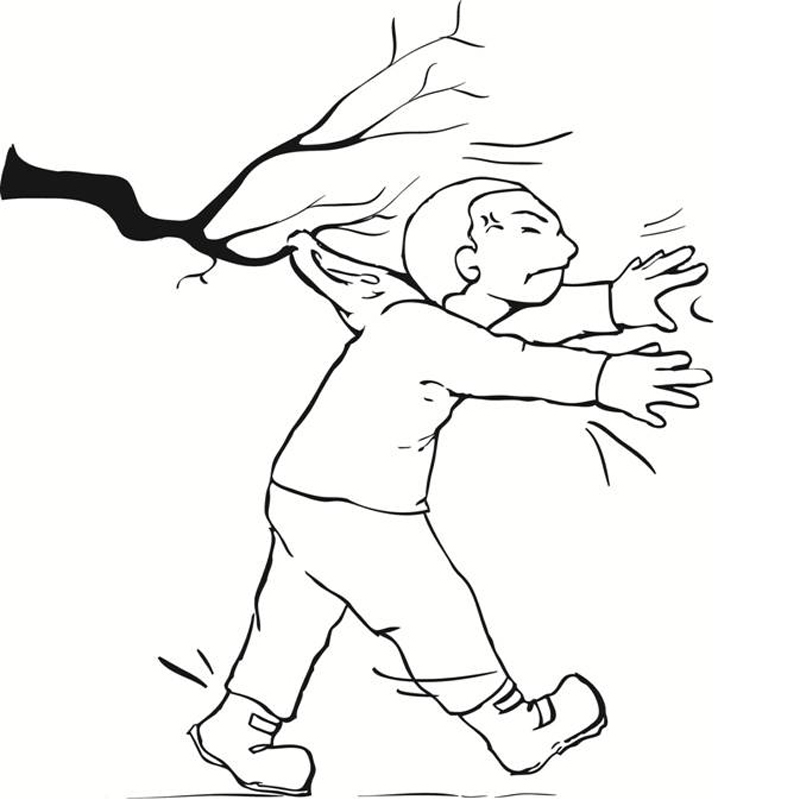

Supplement: Appendix S5 — All NeoHelp stimuli in JPEG format. All stimuli are provided as used in the study reported. (ZIP) [file pone.0084373.s005.zip › Stimuli/branch.jpg]

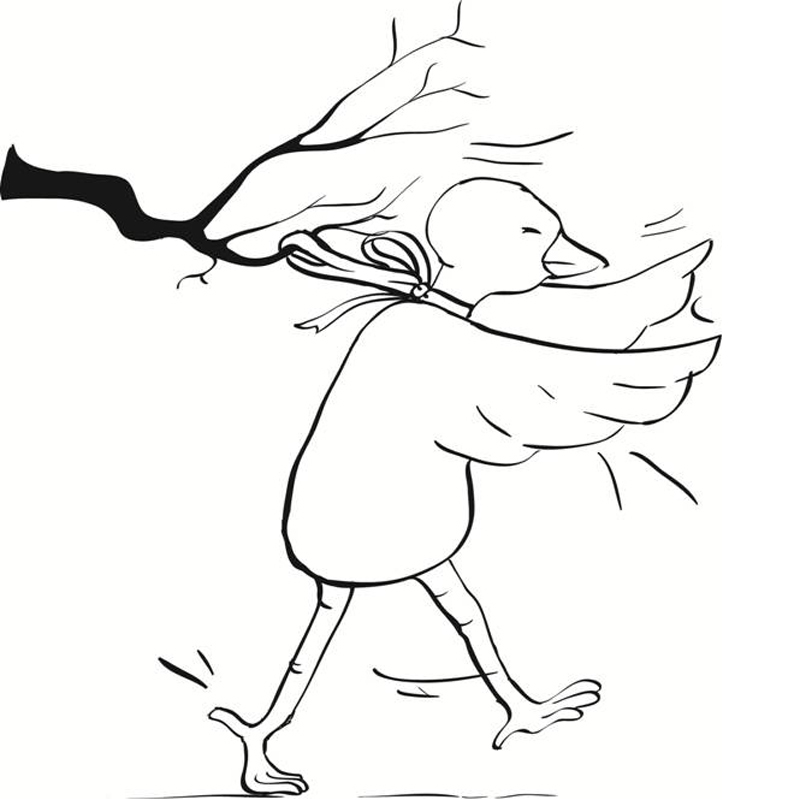

Supplement: Appendix S5 — All NeoHelp stimuli in JPEG format. All stimuli are provided as used in the study reported. (ZIP) [file pone.0084373.s005.zip › Stimuli/branch_bird.jpg]

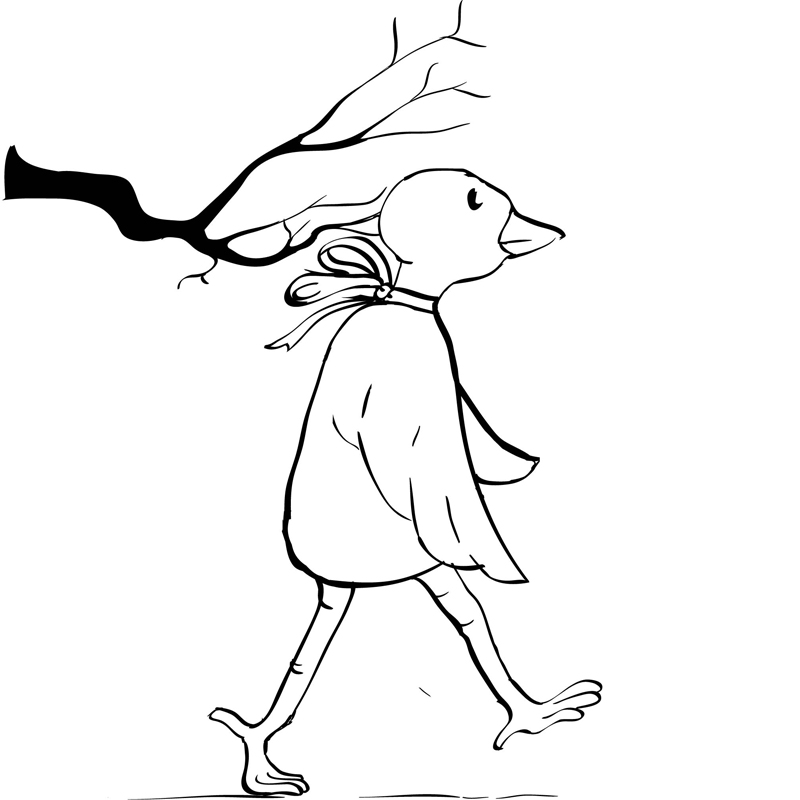

Supplement: Appendix S5 — All NeoHelp stimuli in JPEG format. All stimuli are provided as used in the study reported. (ZIP) [file pone.0084373.s005.zip › Stimuli/branch_bird_success.jpg]

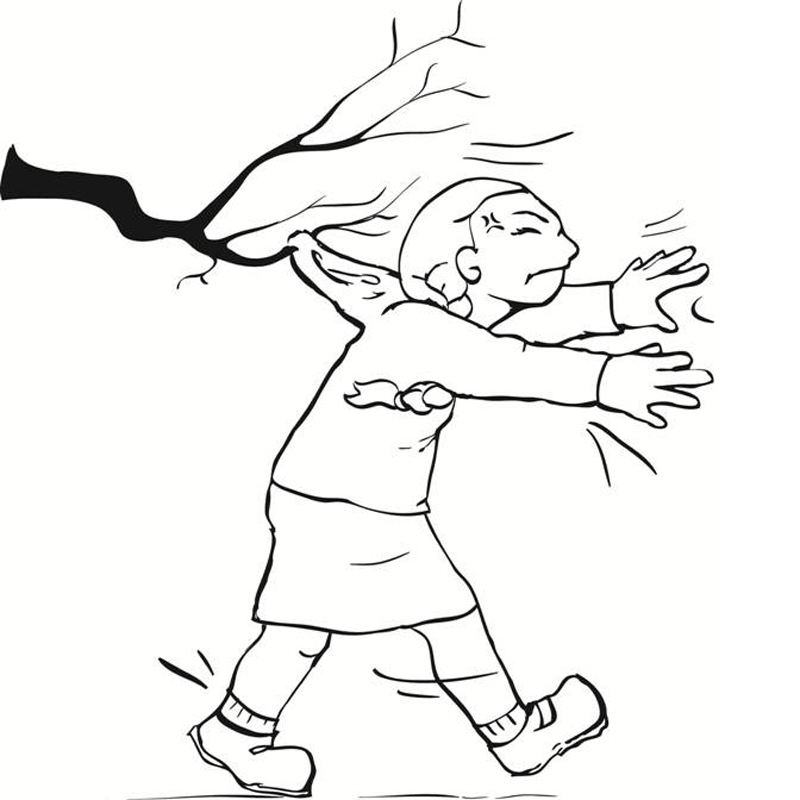

Supplement: Appendix S5 — All NeoHelp stimuli in JPEG format. All stimuli are provided as used in the study reported. (ZIP) [file pone.0084373.s005.zip › Stimuli/branch_girl.jpg]

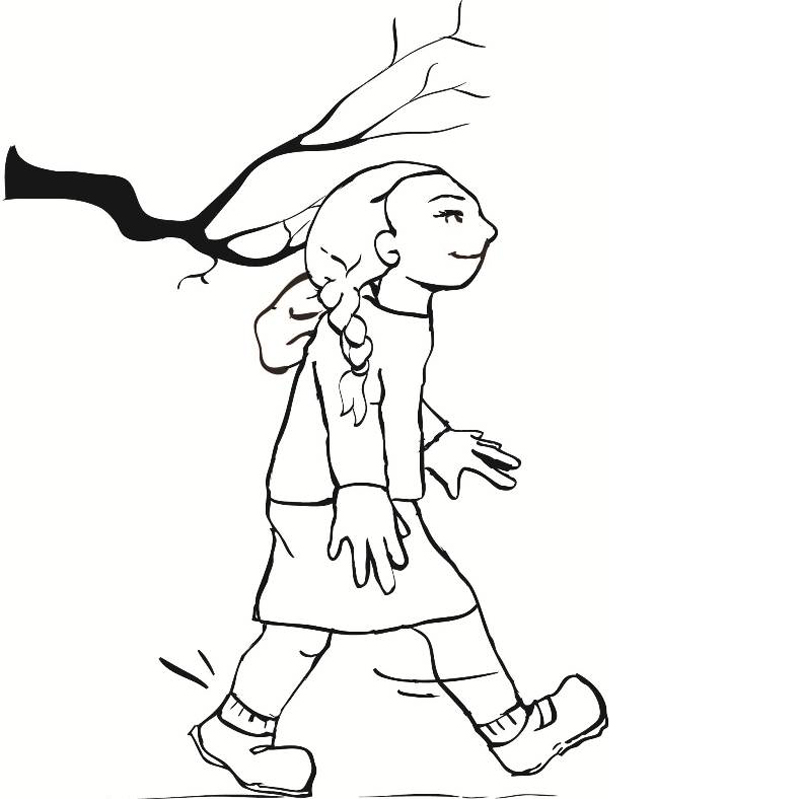

Supplement: Appendix S5 — All NeoHelp stimuli in JPEG format. All stimuli are provided as used in the study reported. (ZIP) [file pone.0084373.s005.zip › Stimuli/branch_girl_success.jpg]

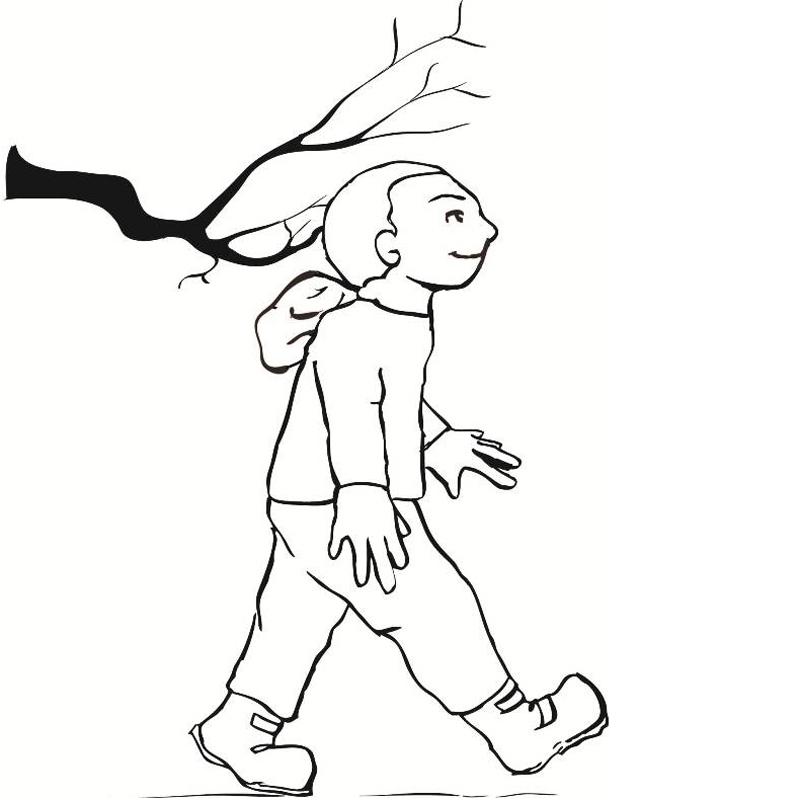

Supplement: Appendix S5 — All NeoHelp stimuli in JPEG format. All stimuli are provided as used in the study reported. (ZIP) [file pone.0084373.s005.zip › Stimuli/branch_success.jpg]

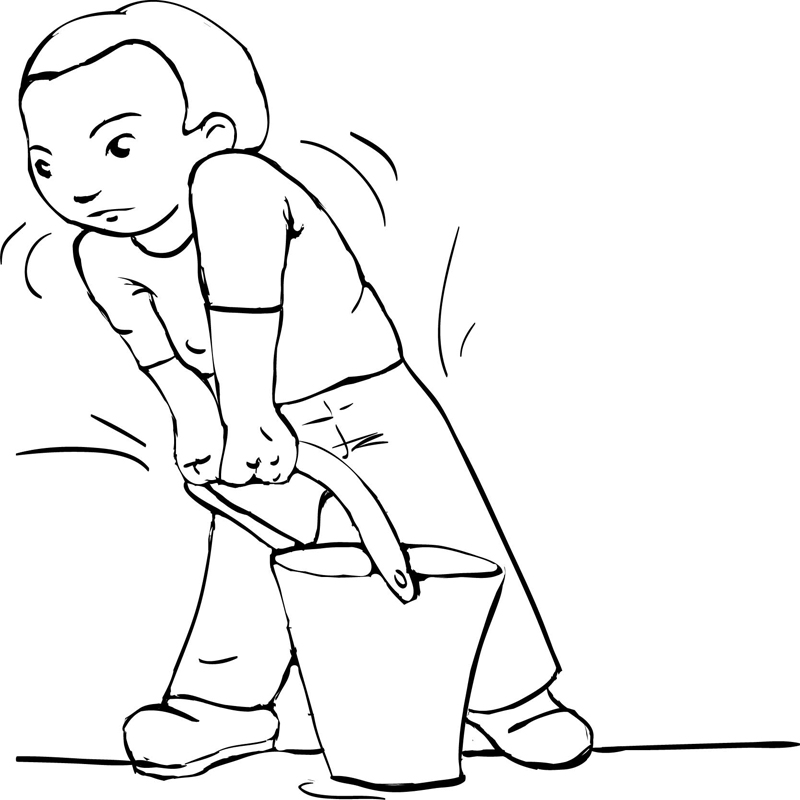

Supplement: Appendix S5 — All NeoHelp stimuli in JPEG format. All stimuli are provided as used in the study reported. (ZIP) [file pone.0084373.s005.zip › Stimuli/bucket.jpg]

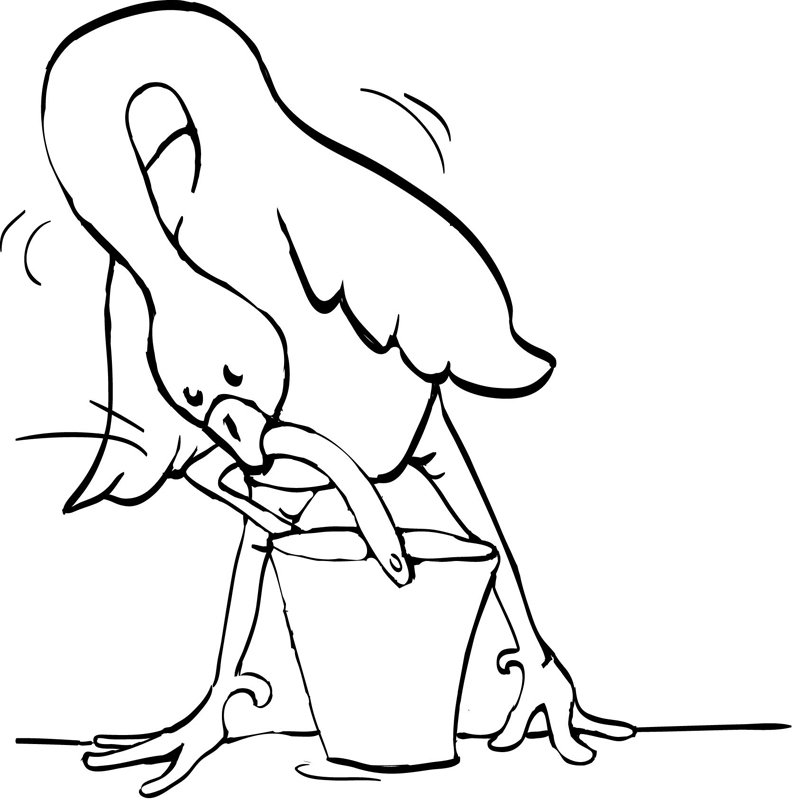

Supplement: Appendix S5 — All NeoHelp stimuli in JPEG format. All stimuli are provided as used in the study reported. (ZIP) [file pone.0084373.s005.zip › Stimuli/bucket_bird.jpg]

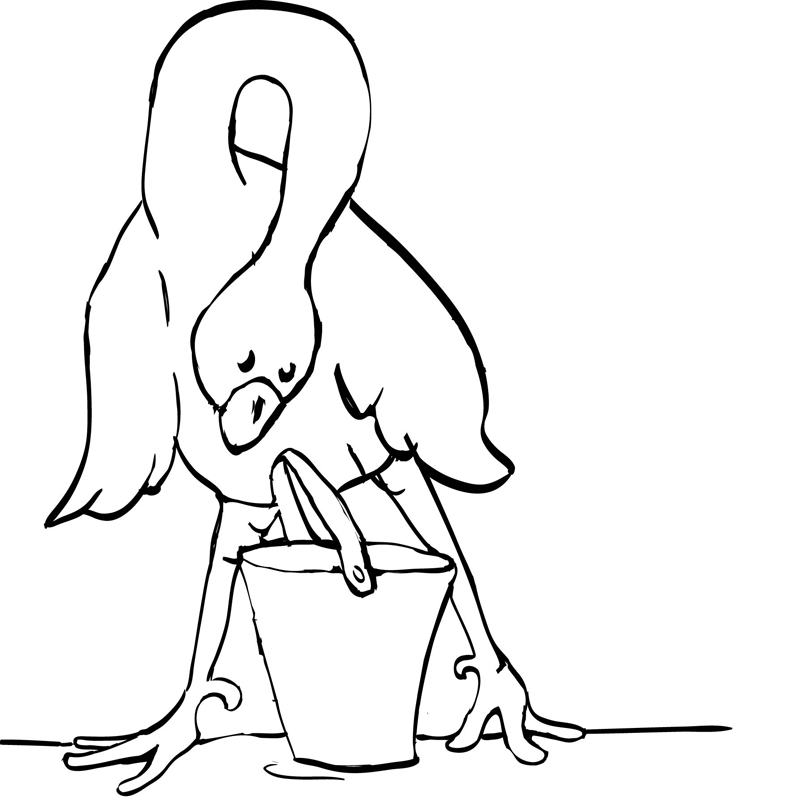

Supplement: Appendix S5 — All NeoHelp stimuli in JPEG format. All stimuli are provided as used in the study reported. (ZIP) [file pone.0084373.s005.zip › Stimuli/bucket_bird_success.jpg]

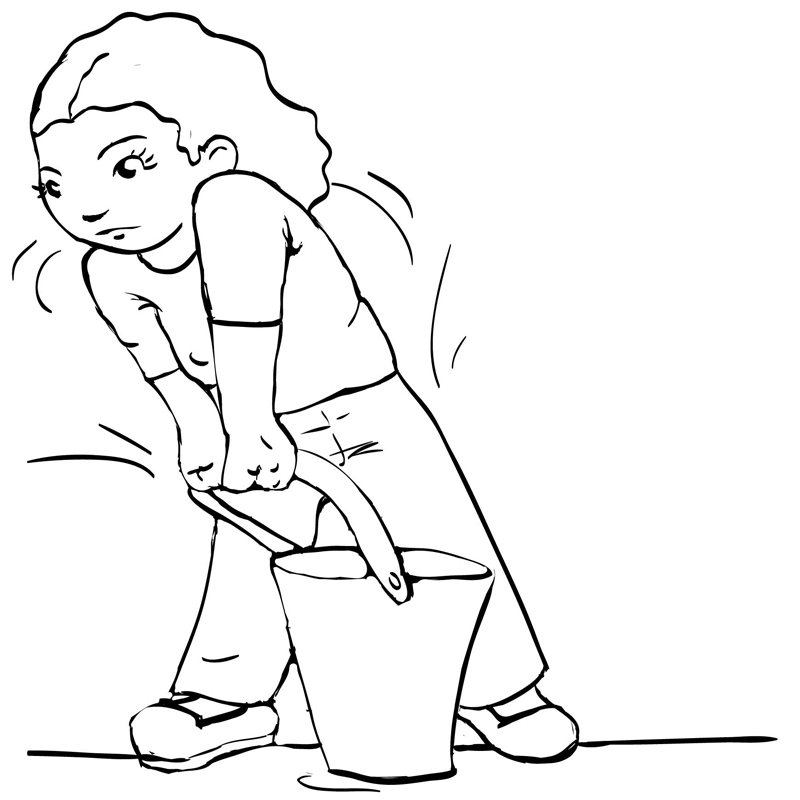

Supplement: Appendix S5 — All NeoHelp stimuli in JPEG format. All stimuli are provided as used in the study reported. (ZIP) [file pone.0084373.s005.zip › Stimuli/bucket_girl.jpg]

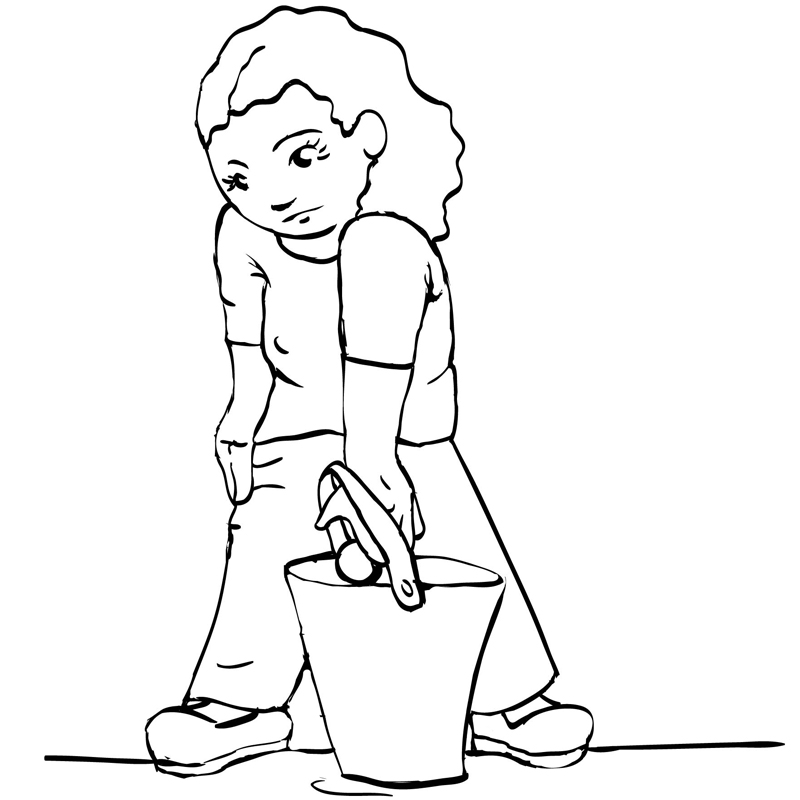

Supplement: Appendix S5 — All NeoHelp stimuli in JPEG format. All stimuli are provided as used in the study reported. (ZIP) [file pone.0084373.s005.zip › Stimuli/bucket_girl_success.jpg]

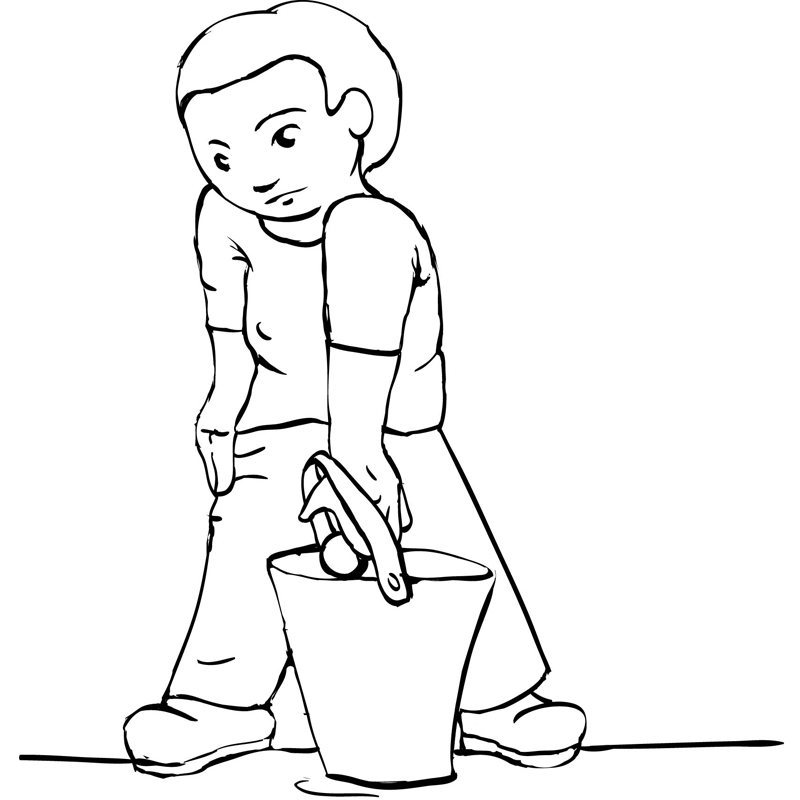

Supplement: Appendix S5 — All NeoHelp stimuli in JPEG format. All stimuli are provided as used in the study reported. (ZIP) [file pone.0084373.s005.zip › Stimuli/bucket_success.jpg]

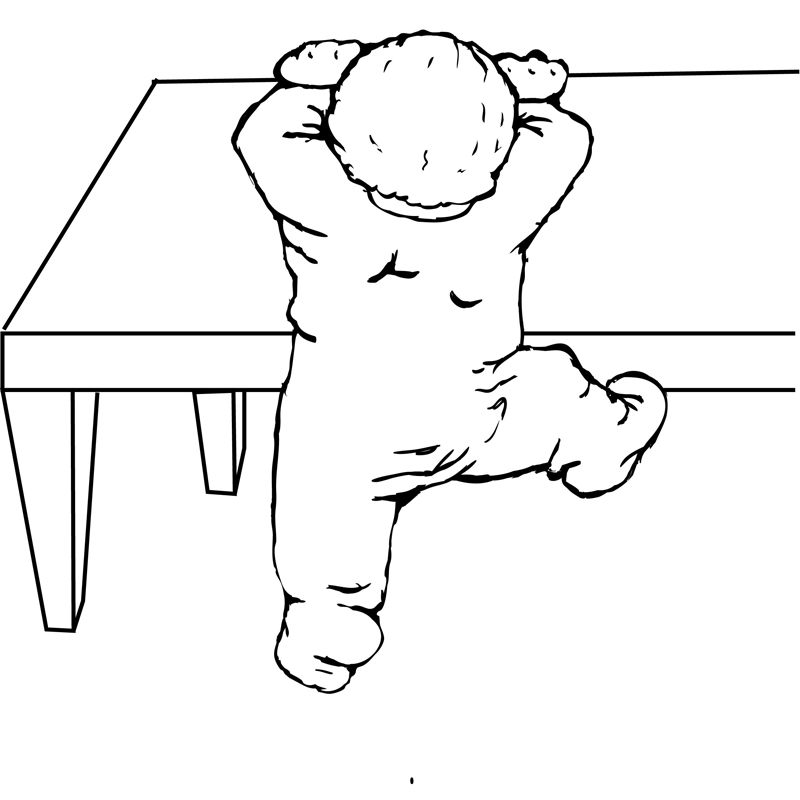

Supplement: Appendix S5 — All NeoHelp stimuli in JPEG format. All stimuli are provided as used in the study reported. (ZIP) [file pone.0084373.s005.zip › Stimuli/climb.jpg]

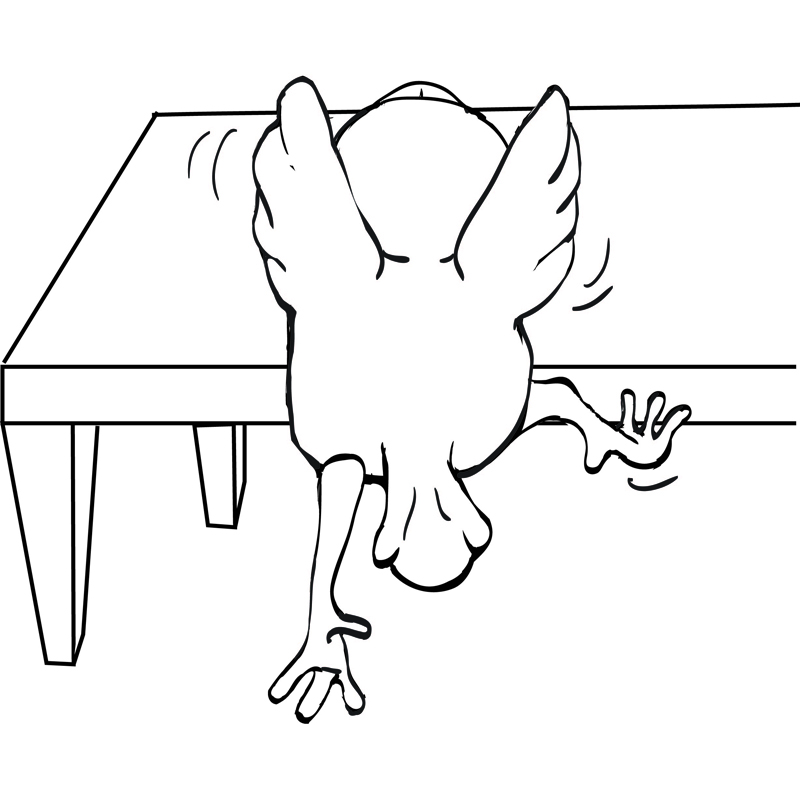

Supplement: Appendix S5 — All NeoHelp stimuli in JPEG format. All stimuli are provided as used in the study reported. (ZIP) [file pone.0084373.s005.zip › Stimuli/climb_bird.jpg]

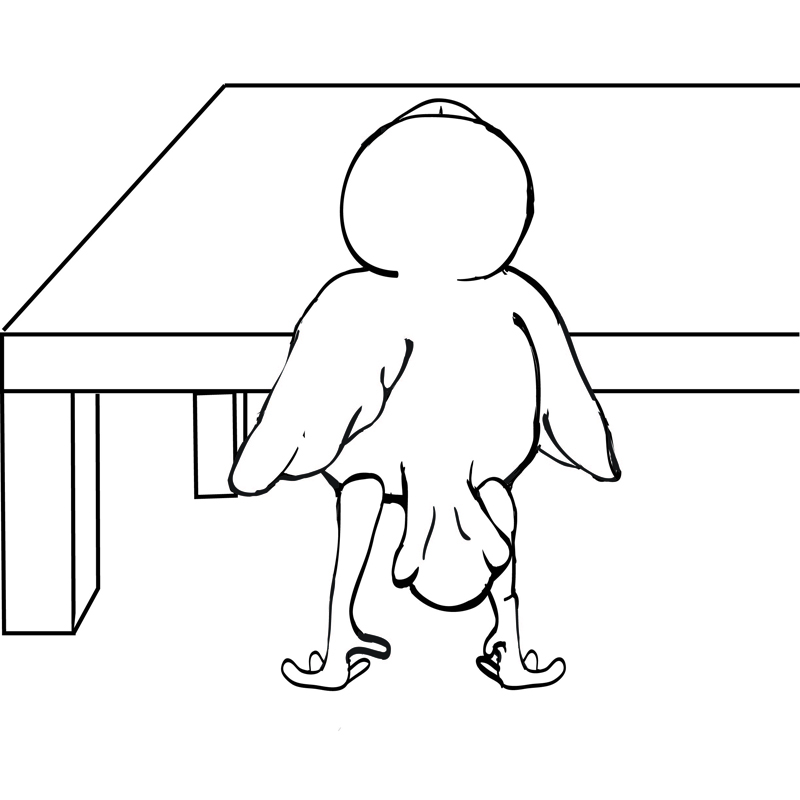

Supplement: Appendix S5 — All NeoHelp stimuli in JPEG format. All stimuli are provided as used in the study reported. (ZIP) [file pone.0084373.s005.zip › Stimuli/climb_bird_success.jpg]

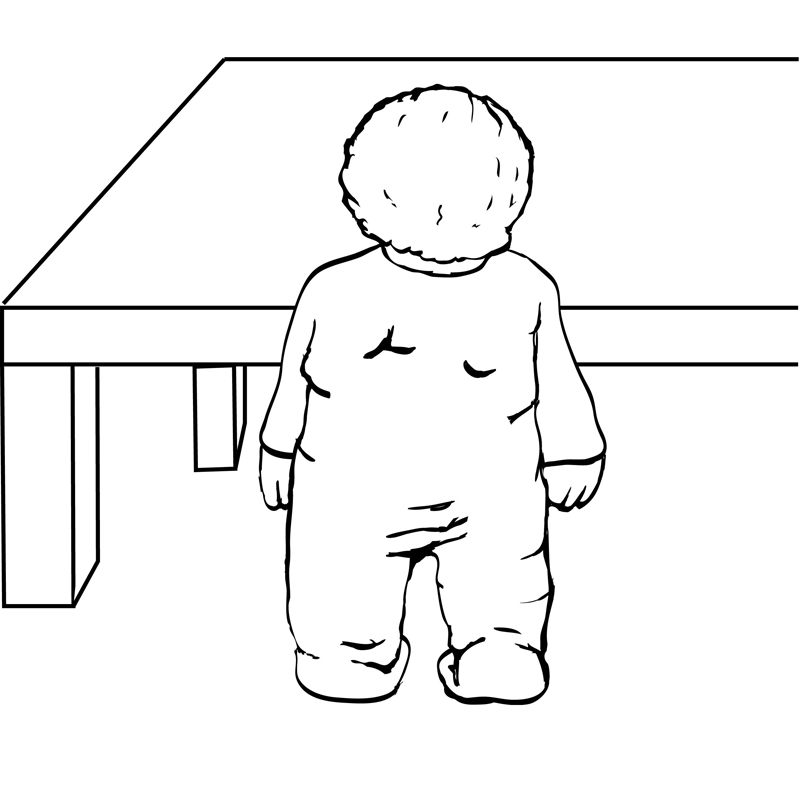

Supplement: Appendix S5 — All NeoHelp stimuli in JPEG format. All stimuli are provided as used in the study reported. (ZIP) [file pone.0084373.s005.zip › Stimuli/climb_success.jpg]

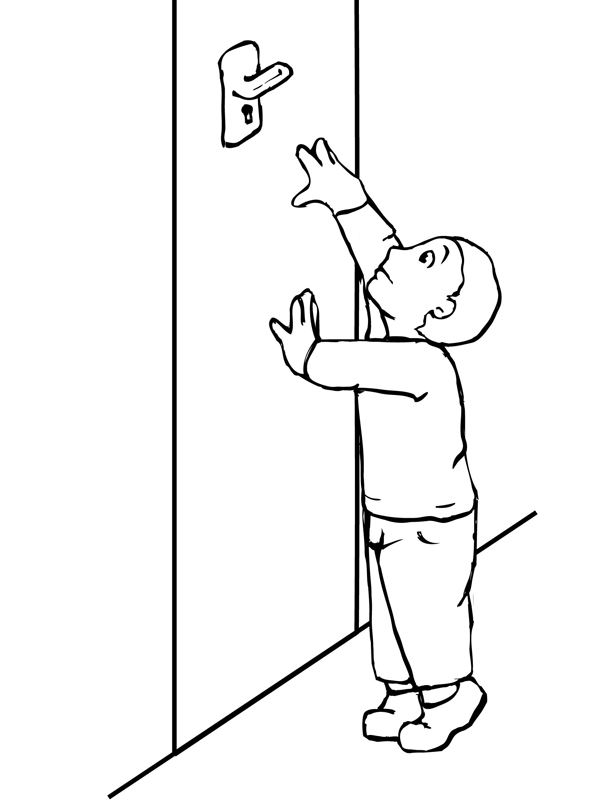

Supplement: Appendix S5 — All NeoHelp stimuli in JPEG format. All stimuli are provided as used in the study reported. (ZIP) [file pone.0084373.s005.zip › Stimuli/door.jpg]

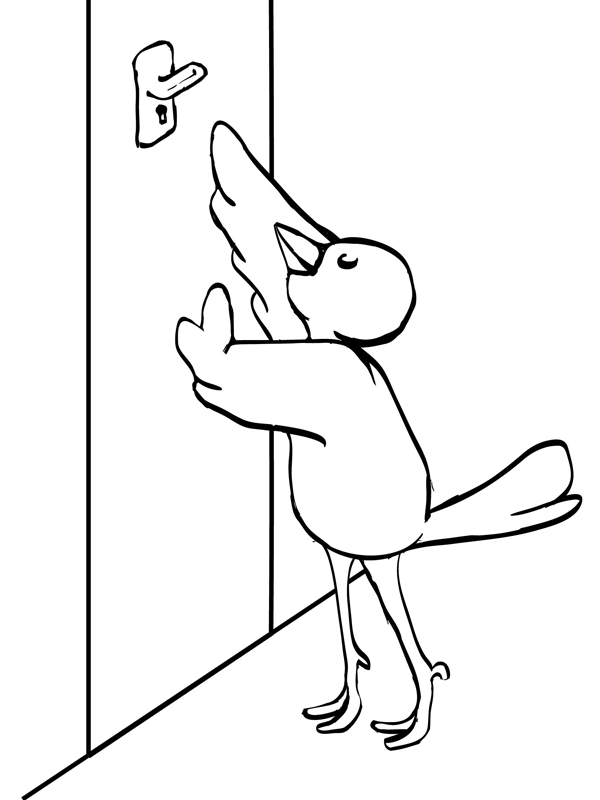

Supplement: Appendix S5 — All NeoHelp stimuli in JPEG format. All stimuli are provided as used in the study reported. (ZIP) [file pone.0084373.s005.zip › Stimuli/door_bird.jpg]

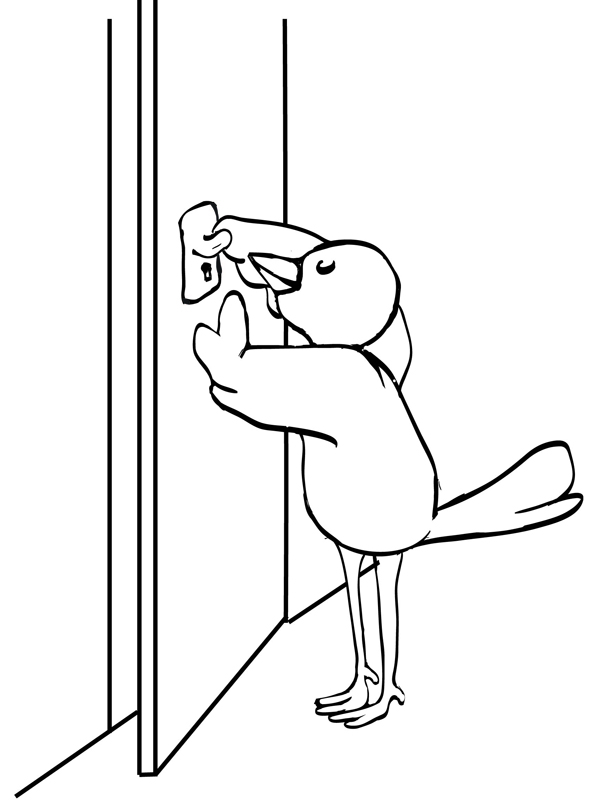

Supplement: Appendix S5 — All NeoHelp stimuli in JPEG format. All stimuli are provided as used in the study reported. (ZIP) [file pone.0084373.s005.zip › Stimuli/door_bird_success.jpg]

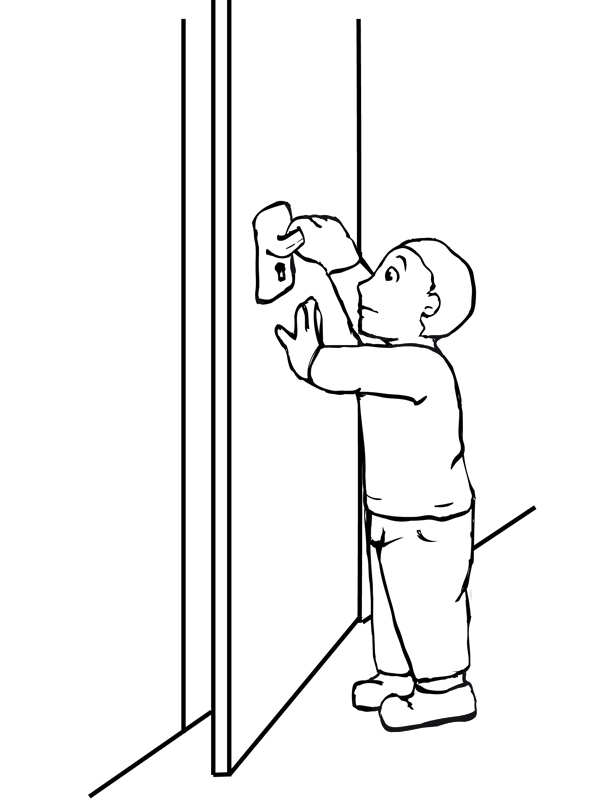

Supplement: Appendix S5 — All NeoHelp stimuli in JPEG format. All stimuli are provided as used in the study reported. (ZIP) [file pone.0084373.s005.zip › Stimuli/door_success.jpg]

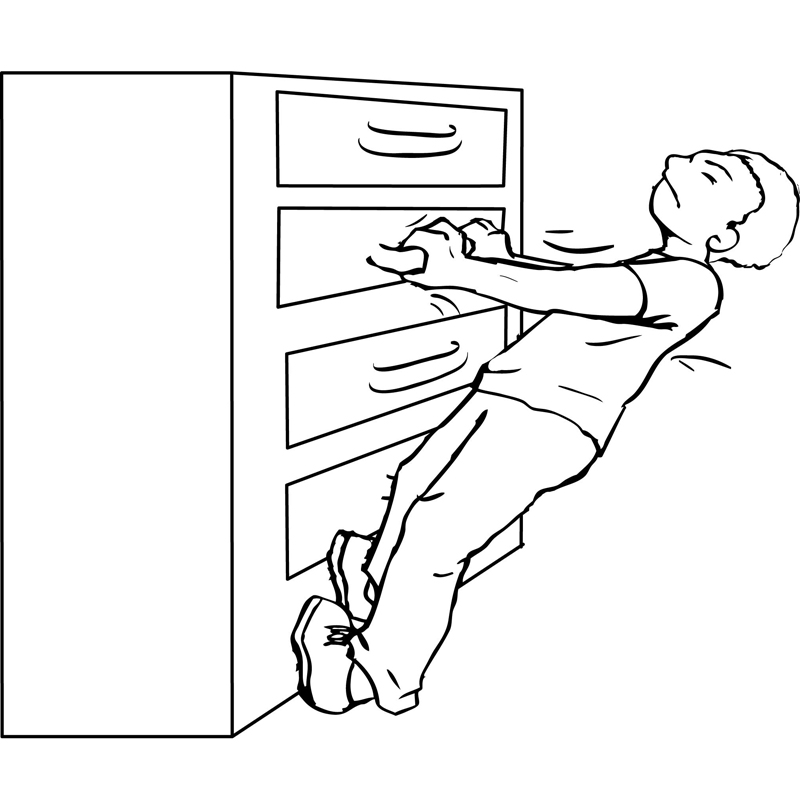

Supplement: Appendix S5 — All NeoHelp stimuli in JPEG format. All stimuli are provided as used in the study reported. (ZIP) [file pone.0084373.s005.zip › Stimuli/drawer.jpg]

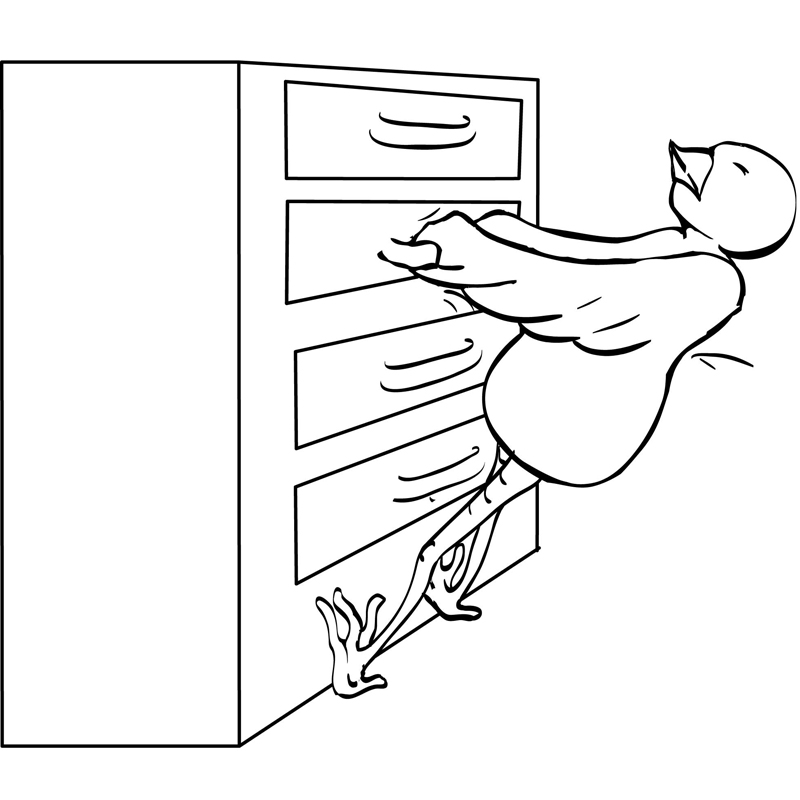

Supplement: Appendix S5 — All NeoHelp stimuli in JPEG format. All stimuli are provided as used in the study reported. (ZIP) [file pone.0084373.s005.zip › Stimuli/drawer_bird.jpg]

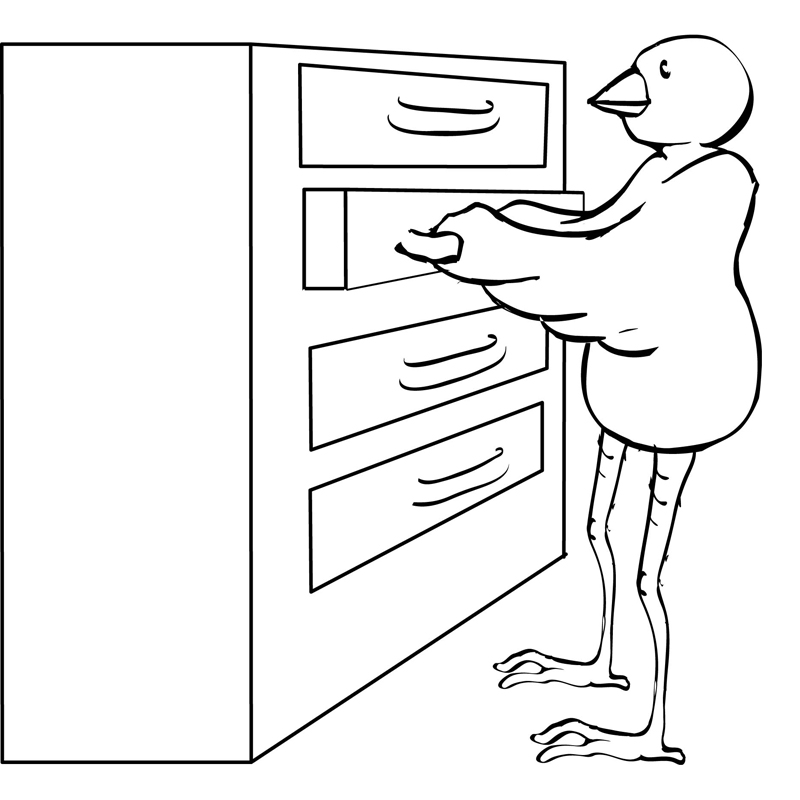

Supplement: Appendix S5 — All NeoHelp stimuli in JPEG format. All stimuli are provided as used in the study reported. (ZIP) [file pone.0084373.s005.zip › Stimuli/drawer_bird_success.jpg]

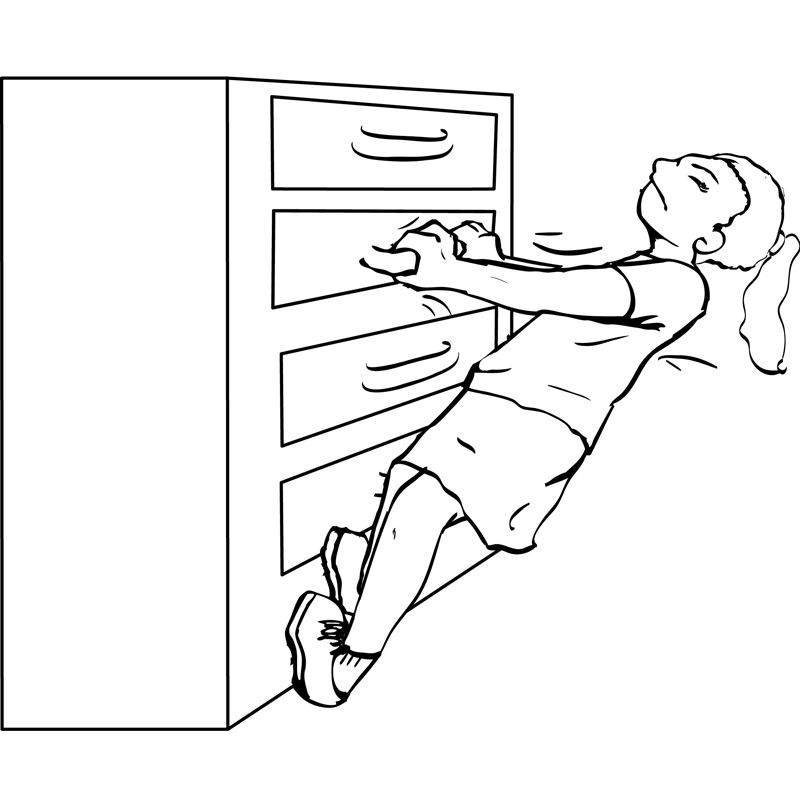

Supplement: Appendix S5 — All NeoHelp stimuli in JPEG format. All stimuli are provided as used in the study reported. (ZIP) [file pone.0084373.s005.zip › Stimuli/drawer_girl.jpg]

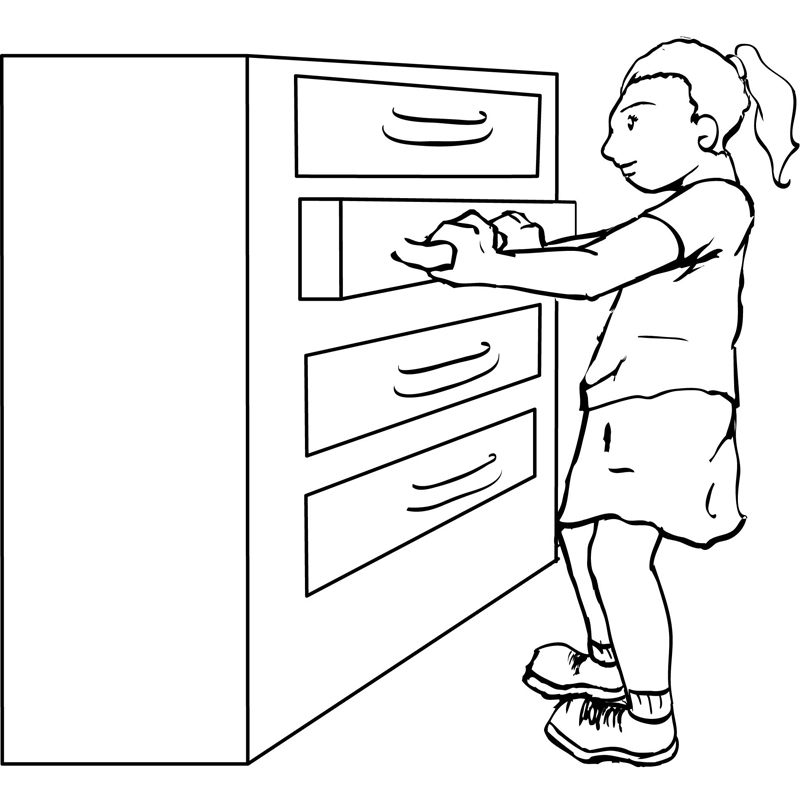

Supplement: Appendix S5 — All NeoHelp stimuli in JPEG format. All stimuli are provided as used in the study reported. (ZIP) [file pone.0084373.s005.zip › Stimuli/drawer_girl_success.jpg]

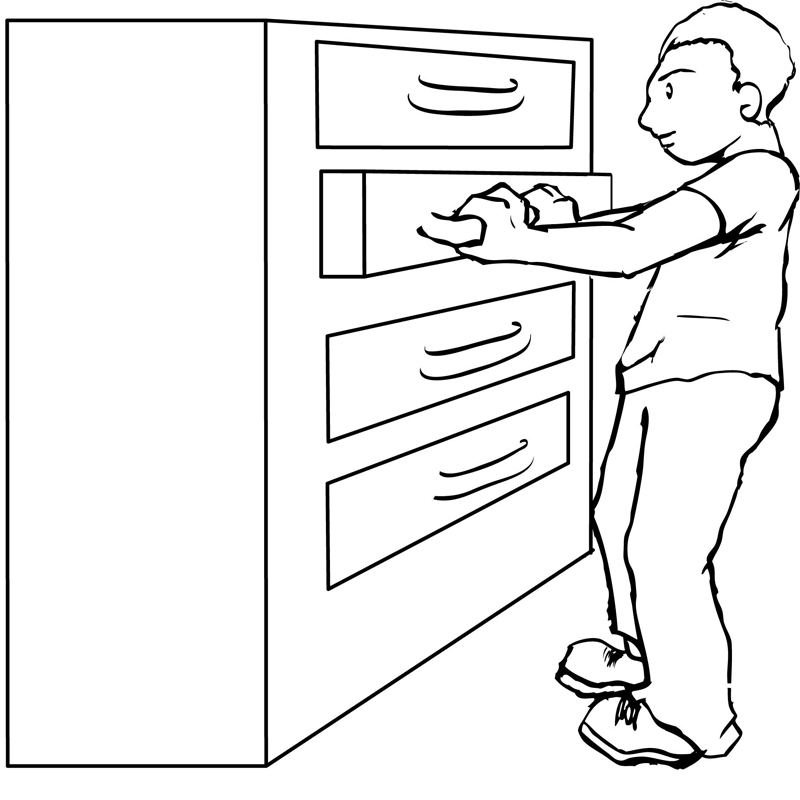

Supplement: Appendix S5 — All NeoHelp stimuli in JPEG format. All stimuli are provided as used in the study reported. (ZIP) [file pone.0084373.s005.zip › Stimuli/drawer_success.jpg]

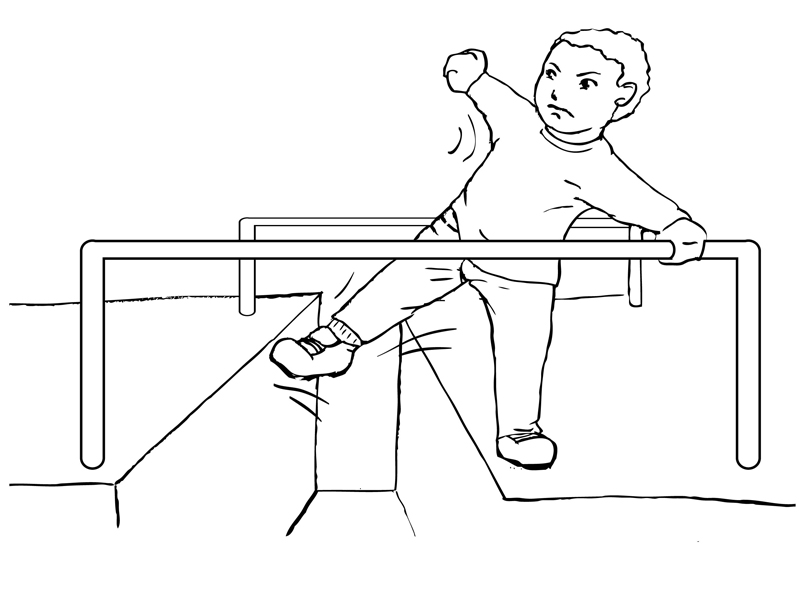

Supplement: Appendix S5 — All NeoHelp stimuli in JPEG format. All stimuli are provided as used in the study reported. (ZIP) [file pone.0084373.s005.zip › Stimuli/gap.jpg]

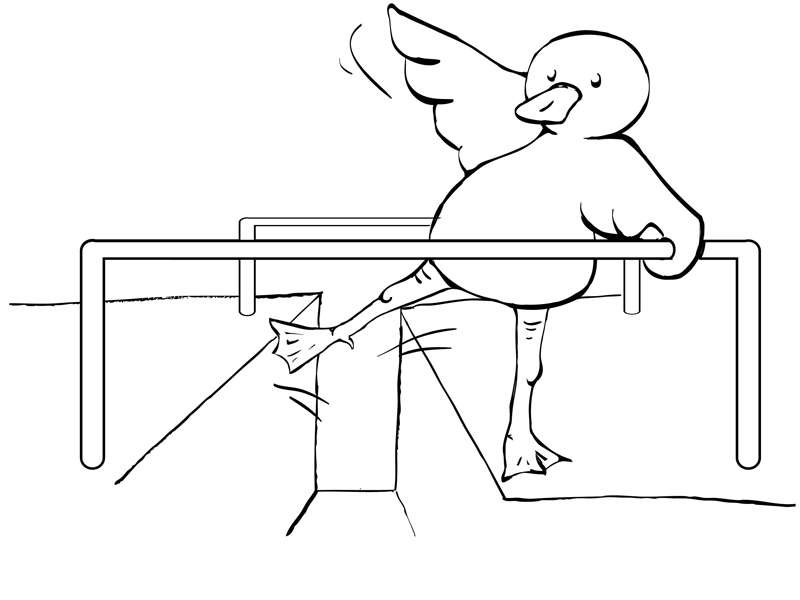

Supplement: Appendix S5 — All NeoHelp stimuli in JPEG format. All stimuli are provided as used in the study reported. (ZIP) [file pone.0084373.s005.zip › Stimuli/gap_bird.jpg]

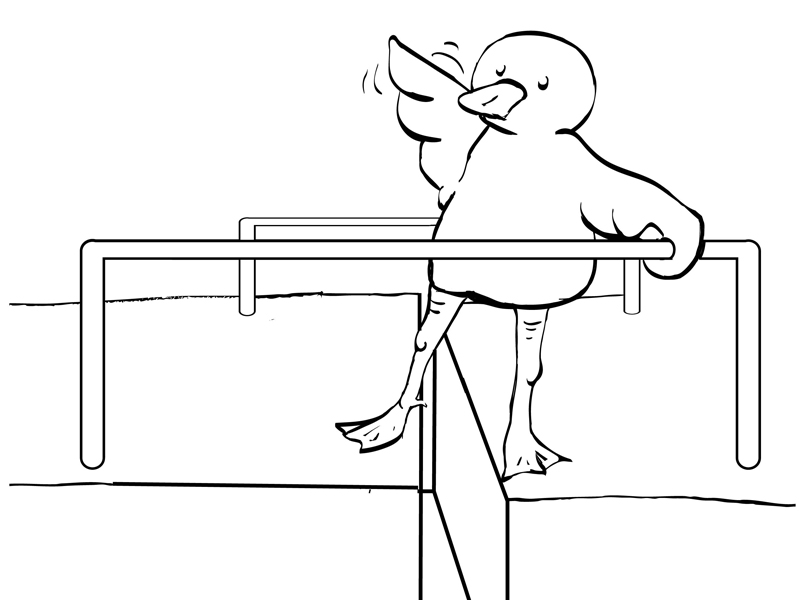

Supplement: Appendix S5 — All NeoHelp stimuli in JPEG format. All stimuli are provided as used in the study reported. (ZIP) [file pone.0084373.s005.zip › Stimuli/gap_bird_success.jpg]

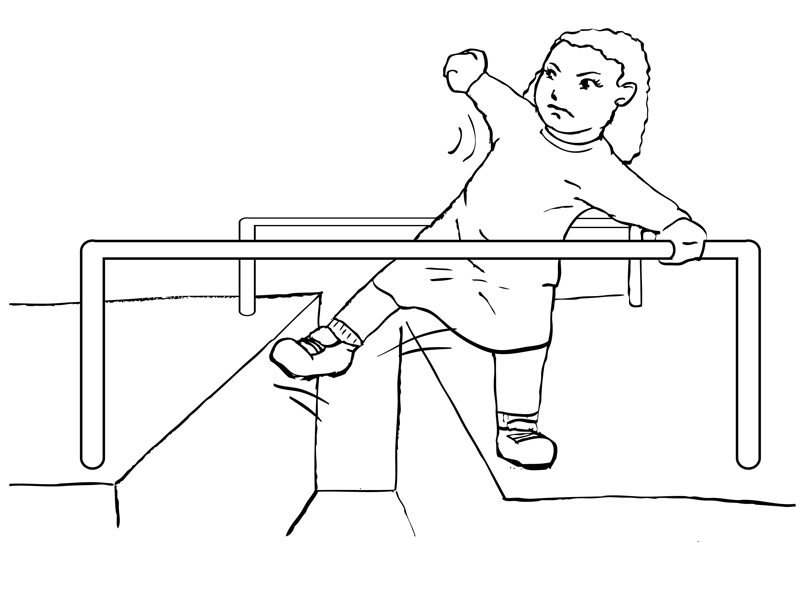

Supplement: Appendix S5 — All NeoHelp stimuli in JPEG format. All stimuli are provided as used in the study reported. (ZIP) [file pone.0084373.s005.zip › Stimuli/gap_girl.jpg]

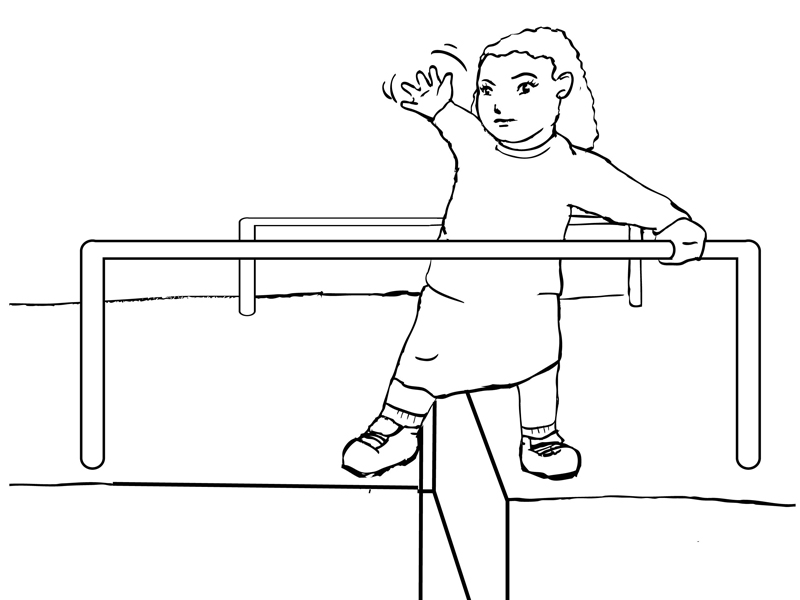

Supplement: Appendix S5 — All NeoHelp stimuli in JPEG format. All stimuli are provided as used in the study reported. (ZIP) [file pone.0084373.s005.zip › Stimuli/gap_girl_success.jpg]

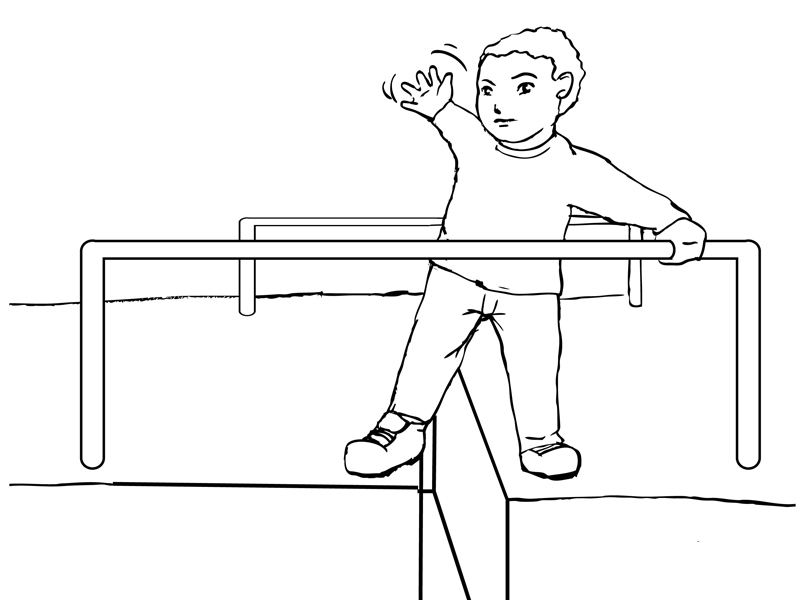

Supplement: Appendix S5 — All NeoHelp stimuli in JPEG format. All stimuli are provided as used in the study reported. (ZIP) [file pone.0084373.s005.zip › Stimuli/gap_success.jpg]

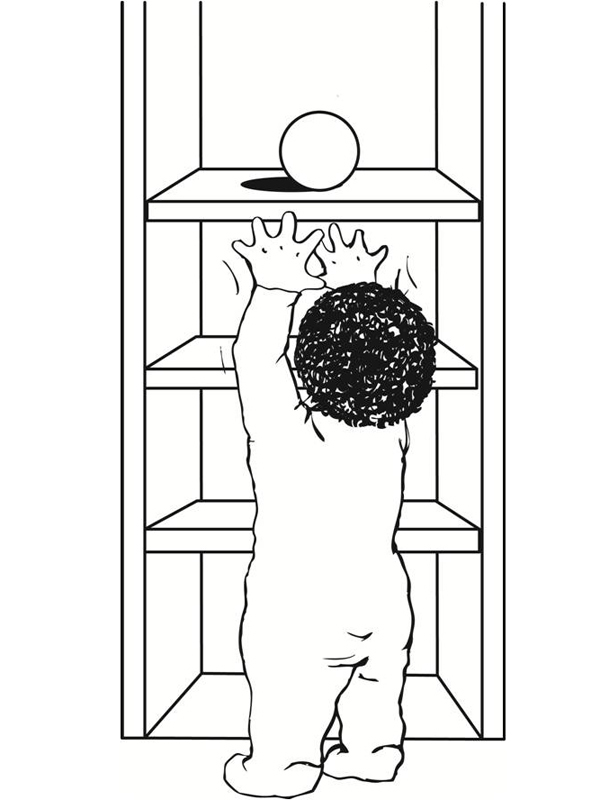

Supplement: Appendix S5 — All NeoHelp stimuli in JPEG format. All stimuli are provided as used in the study reported. (ZIP) [file pone.0084373.s005.zip › Stimuli/shelf.jpg]

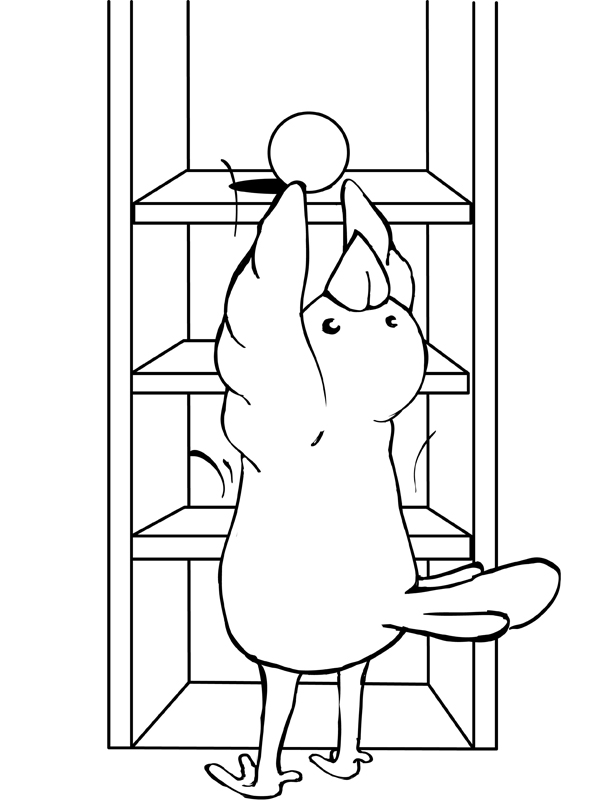

Supplement: Appendix S5 — All NeoHelp stimuli in JPEG format. All stimuli are provided as used in the study reported. (ZIP) [file pone.0084373.s005.zip › Stimuli/shelf_bird.jpg]

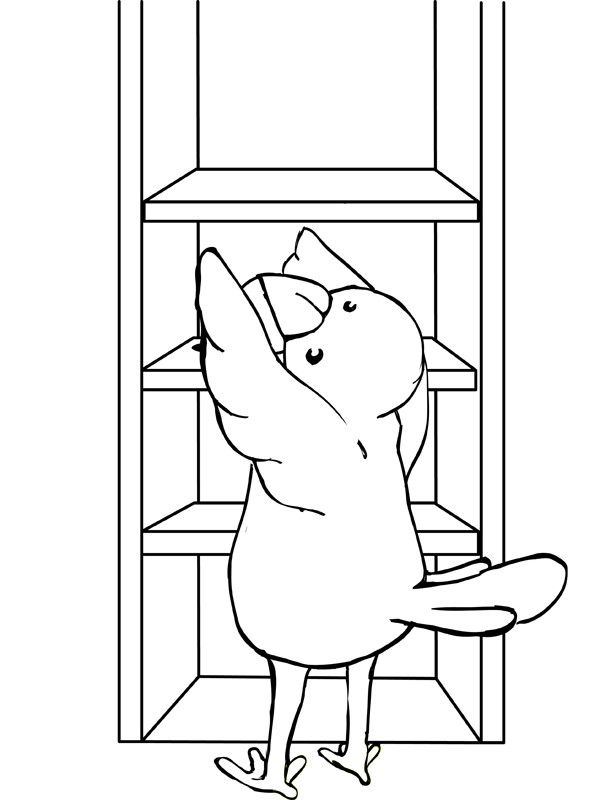

Supplement: Appendix S5 — All NeoHelp stimuli in JPEG format. All stimuli are provided as used in the study reported. (ZIP) [file pone.0084373.s005.zip › Stimuli/shelf_bird_success.jpg]

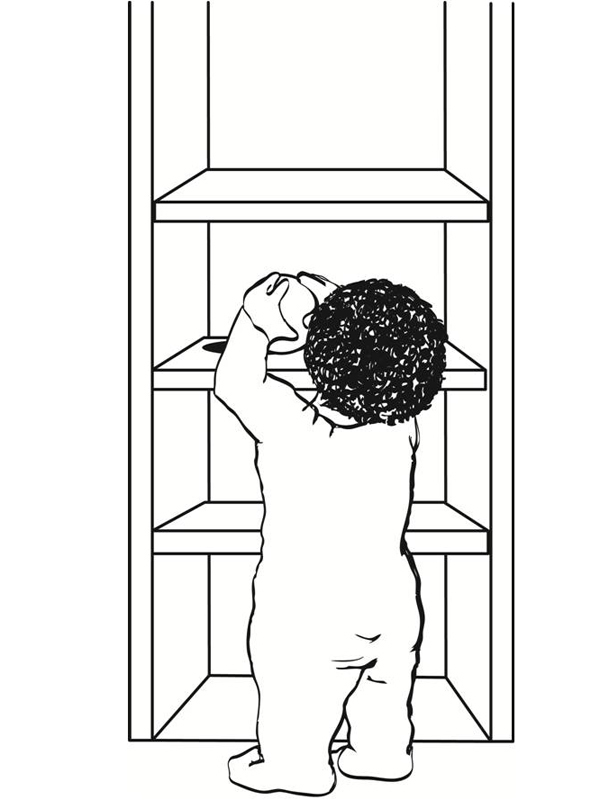

Supplement: Appendix S5 — All NeoHelp stimuli in JPEG format. All stimuli are provided as used in the study reported. (ZIP) [file pone.0084373.s005.zip › Stimuli/shelf_success.jpg]

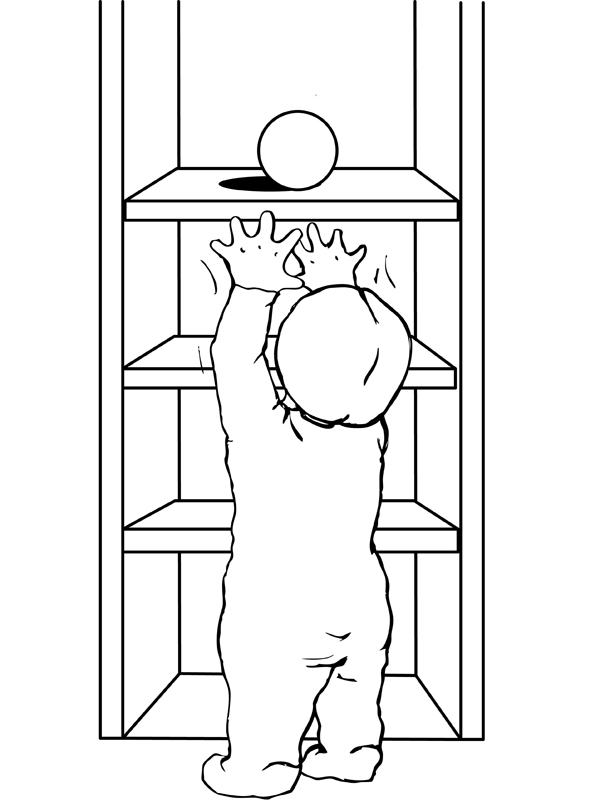

Supplement: Appendix S5 — All NeoHelp stimuli in JPEG format. All stimuli are provided as used in the study reported. (ZIP) [file pone.0084373.s005.zip › Stimuli/shelf_white.jpg]

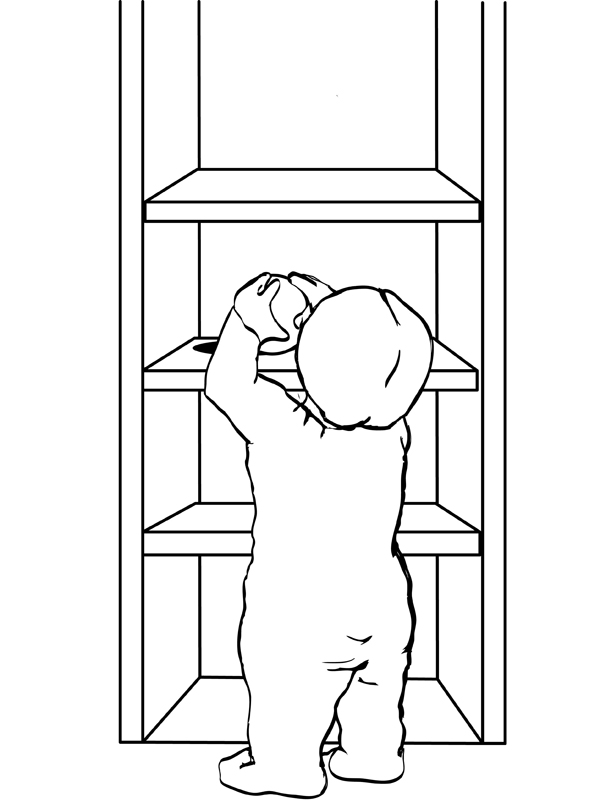

Supplement: Appendix S5 — All NeoHelp stimuli in JPEG format. All stimuli are provided as used in the study reported. (ZIP) [file pone.0084373.s005.zip › Stimuli/shelf_white_success.jpg]

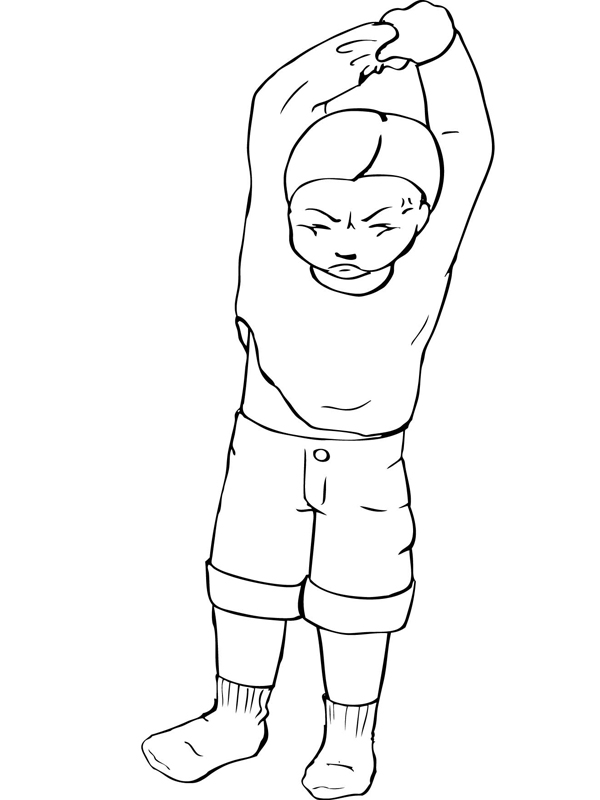

Supplement: Appendix S5 — All NeoHelp stimuli in JPEG format. All stimuli are provided as used in the study reported. (ZIP) [file pone.0084373.s005.zip › Stimuli/shirt.jpg]

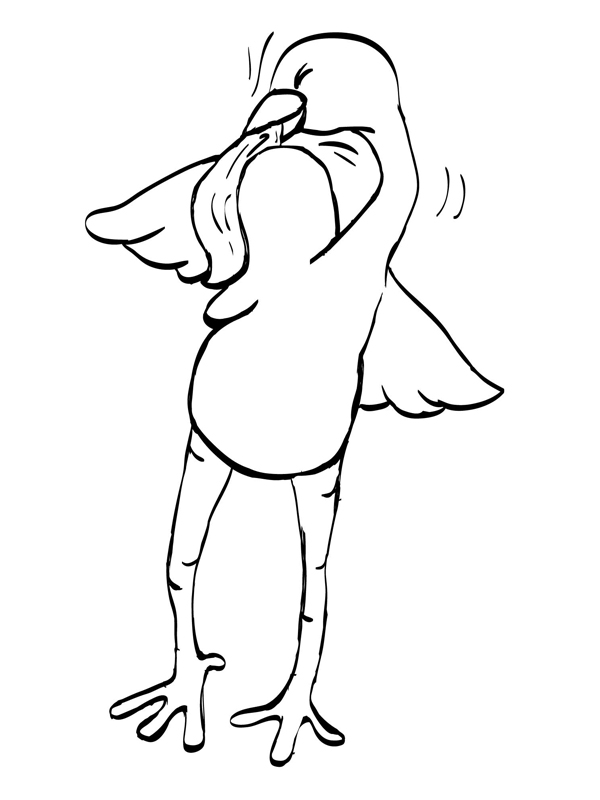

Supplement: Appendix S5 — All NeoHelp stimuli in JPEG format. All stimuli are provided as used in the study reported. (ZIP) [file pone.0084373.s005.zip › Stimuli/shirt_bird.jpg]

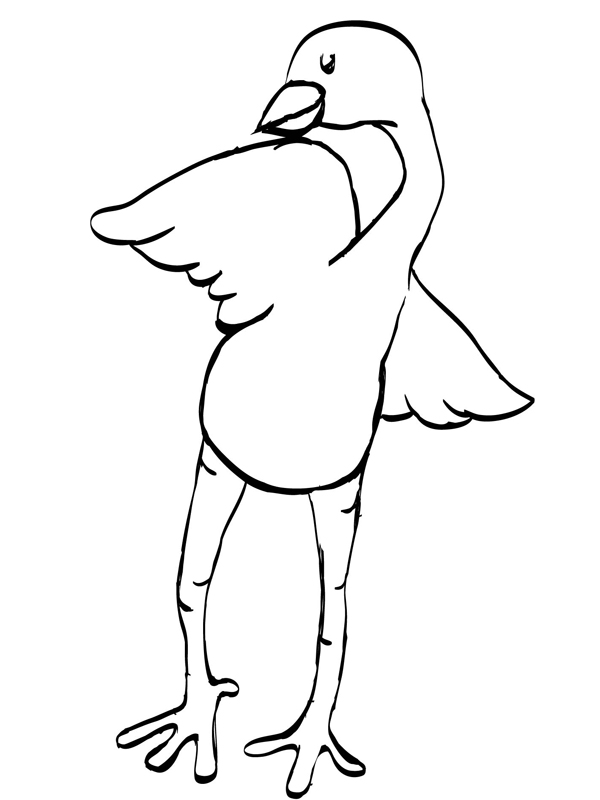

Supplement: Appendix S5 — All NeoHelp stimuli in JPEG format. All stimuli are provided as used in the study reported. (ZIP) [file pone.0084373.s005.zip › Stimuli/shirt_bird_success.jpg]

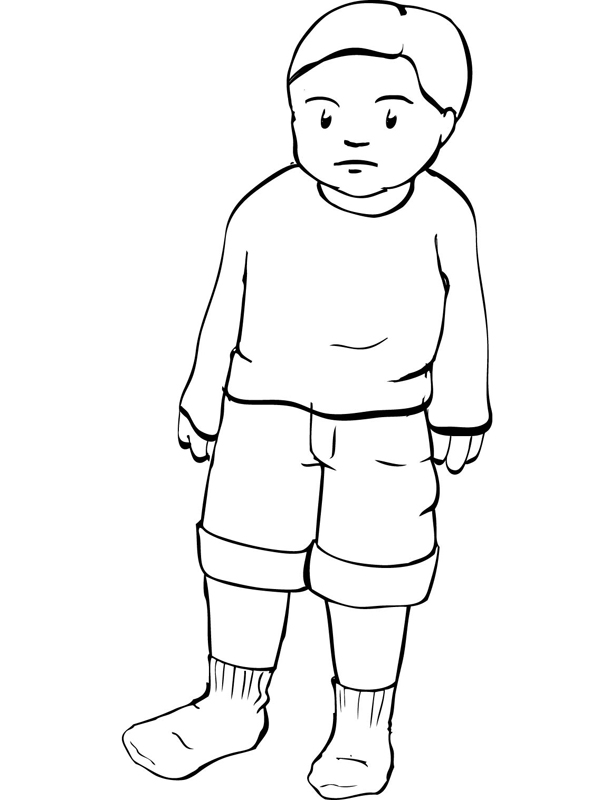

Supplement: Appendix S5 — All NeoHelp stimuli in JPEG format. All stimuli are provided as used in the study reported. (ZIP) [file pone.0084373.s005.zip › Stimuli/shirt_success.jpg]

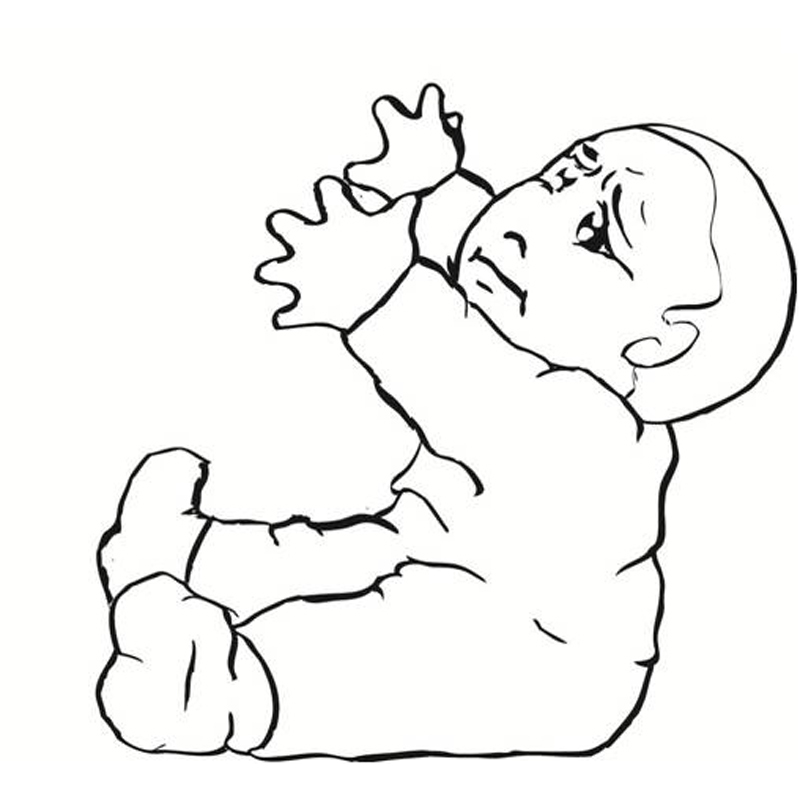

Supplement: Appendix S5 — All NeoHelp stimuli in JPEG format. All stimuli are provided as used in the study reported. (ZIP) [file pone.0084373.s005.zip › Stimuli/sit.jpg]

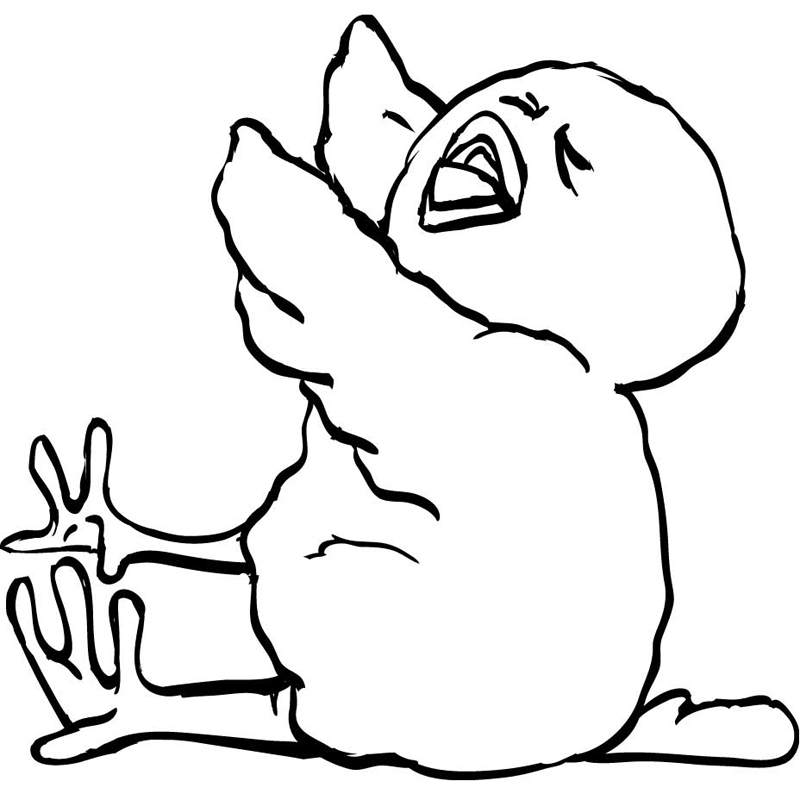

Supplement: Appendix S5 — All NeoHelp stimuli in JPEG format. All stimuli are provided as used in the study reported. (ZIP) [file pone.0084373.s005.zip › Stimuli/sit_bird.jpg]

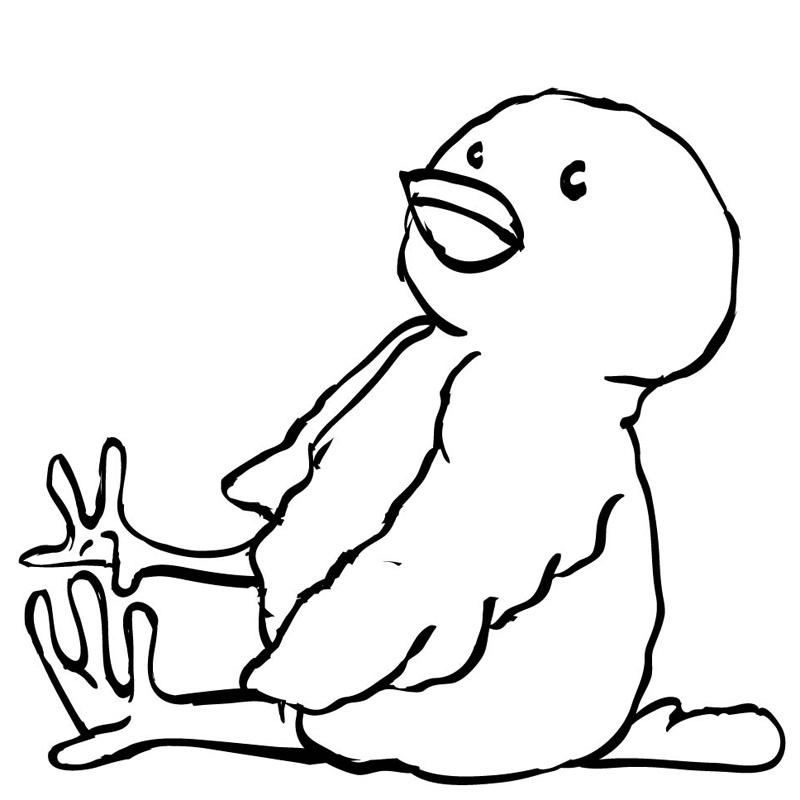

Supplement: Appendix S5 — All NeoHelp stimuli in JPEG format. All stimuli are provided as used in the study reported. (ZIP) [file pone.0084373.s005.zip › Stimuli/sit_bird_success.jpg]

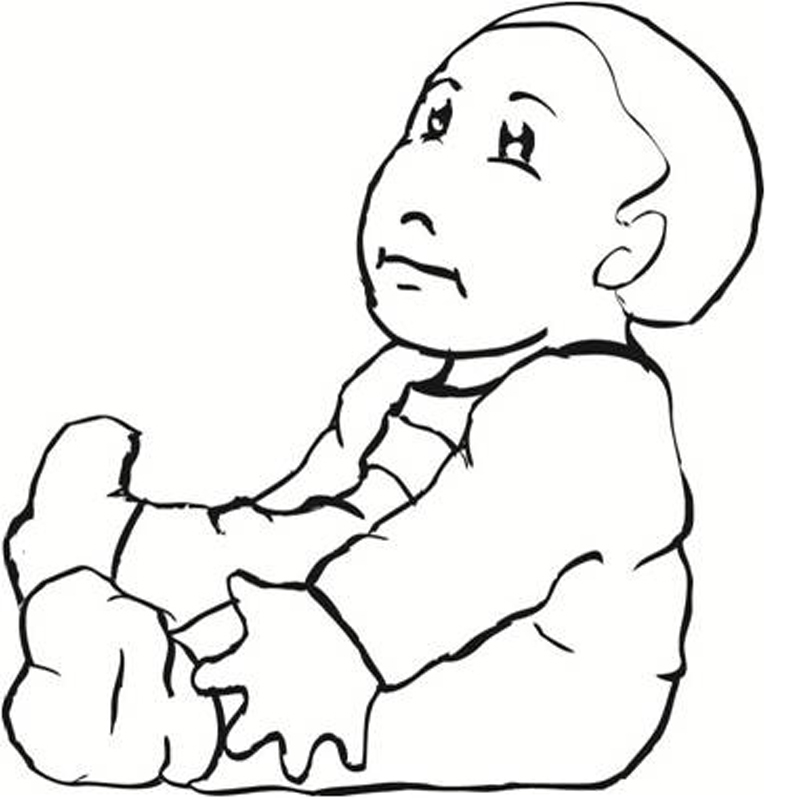

Supplement: Appendix S5 — All NeoHelp stimuli in JPEG format. All stimuli are provided as used in the study reported. (ZIP) [file pone.0084373.s005.zip › Stimuli/sit_success.jpg]

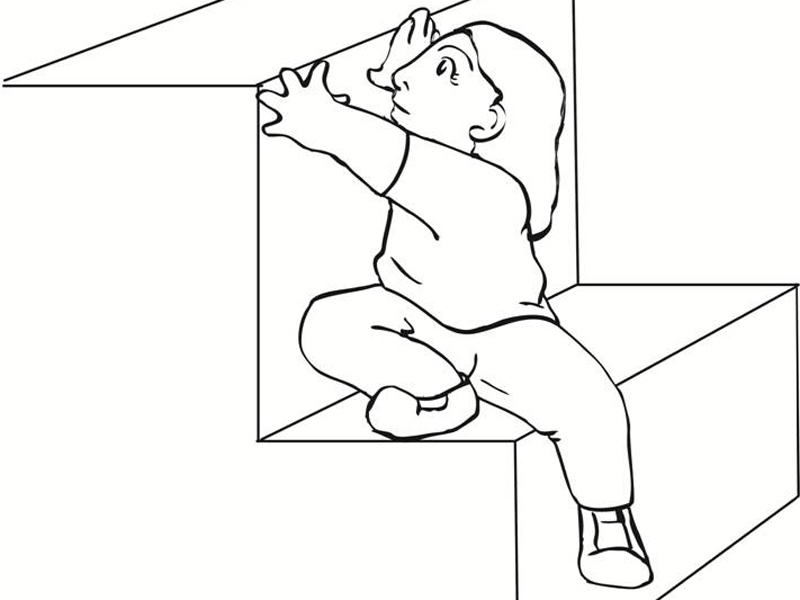

Supplement: Appendix S5 — All NeoHelp stimuli in JPEG format. All stimuli are provided as used in the study reported. (ZIP) [file pone.0084373.s005.zip › Stimuli/stair.jpg]

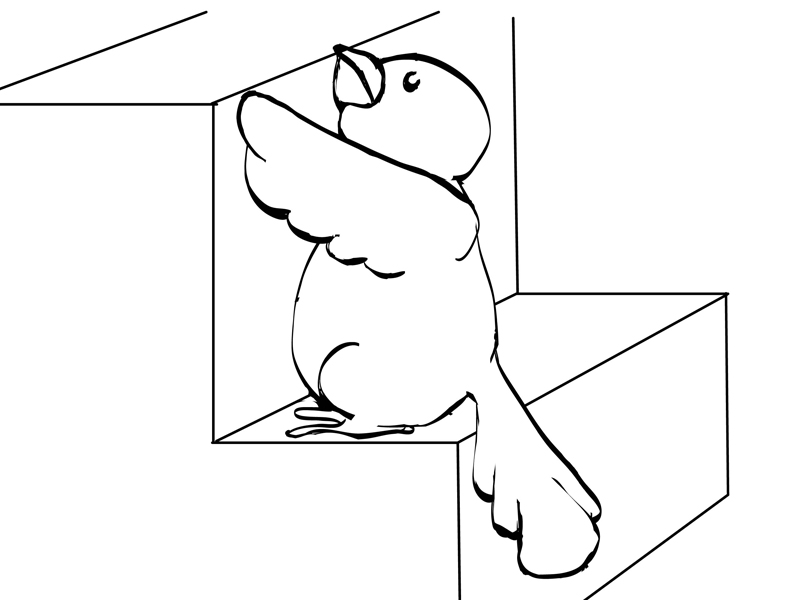

Supplement: Appendix S5 — All NeoHelp stimuli in JPEG format. All stimuli are provided as used in the study reported. (ZIP) [file pone.0084373.s005.zip › Stimuli/stair_bird.jpg]

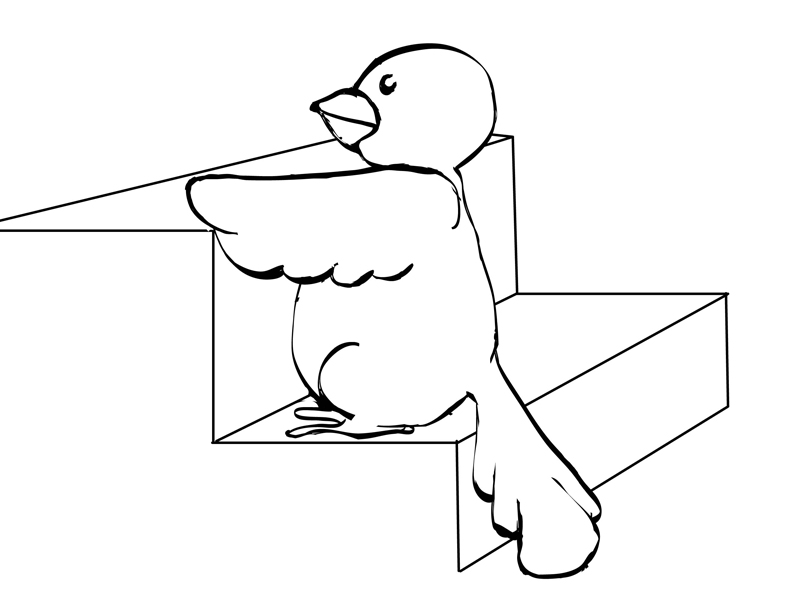

Supplement: Appendix S5 — All NeoHelp stimuli in JPEG format. All stimuli are provided as used in the study reported. (ZIP) [file pone.0084373.s005.zip › Stimuli/stair_bird_success.jpg]

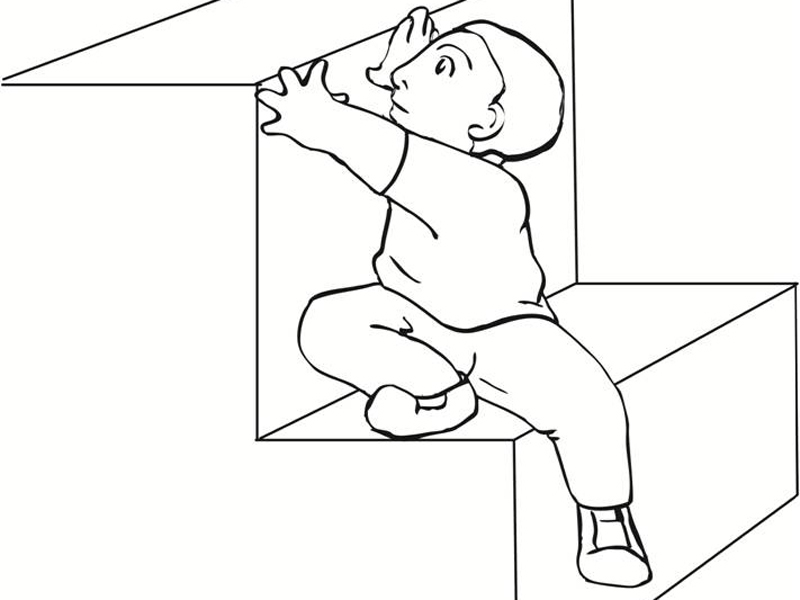

Supplement: Appendix S5 — All NeoHelp stimuli in JPEG format. All stimuli are provided as used in the study reported. (ZIP) [file pone.0084373.s005.zip › Stimuli/stair_boy.jpg]

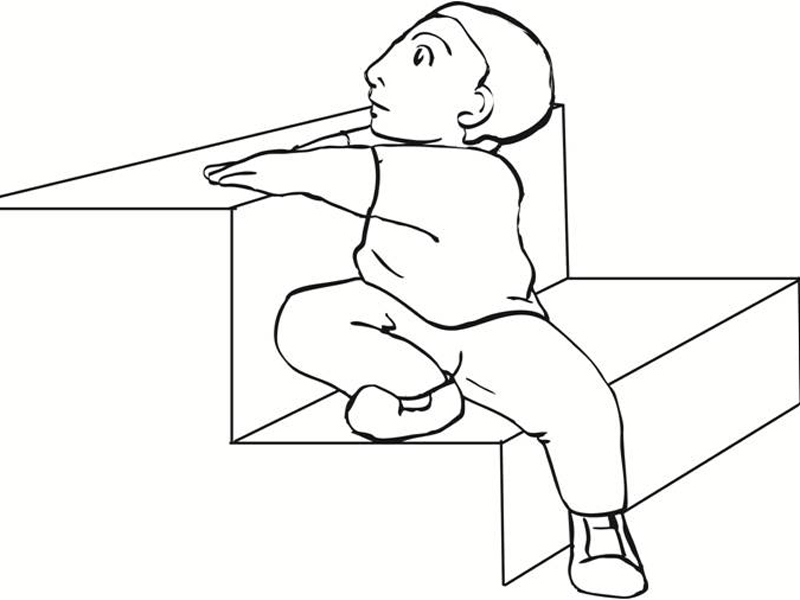

Supplement: Appendix S5 — All NeoHelp stimuli in JPEG format. All stimuli are provided as used in the study reported. (ZIP) [file pone.0084373.s005.zip › Stimuli/stair_boy_success.jpg]

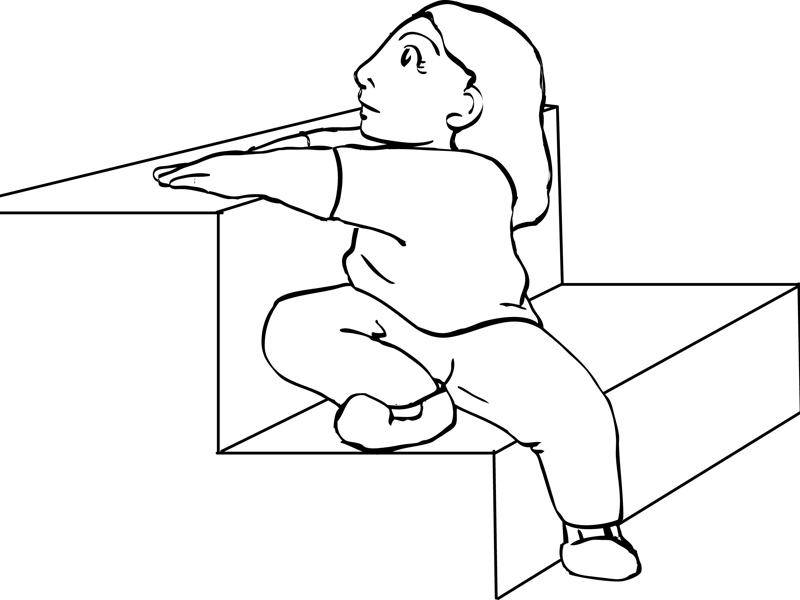

Supplement: Appendix S5 — All NeoHelp stimuli in JPEG format. All stimuli are provided as used in the study reported. (ZIP) [file pone.0084373.s005.zip › Stimuli/stair_success.jpg]

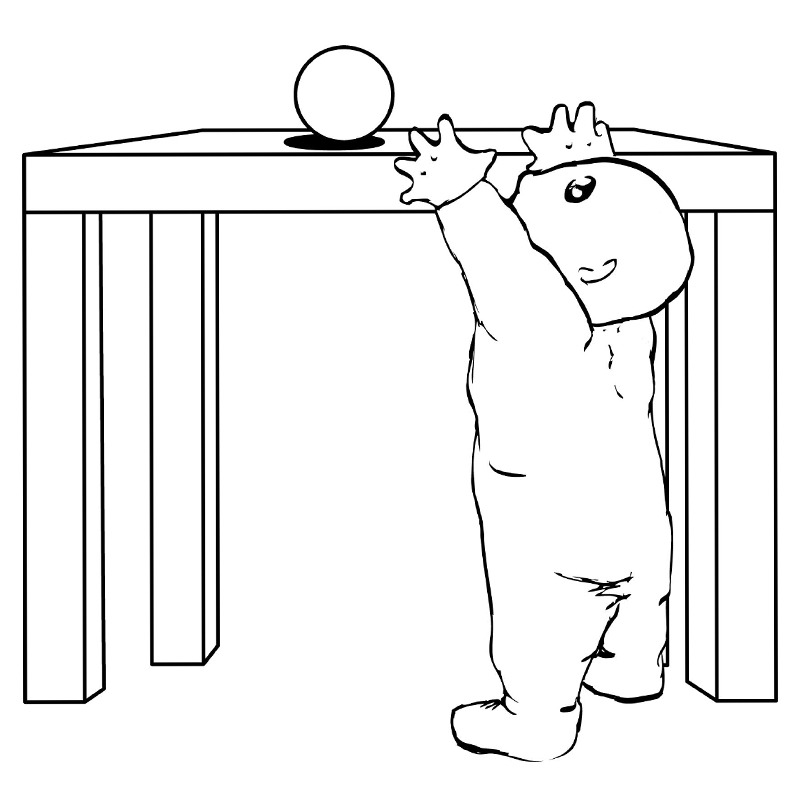

Supplement: Appendix S5 — All NeoHelp stimuli in JPEG format. All stimuli are provided as used in the study reported. (ZIP) [file pone.0084373.s005.zip › Stimuli/table.jpg]

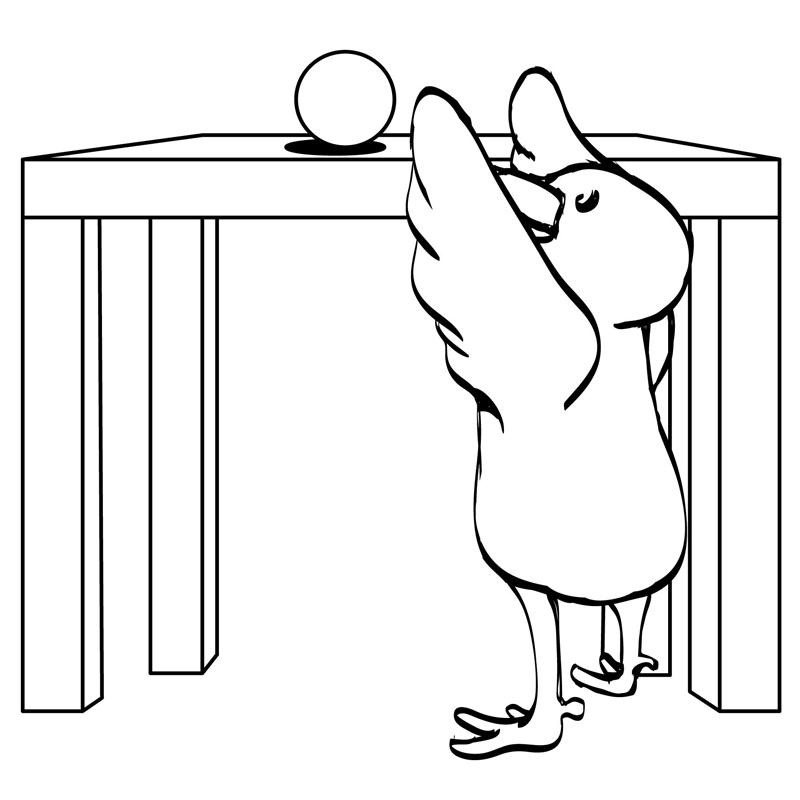

Supplement: Appendix S5 — All NeoHelp stimuli in JPEG format. All stimuli are provided as used in the study reported. (ZIP) [file pone.0084373.s005.zip › Stimuli/table_bird.jpg]

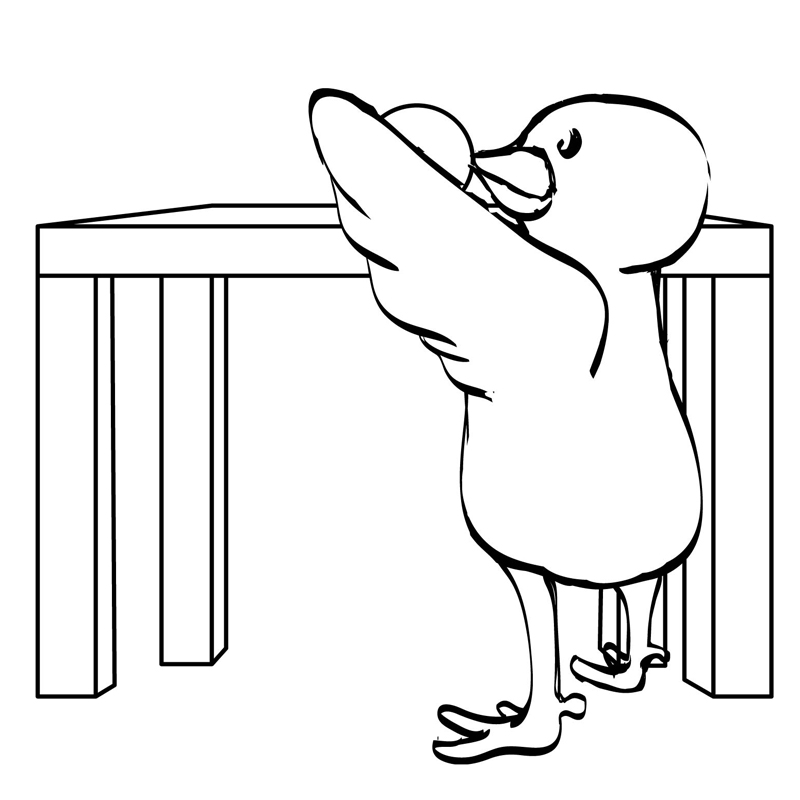

Supplement: Appendix S5 — All NeoHelp stimuli in JPEG format. All stimuli are provided as used in the study reported. (ZIP) [file pone.0084373.s005.zip › Stimuli/table_bird_success.jpg]

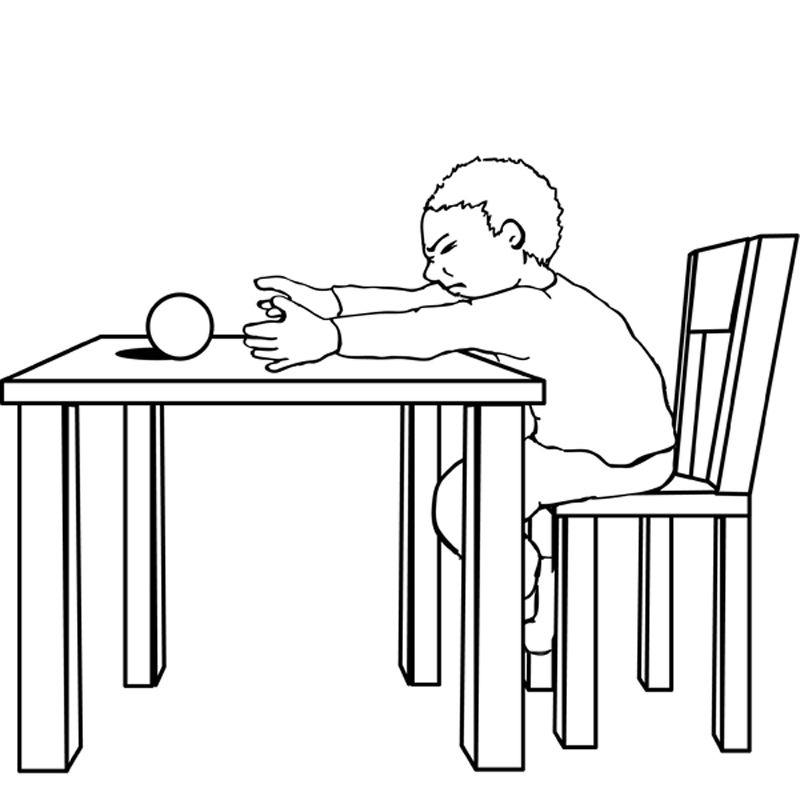

Supplement: Appendix S5 — All NeoHelp stimuli in JPEG format. All stimuli are provided as used in the study reported. (ZIP) [file pone.0084373.s005.zip › Stimuli/table_chair.jpg]

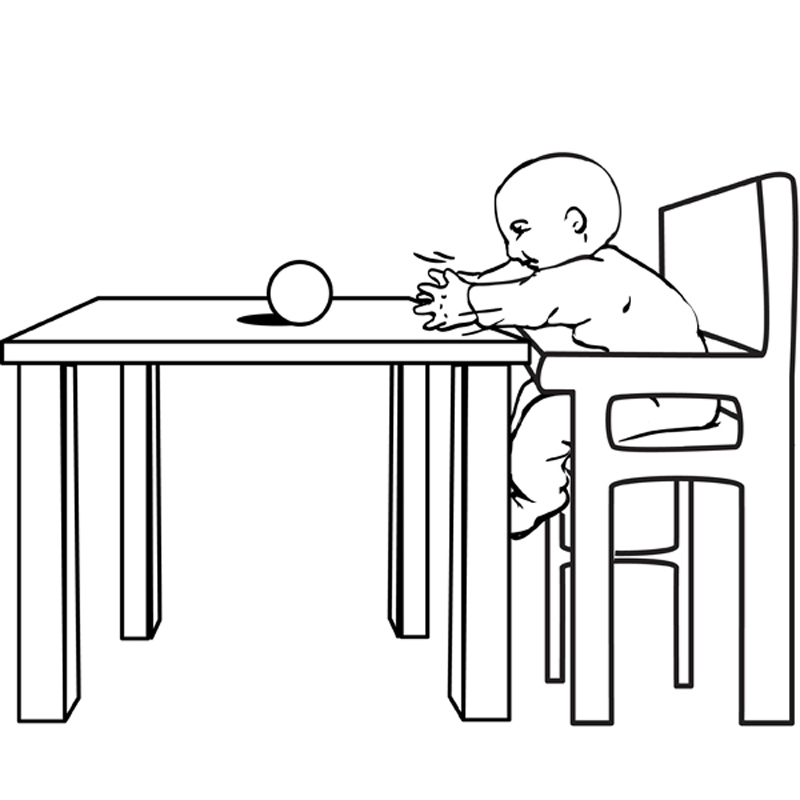

Supplement: Appendix S5 — All NeoHelp stimuli in JPEG format. All stimuli are provided as used in the study reported. (ZIP) [file pone.0084373.s005.zip › Stimuli/table_chair_6m.jpg]

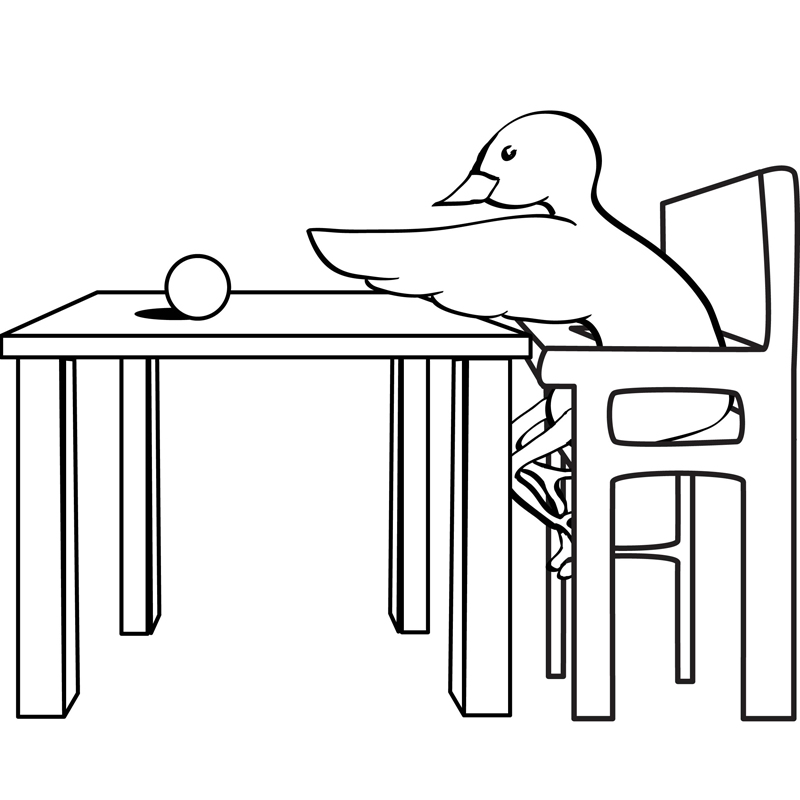

Supplement: Appendix S5 — All NeoHelp stimuli in JPEG format. All stimuli are provided as used in the study reported. (ZIP) [file pone.0084373.s005.zip › Stimuli/table_chair_6m_bird.jpg]

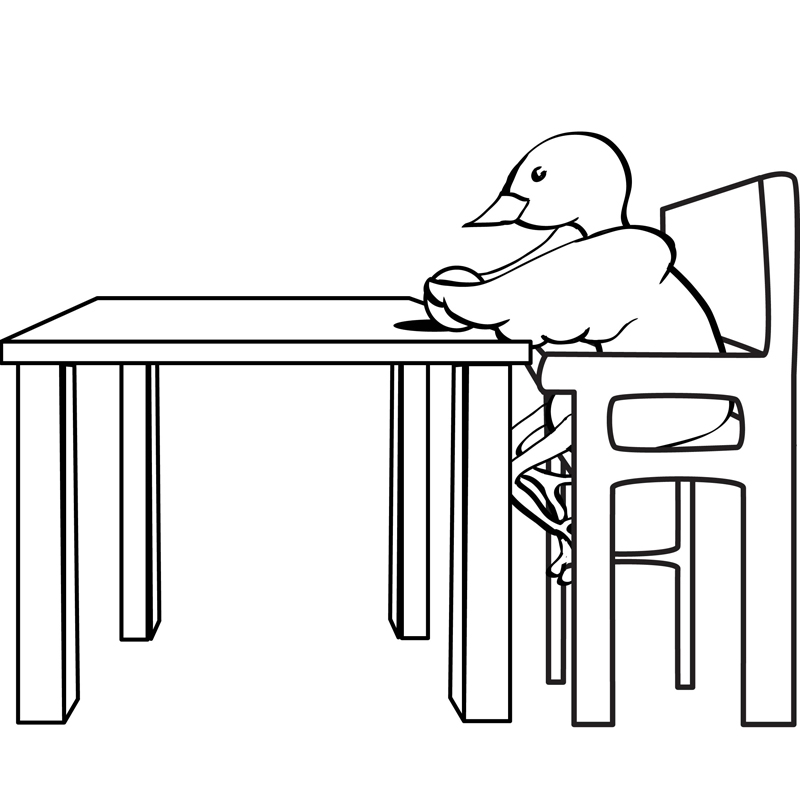

Supplement: Appendix S5 — All NeoHelp stimuli in JPEG format. All stimuli are provided as used in the study reported. (ZIP) [file pone.0084373.s005.zip › Stimuli/table_chair_6m_bird_success.jpg]

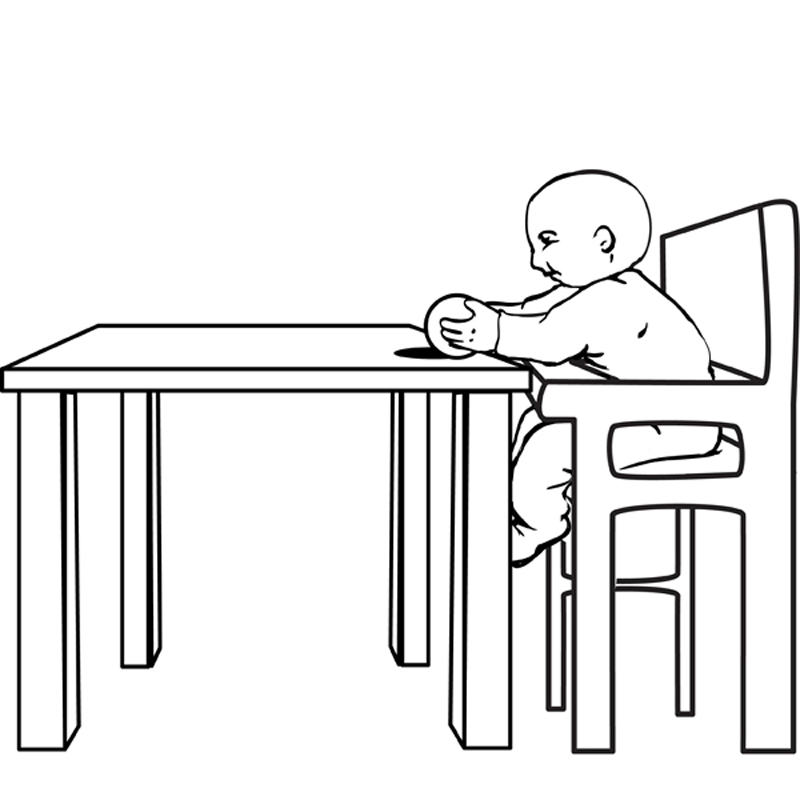

Supplement: Appendix S5 — All NeoHelp stimuli in JPEG format. All stimuli are provided as used in the study reported. (ZIP) [file pone.0084373.s005.zip › Stimuli/table_chair_6m_success.jpg]

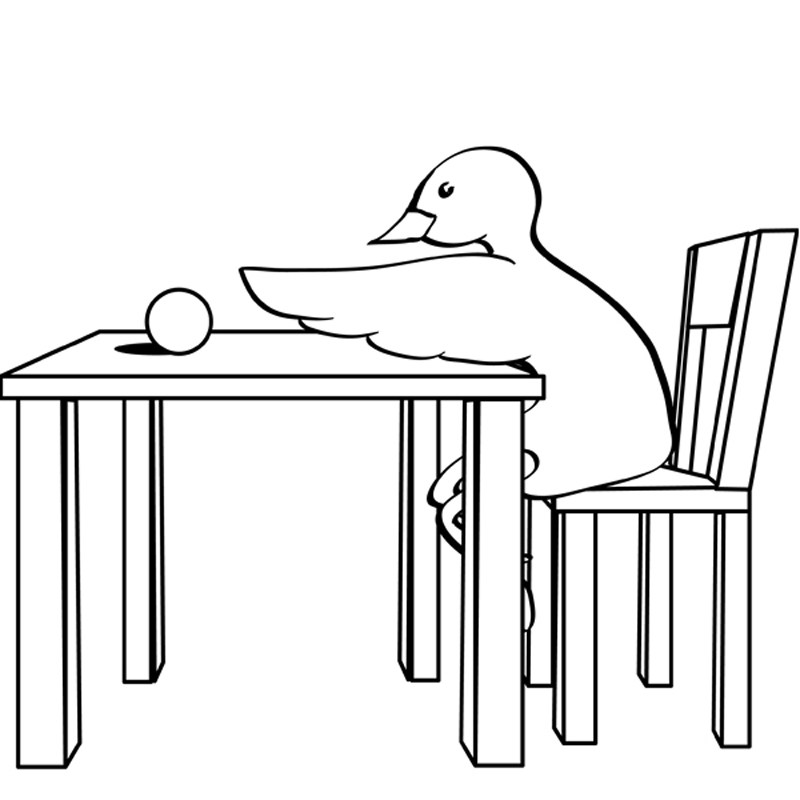

Supplement: Appendix S5 — All NeoHelp stimuli in JPEG format. All stimuli are provided as used in the study reported. (ZIP) [file pone.0084373.s005.zip › Stimuli/table_chair_bird.jpg]

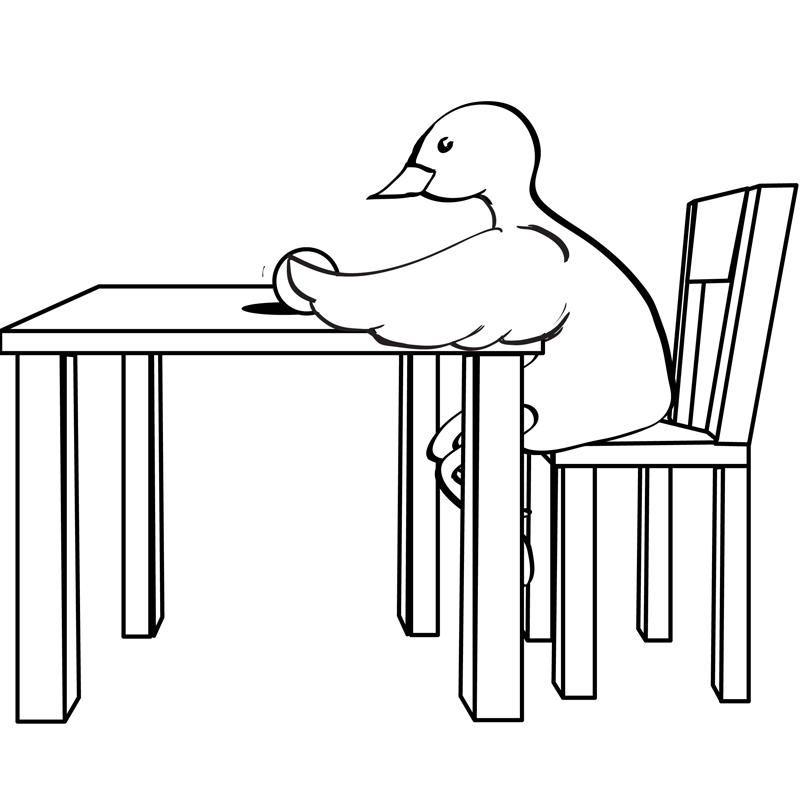

Supplement: Appendix S5 — All NeoHelp stimuli in JPEG format. All stimuli are provided as used in the study reported. (ZIP) [file pone.0084373.s005.zip › Stimuli/table_chair_bird_success.jpg]

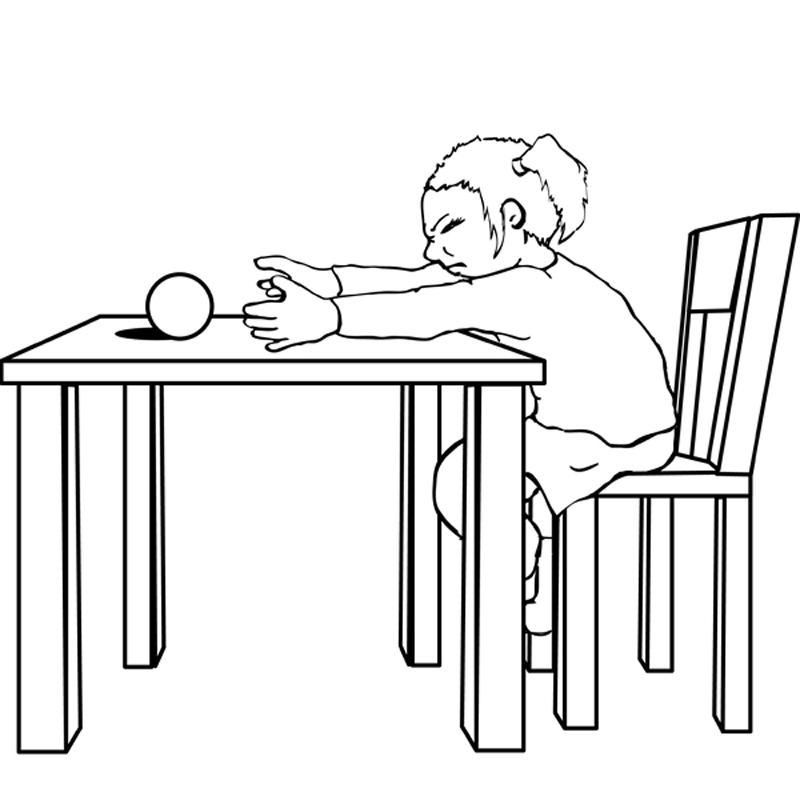

Supplement: Appendix S5 — All NeoHelp stimuli in JPEG format. All stimuli are provided as used in the study reported. (ZIP) [file pone.0084373.s005.zip › Stimuli/table_chair_girl.jpg]

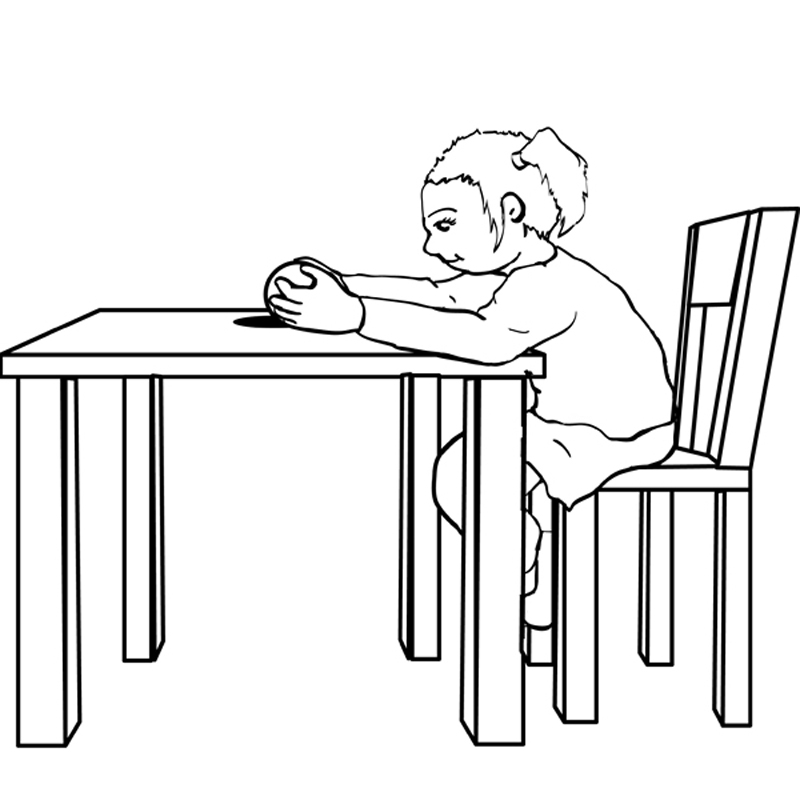

Supplement: Appendix S5 — All NeoHelp stimuli in JPEG format. All stimuli are provided as used in the study reported. (ZIP) [file pone.0084373.s005.zip › Stimuli/table_chair_girl_success.jpg]

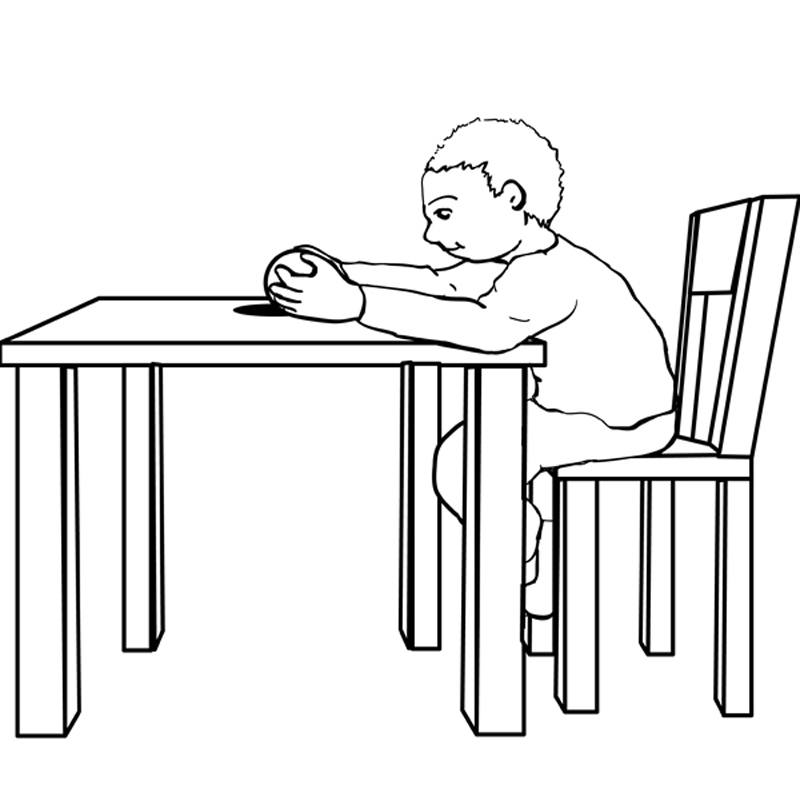

Supplement: Appendix S5 — All NeoHelp stimuli in JPEG format. All stimuli are provided as used in the study reported. (ZIP) [file pone.0084373.s005.zip › Stimuli/table_chair_success.jpg]

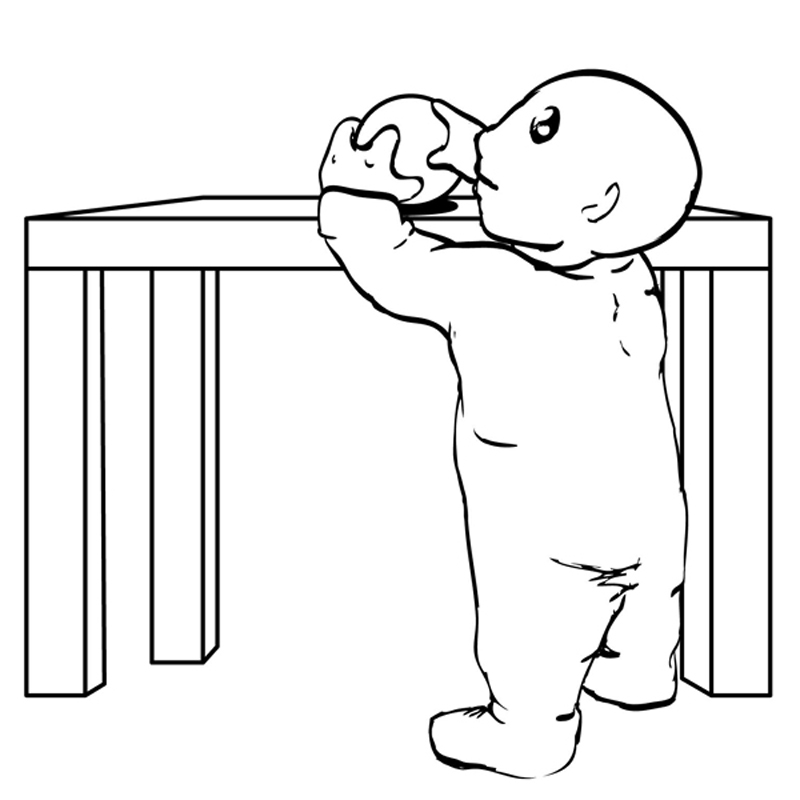

Supplement: Appendix S5 — All NeoHelp stimuli in JPEG format. All stimuli are provided as used in the study reported. (ZIP) [file pone.0084373.s005.zip › Stimuli/table_success.jpg]
